# Combining two relatively weak bases (Zn(TMP)_2_ and KO*t*Bu) for the regioselective metalation of non-activated arenes and heteroarenes†

**DOI:** 10.1039/d4sc03892d

**Published:** 2024-08-13

**Authors:** Neil R. Judge, Eva Hevia

**Affiliations:** a Departement für Chemie, Biochemie und Pharmacie, Universität Bern 3012 Bern Switzerland eva.hevia@unibe.ch

## Abstract

Co-operation between two relatively weak Brønsted bases, Zn(TMP)_2_ and KO*t*Bu, produces a bimetallic base strong enough to regioselectively zincate non-activated arenes such as naphthalene, biphenylene and anthracene under mild conditions. This co-operativity is also effective with a range of more sensitive five-membered ring heterocyclic substrates including benzoxazole and caffeine. Metalation products have been intercepted with iodine, affording the relevant iodo-(hetero)arenes in good to excellent yields with finely tuned regioselective control. Combining NMR spectroscopic and X-ray crystallographic studies has uncovered that depending on the solvent, a complicated ligand distribution process of mixed aryl/alkoxy higher order zincate intermediates, (THF)_*n*_K_2_Zn(Ar)_2_(O*t*Bu)_2_, that can liberate lower order zincates of the form [(THF)_2_KZn(Ar)(O*t*Bu)_2_]_2_ and eliminate potassium aryl species. While this ligand redistribution process seems to operate for non-substituted (hetero)arene metalation products, for non-activated alkylarenes such as mesitylene or *m*-xylene the higher-order zincates resulting from their lateral metalation are stable in solution and the solid state, which is attributed to the greater π-stabilisation that these systems can provide to the K cations. Adding another layer of complexity to this heterobimetallic system, over time the Zn(TMP)_2_/2KO*t*Bu combination reacts with the THF solvent of these reactions, to afford an unusual decomposition product which contains an *s-trans*-1,3-butadienyl (C_4_H_5_^−^) fragment coordinated to Zn within a potassium zincate structure. The formation of the latter is attributed to the initial synergistic α-zincation of THF, followed by subsequent ring opening and oxygen extrusion.

## Abstract 2

Combining KO*t*Bu with Zn(TMP)_2_ allows for the regioselective zincation of a wide range of non-activated arenes. Advancing mechanistic understanding, a new ligand redistribution process has been uncovered with important implication in the success of the Zn–H exchange process.

## Graphical abstract

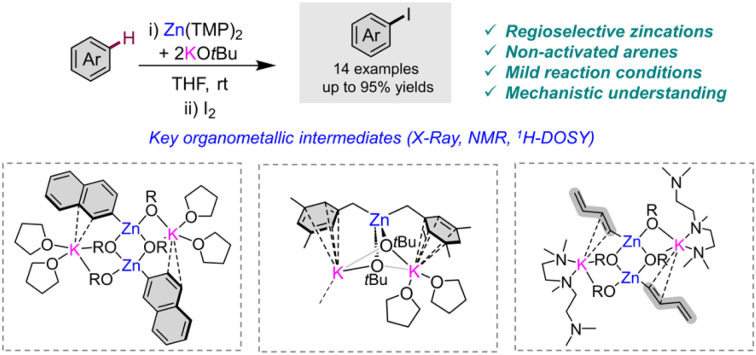

## Introduction

**Fig. 1:**
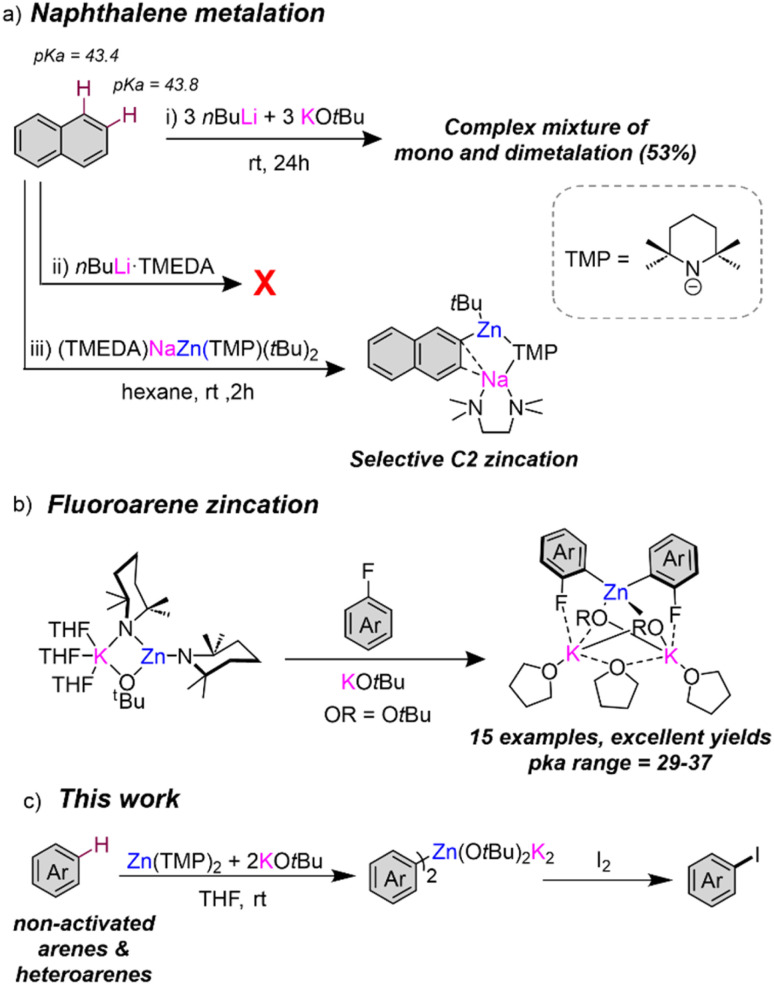
(a) Contrasting naphthalene metallation between conventional bases and a sodium zincate; (b) zincation of fluoroarenes using a Zn(TMP)_2_/2KO*t*Bu combination; (c) extension of the metallation scope of Zn(TMP)_2_/2KO*t*Bu to non-activated arene substrates.

Directed-*ortho* metalation (D*o*M) constitutes one of the most powerful and widely used synthetic methodologies for the regioselective functionalisation of aromatic rings.^1,2^ The regioselectivity and efficiency of such deprotonations is favoured by the presence of directing functional groups *ortho*-located to the C–H bond that experiences metallation.^3,4^ A dual activating effect has been noted. Thus, electron withdrawing directing groups (DGs) enhance the acidity of their vicinal *ortho*-H's but, in addition, the DG provides a coordination site for the metallating reagent which directs the *ortho*-regioselectivity of the process.^5–7^ Contrastingly, the regioselective metalation of non-activated arenes lacking a directing group is significantly more challenging,^2^ frequently requiring use of harsh reaction conditions and strong bases which often lead to low conversions and poor regioselectivities. This can be nicely illustrated for naphthalene, which possesses two equally non-activated sites for metallation with similar acidities in terms of p*K*_a_ (43.4 and 43.8 for C1 and C2 sites respectively).^8^ Employing *n*BuLi, Gilman has reported low conversions (up to 20%) of a mixture of C1- and C2-lithiated isomers in a 2.5 : 1 ratio respectively.^3^ The difficulties in controlling the selectivity in this reaction was emphasised further by Schlosser, who using bimetallic superbase *n*BuLi·KO*t*Bu reported a complex mixture of twelve different isomers of mono- and di-metallation in an overall moderate yield (53%) [Fig. 1a(i) and (ii)].^9^ Mulvey achieved far better regioselective control using sodium zincate [NaZn(TMP)*t*Bu_2_] as a bimetallic base, that induced C2 zincation of naphthalene under mild reaction conditions in good yields [Fig. 1a(iii)].^10^ With O'Hara, Mulvey has also reported selective 1,4-dimagnesiation of naphthalene using [Na_2_Mg(TMP)_3_*n*Bu] through a template deprotonation approach.^2,11,12^ These processes are described as sodium-mediated-metalations, with sodium proposed to play a prominent role in coordinating naphthalene *via* π-electrostatic interactions, favouring the low polarity metallation effected by Zn or Mg. Recent studies from our group have also shown the potential of NaTMP as a monometallic base in combination with the Lewis donor PMDETA (*N*,*N*,*N*′,*N*′′,*N*′′-pentamethyldiethylenetriamine) and a B(OiPr)_3_ as an *in situ* electrophilic partner to promote regioselective and high yielding C2 borylation of naphthalene to give a sodium boronate species that are amenable to Suzuki–Miyaura coupling reactions.^13^

In parallel with this work, our group has uncovered the profound activating effects of alkali-metal alkoxide additives towards enhancing the reactivities of organomagnesium^14,15^ and organozinc reagents.^16,17^ Reminiscent of the aforementioned *n*BuLi·KO*t*Bu superbase, we reported the activating effects of adding stoichiometric amounts of KO*t*Bu to the zinc bis-amide Zn(TMP)_2_ which permits direct zincation of a broad range of fluoroarenes at room temperature with excellent regioselective control (Fig. 1b). This is particularly remarkable since, differing from Mulvey's bimetallic combinations, both single-metal components KO*t*Bu and Zn(TMP)_2_, are relatively weak bases that on their own are inert towards fluoroarene metallation. Mechanistic investigations have shown that the reaction occurs by formation of the unique, highly reactive alkoxy/amido potassium zincate [KZn(TMP)_2_O*t*Bu].^16^ These studies also revealed that in order to control the speciation of the organometallic species present in solution, an additional equivalent of KO*t*Bu was essential to induce formation of the higher order potassium zincate [K_2_Zn(Ar)_2_(O*t*Bu)_2_] which proved isolable for X-ray structural refinement. The potential of this bimetallic combination for zincation of the non-activated substrates benzene and toluene was realised by promising results although in these cases the substrates were employed as the bulk solvents of the reaction.^16^

Expanding further the synthetic potential of this bimetallic combination here we investigate its applications for the regioselective zincation of non-activated substrates devoid of directing groups, as well as of a selection of key heterocyclic molecules. Aiming to shed light on the activating effect provided by KO*t*Bu, the roles of the alkali-metal and donor ability of the solvents employed are considered as well as the identity of the metalated intermediates prior to electrophilic insertion, which has revealed their complicated multi-species constitution in solution. The stability of this Zn(TMP)_2_/2KO*t*Bu mixture has also been evaluated in different solvents revealing its ability to cleave THF in an unusual manner to form a butadienyl fragment that can be trapped within a zincate structure.

## Results and discussion

We began the study using naphthalene 1a as a model substrate assessing the selectivity and extent of metalation *via* an iodine quench after reaction with assorted bases in THF (Table 1). Unsurprisingly, Zn(TMP)_2_ completely fails to promote any metalation of 1a in the absence of alkoxide additives with complete recovery of naphthalene observed even under refluxing conditions (entry 1). In stark contrast, adding two equivalents of KO*t*Bu (entry 2) led to the quantitative and selective C2-zincation of two equivalents of 1a in just 2 h at room temperature forming 2-iodonaphthalene which could be isolated in an 89% isolated yield. These findings contrast with our preliminary studies assessing the zincation of toluene and benzene with this basic combination which required the use of the non-activated arene as bulk solvents.

**Table 1:** Screening of naphthalene (1) C2-zincation by different Zn(TMP)_2_/2(AM)O*t*Bu (AM = Li, Na, K) pairings and subsequent iodine quench forming 2-iodonaphthalene (2a)

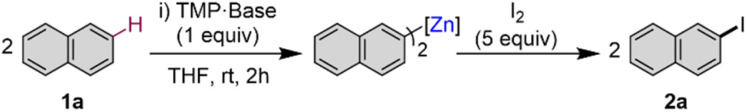		
Entry	Base	Yield^a^ (%)
1	Zn(TMP)_2_	0^b^
**2**	**Zn(TMP)**_**2**_ **+ 2KO*t*Bu**	**99 (89)** c
3	Zn(TMP)_2_ + 2LiO*t*Bu	0
4	Zn(TMP)_2_ + 2NaO*t*Bu	0
5	Zn(TMP)_2_ + 2KO*t*Bu + 2(18-crown-6)	0
6	KTMP	<5%^d^
7	Zn(TMP)_2_·2MgCl_2_·2LiCl	<5%

a Conversions determined by ^1^H NMR monitoring using hexamethylbenzene as internal standard.

b Reaction refluxed for 2 h.

c Isolated yield after purification by column chromatography.

d 1 equivalent of naphthalene used.

A significant alkali metal effect is then observed using the lighter alkali metal *tert*-butoxide congeners, LiO*t*Bu (entry 3) or NaO*t*Bu (entry 4) which completely shuts down the reactivity of the bimetallic mixture towards 1, since no zincation occurs. This is particularly surprising for Na since previous work by Mulvey discovered that sodium zincates and magnesium bimetallics can promote the metalation of naphthalene (*vide supra*). Here, the softer and larger K centres can be envisaged as built-in Lewis acidic centres which can π-engage with the naphthalene ring activating the substrate towards C–H zincation. In this regard, Pardue *et al.* probed the benzylic deprotonation of toluene by alkali metal amides *via* DFT calculations concluding that the aforementioned cation–π interactions facilitate the C–H bond scission finding that the heaviest alkali metal Cs amide offers the lowest energy barrier for this transformation.^18^ The importance of these K–π interactions for successful zincation of naphthalene is discernible from entry 5 in Table 1 where the same reaction is carried out in the presence of two equivalents of 18-crown-6, which in forming a solvent-separated ion pair sequesters and coordinatively saturates the K cations, presumably precluding precoordination of the arene substrate. Monometallic potassium amide KTMP also failed to promote the metalation of naphthalene in THF at room temperature (entry 6) highlighting the importance of a bimetallic system for this transformation. It should also be noted that under these conditions, other bimetallic bases which have previously shown considerable promise for arene, or benzylic metalation also fail to metalate naphthalene to any appreciable extent. This includes the LiNK reagent,^19^ Mongin's tandem LiTMP/Zn(TMP)_2_ combination^20^ (see ESI†) and Knochel's powerful Turbo Hauser zinc reagent Zn(TMP)_2_·2MgCl_2_·2LiCl^21^ (entry 7).

**Fig. 2:**
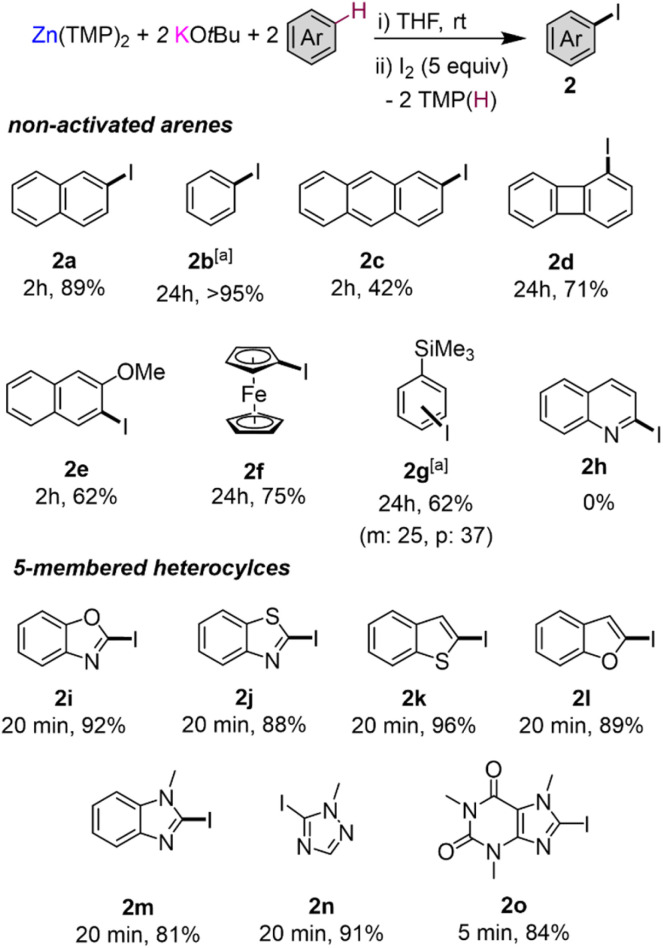
Scope of the C–H zincation and subsequent iodination using a 2 : 1 mixture of KO*t*Bu/Zn(TMP)_2_ forming iodoarenes 2a–2o. Substrate (2 equiv.), KO*t*Bu (2 equiv.) and Zn(TMP)_2_ (1 equiv.) in THF followed by I_2_ quench (5 equiv.). Isolated yields. ^*a*^Substrate used as a solvent and yields of 2b and 2g determined using hexamethylbenzene as an internal standard.

To assess the scope of the optimised conditions [Zn(TMP)_2_/2KO*t*Bu in THF] we introduced other non-activated arenes to the study (Fig. 2). Iodobenzene 2b was produced quantitatively after stirring a solution of Zn(TMP)_2_/2KO*t*Bu in neat benzene for 24 h at room temperature prior to an iodine quench. Demonstrating the strong metalating power of this K/Zn heterobimetallic combination, the high yielding zincation of benzene under mild conditions contrasts with recent studies using Pd-catalysis to promote C–H zincation of benzene which requires 5 days at 100 °C under static vacuum.^22^ Moving to larger π-extended systems, reports of selective deprotonative metalation and functionalisation of anthracene are extremely scarce,^13^ though here, a 42% yield of 2-iodoanthracene 2c could be obtained *via* the zincation of two equivalents of anthracene at room temperature after just 2 hours. Unfortunately, extension to other π-extended systems, such as pyrene and phenanthrene, proved less successful forming deep coloured NMR silent^23^ solutions indicative of competitive single electron transfer (SET) processes.^24^ Indeed, this competing SET process seems to be apparent for the zincation of anthracene which explains the moderate yield of 2c (see ESI for details†). Next, when two equivalents of biphenylene were reacted with Zn(TMP)_2_/2KO*t*Bu in THF at room temperature, a quantitative C1 zincation is achieved after 24 h (confirmed by ^1^H NMR monitoring) forming 1-iodobiphenylene 2d in a 71% isolated yield after an iodine quench and purification. Previous reports of the direct metalation of biphenylene are scarce, though Johnson noted C1-lithiation of biphenylene using a 4 fold excess of *t*BuLi under cryogenic conditions (−78 °C) but over the course of 3 days.^25^ O'Hara has successfully used the aforementioned [Na_2_Mg(TMP)_3_*n*Bu] template base for 1,4-dimagnesiation of biphenylene.^12^ Our K/Zn bimetallic base combination also reacts smoothly with two equivalents of 2-methoxynaphthalene affording a 62% yield of 3-iodo-2-methoxynaphthalene 2e, post electrophilic interception. This regioselectivity mirrors that previously observed using the *n*BuLi·KO*t*Bu superbase where an exclusive C3 metallation is also observed, although low temperatures (−78 °C) need to be employed.^26^ 1-Iodoferrocene 2f can be isolated in a 75% yield after selective monozincation of ferrocene with Zn(TMP)_2_/2KO*t*Bu for 24 h followed by iodination. Note that while alkali-metal zincates have been previously used for ferrocene zincation,^27–30^ they tend to promote polymetalation reactions, even when working under stoichiometric conditions, making the isolation of monometalated species particularly challenging. Interestingly, the zincate bases used in these studies are made by pairing dialkylzinc reagents with strong Group 1 metal amide TMP-bases, whereas for 2f, the bimetallic reagent comprises two weakly basic components KO*t*Bu and Zn(TMP)_2_ that separately are completely inert towards ferrocene metalation, demonstrating the cooperative origin of this reactivity. Zincation of trimethyl(phenyl)silane could be achieved in a 65% yield after iodination, affording 2g as a 1 : 1.5 mixture of the products of *meta*- and *para*-iodination, however the arene was used as the reaction solvent in this case. This regioselectivity seems to be directed by the steric demands of the SiMe_3_ group which precludes *ortho*-zincation.^9,31^ Similar regioselectivities have been previously reported by Schlosser for deprotonation of triisopropyl(phenyl)silane using a combination of *n*BuLi·KO*t*Bu (1 equiv. base) for 24 h at room temperature giving rise to a 58% yield of *m*/*p* isomers in a 28 : 72 ratio.^9^

Attempts to extend our approach to pyridines and diazines were unsuccessful as extensive decomposition was seen at room temperature, as shown in Fig. 2 for quinoline 2h. Contrastingly, effective alpha-zincation of a range of more activated five-membered heterocyclic molecules such as benzoxazole, benzothiazole, benzothiophene, benzofuran, *N*-methyl imidazole, 1-methyl-1,2,4-triazole and caffeine was successfully achieved affording the relevant alpha-C-iodination products 2i–2o in excellent yields ranging from 81 to 96%. Reactions could be carried out at room temperature in short periods of time (5–20 min), without observing side reactions or decomposition of the relevant metalated intermediates. This method complements that reported by Daugulis for halogenation of electron-rich five-membered ring heterocycles, using LiO*t*Bu or K_3_PO_4_ as base, which requires a large excess of base (2–4 equivalents) and elevated temperatures (50–130 °C) for extended periods of time (10–13 h) using DMF as solvent.^32^ In addition, the zincated intermediates from these reactions can also engage in Pd catalysed cross-coupling reactions as shown for benzothiophene which can be metalated *in situ* and cross-coupled with 4-iodoanisole in the presence of 5 mol% of [Pd(OAc)_2_]_3_ to furnish 2-(4-methoxyphenyl)benzothiophene in almost quantitative yield (see ESI for details†).

**Fig. 3:**
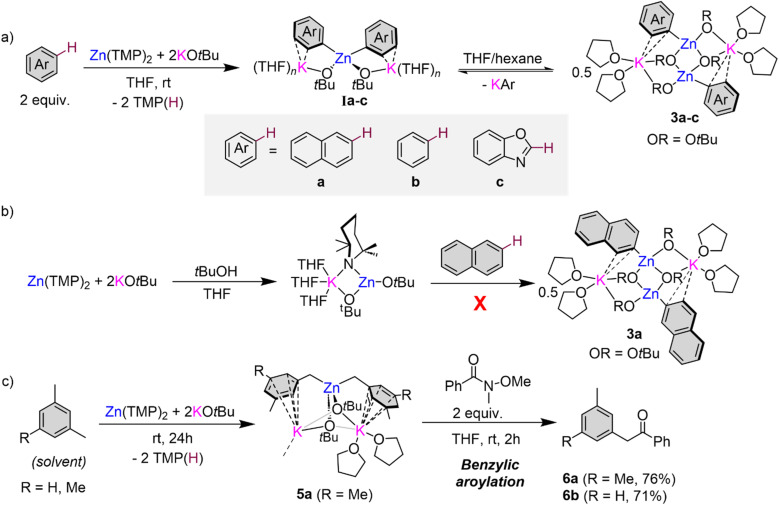
(a) Formation of lower order potassium zincates 3a–c*via* redistribution of higher order potassium zincates I; (b) *in situ* formation of (THF)_*n*_KZn(TMP)(O*t*Bu)_2_ and reaction with naphthalene; (c) benzylic zincation of mesitylene using a 2 : 1 mixture of KO*t*Bu/Zn(TMP)_2_ forming [(THF)_2_K_2_Zn(Ar)_2_(O*t*Bu)_2_]_∞_5 (Ar = 3,5-dimethylbenzyl) followed by addition to a Weinreb amide affording acetophenones 6.

**Fig. 4:**
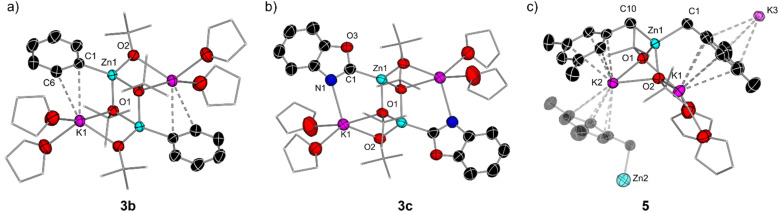
(a) Molecular structure of [(THF)_2_KZn(Ph)(O*t*Bu)_2_]_2_ (3b) with displacement ellipsoids at 50% probability, all H atoms omitted and with C atoms in the alkoxide substituent and THF molecules drawn as wire frames for clarity; (b) molecular structure of [(THF)_2_KZn(C2-benzoxazolyl)(O*t*Bu)_2_]_2_ (3c) with displacement ellipsoids at 50% probability, all H atoms omitted and with C atoms in the alkoxide substituent and THF molecules drawn as wire frames for clarity; (c) molecular section of [(THF)_2_K_2_Zn(CH_2_-3,5-Me_2_-C_6_H_3_)_2_(O*t*Bu)_2_]_∞_ (5) with displacement ellipsoids at 50% probability, all H atoms omitted and with C atoms in the alkoxide substituent and THF molecules drawn as wire frames for clarity.

To gain a better understanding on how these zincation reactions take place, we turned to trapping and characterising metalated intermediates that could be key in these transformations. Our previous studies assessing the potential of this basic combination for the zincation of fluoroarenes have shown that the zincated intermediates are stabilised by the formation of K⋯F contacts that also seem to have a strong directing effect, controlling the regioselectivity of the Zn–H exchange process (Fig. 1b).^16^ Taking benzene as a model substrate, ^1^H-NMR monitoring studies are consistent with the formation of higher order potassium zincate [K_2_Zn(Ph)_2_(O*t*Bu)_2_] (Ia) (Fig. 3a), while its ^13^C{^1^H} NMR spectrum in d_8_-THF exhibits an informative signal at 171.3 ppm for its Zn–C(Ph) fragment. ^1^H DOSY NMR studies additionally showed that the Ph and O*t*Bu groups in Ib belong to the same molecular entity (Fig. S36 in ESI†). Endeavours to isolate Ib as a crystalline solid from hexane/THF solvent mixtures led to the repeated crystallisation of lower order potassium zincate [(THF)_2_KZn(Ph)(O*t*Bu)_2_]_2_ (3b) (Fig. 3a and 4a) in a 23% crystalline yield.

Exhibiting a centrosymmetric dimeric structure, 3b features a ladder motif comprising outer K–O rungs [K1–O1, 2.830(11) Å] and inner Zn–O rings [Zn1–O2, 2.062(2) Å]. Alternatively, 3b can be envisaged as two {KOZnO} rings which combine *via* their Zn–O edges to generate a tetrametallic ladder. Within this motif, one Ph group binds terminally to Zn, which occupies the position previously filled by a H atom, forming a sigma Zn–C bond [2.033(15) Å], whereas each K is disposed perpendicularly to the aromatic ring, π-engaging with two of its carbons [K1–C1 3.178(15) Å and K1–C6 3.194(19) Å] and completing its coordination sphere *via* two molecules of THF. This ladder motif is reminiscent to that reported by our group for mixed alkyl/alkoxy lithium magnesiates.^14,15^ NMR characterisation of 3b in d_8_-THF solutions display three informative signals at 7.89, 7.00 and 6.89 ppm for the *ortho*, *meta*, and *para*-H of the Ph ring in the ^1^H NMR spectrum and a resonance for the Zn–C_*ipso*_ at 167.4 ppm in the ^13^C NMR spectrum. ^1^H DOSY NMR studies also confirmed that both O*t*Bu and Ph groups belong to the same molecular entity, although in this case the relative integration of these two groups is 2 : 1 whereas for Ib the ratio is 1 : 1 (see ESI for details†).

Formation of 3b can be rationalised as the product of a ligand redistribution process of Ib with concomitant elimination of phenyl potassium (Fig. 3a). Previous studies on mixed alkyl (or aryl/alkoxide) alkali-metal magnesiates have already established the ability of these heterobimetallic systems to engage in complex equilibria between lower and higher order bimetallic species.^15^ The concomitantly formed KPh is presumed to decompose rapidly in THF and indeed Schlosser has previously documented the fragility of potassium aryl moieties in ethereal solvents such as THF.^33^ Whilst only isolated in a 23% crystalline yield (of a possible maximum 50% yield), analysis of the filtrate of 3b confirmed the presence of remnant [K_2_Zn(Ph)_2_(O*t*Bu)_2_] (Ib). It should be noted that higher order zincate (Ib) is perfectly stable in a solution of THF, and this redistribution process is only observed upon cooling a hexane/THF solution of this species to −30 °C for crystallisation. We attribute this to the low solubility of KPh under these conditions that drives the equilibrium shown in Fig. 3a towards formation of 3b.

Attempts to isolate zincation intermediates from the reaction of Zn(TMP)_2_/2KO*t*Bu in THF with naphthalene and benzoxazole by cooling THF/hexane solutions at −30 °C led to the isolation of [(THF)_2_KZn(C2-naphthyl)(O*t*Bu)_2_]_2_ (3a) and [(THF)_2_KZn(C2-benzoxazolyl)(O*t*Bu)_2_]_2_ (3c) in 29% and 24% crystalline yields respectively, demonstrating that the presence of this redistribution process is not exclusive to benzene (Fig. 3a and 4). It should be mentioned that when using fluoroarenes (Fig. 1b)^16^ formation of related [(THF)_2_KZn(Ar^F^)(O*t*Bu)_2_] species resulting from a similar redistribution process was never observed. It is important to stress that 3a could not be accessed by direct zincation of naphthalene with zincate [(THF)_*n*_KZn(TMP)(O*t*Bu)_2_] which was prepared *in situ* by combining [(THF)_*n*_KZn(TMP)_2_(O*t*Bu)] with one equivalent of *t*BuOH (Fig. 3b). This lack of reactivity supports that formation of 3a is a post-metalation event. These findings also emphasize the importance of the TMP : O*t*Bu ratio present in the base in order to promote the zincation reactions.

**Fig. 5:**
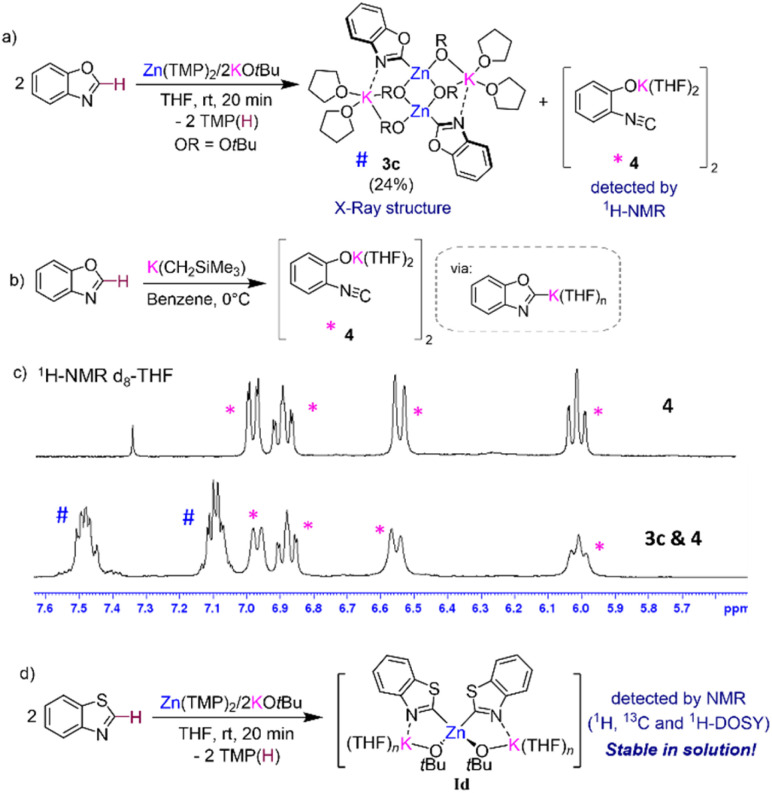
(a) Formation of [(THF)_2_KZn(C2-benzoxazolyl)(O*t*Bu)_2_]_2_ (3c) and [K(1,2-*O*-C_6_H_4_-NC)] (4) *via* reaction of Zn(TMP)_2_/2KO*t*Bu with two equivalents of 1,3-benzoxazole; (b) rationale synthesis of [K(1,2-*O*-C_6_H_4_-NC)] (4); (c) stacked ^1^H-NMR of [K(1,2-*O*-C_6_H_4_-NC)] (4) (top) and a mixture of [(THF)_2_KZn(C2-benzoxazolyl)(O*t*Bu)_2_]_2_ (3c) and [K(1,2-*O*-C_6_H_4_-NC)] (4) (bottom); (d) formation of higher order zincate [(THF)_*n*_K_2_Zn(C2-benzothiazolyl)_2_(O*t*Bu)_2_] (Id) *via* reaction of Zn(TMP)_2_/2KO*t*Bu with two equivalents of 1,3-benzothiazole.

Interestingly, ^1^H-NMR monitoring of the zincation of benzoxazole revealed that in this case, formation of 3c occurs almost instantaneously at room temperature in THF solution. However, now the concomitant [K(C2-benzoxazolyl)] species does not react with the solvent, instead it undergoes ring opening to form potassium phenoxide [(THF)_2_K(1,2-*O*-C_6_H_4_-NC)]_2_ (4) in d_8_-THF solutions (Fig. 5a and c). Formation of 4 aligns well with previous studies on the lithiation or magnesiation of oxazoles that have revealed poor stability of the s-block metalated intermediates and their tendency to undergo ring opening.^34–37^ Compound 4 could be prepared independently by reacting KCH_2_SiMe_3_ with benzoxazole in THF (Fig. 5b) and its dimeric constitution in THF-solutions could be established with the aid of ^1^H-DOSY NMR (see ESI for details, Fig. S41†). The ^13^C NMR spectrum of 4 in d_8_-THF displays an informative signal at 117.4 ppm for the N*C* group, noticeably more upfield than that observed for the Zn–*C* of 3c which resonates at 197.9 ppm. ^1^H-NMR analysis of 4 could unambiguously confirm its presence, alongside potassium zincate 3c, in the *in situ* reaction mixture between Zn(TMP)_2_/2KO*t*Bu and two equivalents of 1,3-benzoxazole in d_8_-THF solution (Fig. 5c). Despite the formation of 4, quenching with iodine gives almost quantitative formation of 2i (Fig. 2). A control reaction of potassium enolate 4 with excess I_2_ led to the quantitative formation of the ring closed product 2-iodobenzoxazole 2i, which suggests a faster equilibration between ring opened enolate 4 and ring closed [K(C2-benzoxazolyl)] than the quenching of the opened isomer 4. It is also possible that in the presence of this electrophile the equilibration between Ic, 3c, and 4 is faster than the trapping of 3c and 4 with I_2_. Care must therefore be taken with the choice of electrophile for the reaction of Zn(TMP)_2_/2KO*t*Bu with benzoxazole as Jutzi has previously shown that lithiation of this heterocycle with *n*BuLi followed by a quench with trimethylsilylchloride (TMSCl) resulted in the formation of the ring opened product (2-isocyanophenoxy)trimethylsilane.^38^ Interestingly, NMR monitoring (including ^1^H-DOSY NMR) of reaction between two equivalents of the more robust heterocycle, 1,3-benzothiazole,^36^ with our Zn(TMP)_2_/2KO*t*Bu combination led to the quantitative zincation of the arene and the formation of higher order potassium zincate [(THF)_*n*_K_2_Zn(2-benzothiazolyl)_2_(O*t*Bu)_2_] (Id) as the sole metalation product with no redistribution of the metalated product observed in d_8_-THF solutions (see ESI for details†). Adding a further level of complexity, these findings suggest that the equilibria between the metalation product, higher-order zincate I, and the relevant lower order zincate 3 and the KAr do not only depend on the solvent employed but also on the nature of the aromatic substrate used.

The molecular structures of 3a and 3c were established by X-ray crystallographic studies (Fig. S1† and 4c). In both cases the same dimeric motif as that described for 3b is found, while for 3a the K atoms π-engage in an η^2^ fashion to two C atoms in the metalated arene ring in a similar manner to that observed for 3b with closely comparable geometrical parameters. However, in 3c the K atoms are stabilised by binding to the N atoms of the benzoxazolyl groups [K1–N1, 2.801(3) Å] rather than forming π-contacts with the aromatic ring as shown for 3a and 3b. The Zn–C bond distance in 3c, Zn1–C1 [2.007(3) Å], compares well with that previously reported by Boche for [(C2-benzoxazolyl)ZnCl(THF)]_2_ prepared by salt-metathesis of the relevant lithiated species with ZnCl_2_.^39^ Collectively these findings highlight the convoluted solution chemistry of the organometallic intermediates involved in these reactions, which can be profoundly influenced by the solvent and temperature of the reaction, leading in some cases to the formation of decomposition products that can ultimately impact on the yield of the organic product after electrophilic interception.

While proposed intermediates of type I (Fig. 3a) could not be structurally defined in the solid state for any of the model substrates investigated, using mesitylene as a solvent led to the isolation of higher order potassium zincate [(THF)_2_K_2_Zn(CH_2_-3,5-Me_2_-C_6_H_3_)_2_(O*t*Bu)_2_]_∞_ (5) in a 64% yield (Fig. 3c). X-ray crystallographic studies established its polymeric structure (Fig. 4c and S2†). The central Zn atom bonds to two benzylic carbon anions, Zn1–C1 [2.104(2)] Å and Zn1–C10 [2.102(2) Å], and to two *tert*-butoxide anions which bridge to the two potassium atoms present. The two potassium cations then connect by two alkoxide bridges. K1 satisfies its coordination sphere binding to two THF molecules and π-engaging in an η^5^ fashion with the π cloud of one mesityl anion [K⋯C interactions ranging from 3.382(2) to 3.536(3) Å]. K2 forms similar π interactions with the second mesityl group in the monomeric unit in an η^3^ fashion [K⋯C interactions ranging from 3.135(19) to 3.343(2) Å] however, K2 also π-engages in an η^4^ manner [K⋯C interactions ranging from 3.117(2) to 3.331(2) Å] with an additional mesityl group of a neighbouring unit propagating the two-dimensional polymeric structure (see Fig. S2 in ESI†). Note that there are no K contacts with the CH_2_ group resulting from the zincation reaction. In this case the formation of the benzyl anion and its coordination preference to the bimetallic system allows to maximise the number of K–arene interactions, which could greatly contribute to the overall stability of this higher order zincate, precluding the ligand redistribution processes described above. Previous work by our group has also shown that when KO*t*Bu is added to a solution of Zn(TMP)_2_ in toluene, benzylic zincation is observed at room temperature forming a stable intermediate with a structure similar to that of 5.^16^ Potassium zincate 5 displayed onward reactivity towards Weinreb amide *N*-methoxy-*N*-methylbenzamide to give 2-(3,5-dimethylphenyl)-1-phenylethan-1-one 6a in a 76% yield (Fig. 3c). This reactivity was extended to *m*-xylene forming 2-(*m*-tolyl)phenylethan-1-one 6b in a 71% yield, showing the potential of this bimetallic approach to promote benzylic aroylation of non-activated toluenes using Weinreb amides (see ESI for details†). The conditions employed are significantly milder than those previously reported employing Group 1 metal amides.^40^

**Fig. 6:**
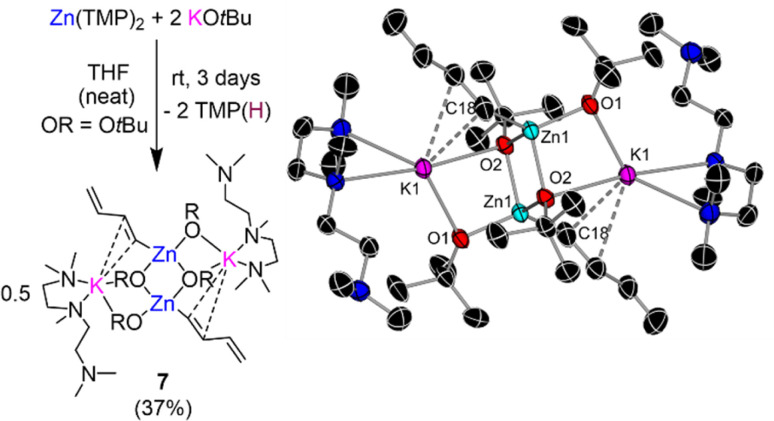
Reaction of Zn(TMP)_2_/2KO*t*Bu with excess THF forming [(PMDETA)KZn(C_4_H_5_)(O*t*Bu)_2_]_2_ (7) and its molecular structure with displacement ellipsoids at 50% probability, all H atoms omitted and with C atoms in the alkoxide fragment drawn as wire frames for clarity.

Curiously, in the absence of any substrate, leaving a solution of Zn(TMP)_2_/2KO*t*Bu in d_8_-THF to stand at room temperature for 3 days was accompanied with a dramatic colour change from colourless to an intense bright purple solution. ^1^H-NMR analysis of this solution indicated that all Zn-TMP signals had converted to TMP(D), indicating a possible deprotonative metalation reaction between the zinc base and the THF solvent. Intrigued by this potential C-sp^3^ metalation, our Zn(TMP)_2_/2KO*t*Bu combination was left to stir in protic THF over the course of three days forming a deep purple/black solution with a black deposit left on the Teflon stirrer bar. A work-up of this reaction in hexane with the addition of Lewis donor PMDETA afforded a colourless crop of crystals revealed by NMR spectroscopy and X-ray crystallography to be the potassium zincate [(PMDETA)KZn(C_4_H_5_)(O*t*Bu)_2_]_2_ (7) (crystalline yield 37% of a maximum 50% yield). The molecular structure of 7 (Fig. 6) closely resembles the dimeric ladder motif described for potassium zincates 3a–c (Fig. 4 and S1†). However, in this case an unexpected *s-trans*-1,3-butadienyl (C_4_H_5_^−^) fragment resides terminally on the Zn centre. Bond lengths within this chain (C18–C19, 1.351(3) Å; C19–C20, 1.467(3) Å; C20–C21, 1.315(3) Å) denote a localized double-bond, single bond, double bond pattern consistent with that in the parent diene.^41^ The butadienyl is connected to Zn through a Zn1–C18 σ-bond [1.999(15) Å] and interacts with the K atom through π-type contacts *via* two of its C atoms, K1–C18 [3.174(15) Å] and K1–C19 [3.372(2) Å]. The K atoms in 7 are then capped by two N donor atoms from PMDETA whilst the third N atom of the Lewis donor lies pendant.

While strong bases such as organolithium reagents are known to metalate THF, typically the reverse [3 + 2] cycloaddition products are formed as a consequence of the decomposition of the fragile α-lithiated intermediate that rapidly breaks down into ethene and the corresponding lithium enolate.^42,43^ However, Mulvey has previously reported a related compound in the form of a di-magnesiated butadiene fragment, [(TMEDA){Na(TMP)}_2_{1,4-(Mg(TMP))_2_-C_4_H_4_}] as a result of the reaction between sodium magnesiate [(TMEDA)NaMg(TMP)_2_R] (R = CH_2_SiMe_3_) and one equivalent of THF in hexane.^44^ Remarkably, in that study the decapitated oxygen heteroatom could be trapped in the form of an oxide inverse crown ether. Mulvey has further demonstrated how bimetallic zincates can tame exceptionally sensitive anions reporting the α-zincation of THF using sodium zincate [(TMEDA)NaZn(TMP)(CH_2_SiMe_3_)_2_], where the metalated product is stable, the heterocyclic motif remains intact, and the tetrahydrofuranyl fragment can be transferred to electrophiles such as benzoyl chloride.^45^ The presence of two equivalents of TMP(H) in our reaction mixture between excess THF and Zn(TMP)_2_/2KO*t*Bu indicate an initial α-metallation of the cyclic ether similar to that reported by Mulvey, though attempts to isolate such an intermediate were unsuccessful.

In an effort to ascertain whether this zincate 7 was indeed the product of an intriguing decomposition of THF, the reaction was carried out in d_8_-THF and subsequent deuterium-NMR analysis indicated deuterium incorporation into the C_4_H_5_ diene fragment displaying broad multiplets, at similar chemical shifts in the ^2^H-NMR (6.76, 6.22, 5.11 and 4.68 ppm, see ESI for details†) to those observed in the ^1^H-NMR spectrum of 7 (6.68, 6.23, 4.73 and 4.54 ppm). Frustratingly attempts to isolate additional side products or other organometallic species in formation of 7 proved futile and thus the mechanism of this decomposition reaction remains unknown. The competing THF metallation, and subsequent decomposition that furnishes 7, poses a problem when longer reaction times are needed for full zincation of non-activated substrates such as benzene or mesitylene where it is necessary for the arene to be used as a solvent (or in excess) to avoid side reactions of Zn(TMP)_2_/2KO*t*Bu with THF.

## Conclusions

In conclusion, this work has revealed that two relatively weak metalating agents, KO*t*Bu and Zn(TMP)_2_, can combine to form a powerful basic mixture in solution capable of performing challenging regioselective zincations of non-activated arenes such as naphthalene, biphenylene and anthracene. A dramatic alkali-metal effect was observed within these reactions, where switching from KO*t*Bu to the lighter alkali-metal congeners Li and NaO*t*Bu completely shuts down the metallation of the arene, highlighting the pivotal role of the alkali-metal in these zincation reactions. X-Ray crystallographic and NMR spectroscopic analysis of the metalated intermediates of these reactions added a new layer of complexity to these systems where mixed aryl/alkoxy higher order potassium zincates can undergo a redistribution process in which potassium aryl species are formed. However, this redistribution process can be easily circumvented by stabilisation of the K atom through π–arene interactions retaining the integrity of the higher order potassium zincates in both solution and solid states. Finally, this study has also captured and characterised an unusual decomposition product of THF, likely through an initial sp^3^-C α-metallation of the common cyclic ether, demonstrating the power of this Zn(TMP)_2_/2KO*t*Bu combination.

## Data availability

Crystallographic data: deposition numbers 2358265 (3a), 2358266 (3b), 2358267 (3c), 2358268 (5), and 2358269 (7) contain the ESI crystallographic data for this paper. Experimental procedures and analytical data (NMR and elemental analysis) can be found in the ESI.† Copies of NMR spectra are also provided.

## Author contributions

Neil R. Judge performed the experimental work. E. Hevia supervised the work. All authors participated in the writing of the manuscript and approved its last version.

## Conflicts of interest

There are no conflicts to declare.

## Supplementary Material

SC-OLF-D4SC03892D-s001

SC-OLF-D4SC03892D-s002
