# Supplementary material for: Combining two relatively weak bases (Zn(TMP)2 and KOtBu) for the regioselective metalation of non-activated arenes and heteroarenes
Source: Chem Sci. 2024 Aug 13;15(36):14757–65. doi: 10.1039/d4sc03892d (PMC11334761; doi:10.1039/d4sc03892d)
Supplement: SC-015-D4SC03892D-s001 [file SC-015-D4SC03892D-s001.pdf]

Supplementary Information (SI) for Chemical Science.
This journal is © The Royal Society of Chemistry 2024

Electronic Supplementary Information

Combining Two Relatively Weak Bases ($Zn(TMP)_2$ and KO*t*Bu) for the Regioselective Metalation of Non-Activated Arenes and Heteroarenes

Neil R. Judge and Eva Hevia*

Departement für Chemie, Biochemie und Pharmacie, Universität Bern, 3012 Bern, Switzerland.

E-mail: eva.hevia@unibe.ch and neil.judge@unibe.ch

Contents

1. General Methods ... 2

2. X-Ray Crystallographic Details ... 3

2.1 Selected crystallographic parameters for compounds ... 4

2.2 X-ray crystal structures of compounds **3b** and **5** ... 6

3. Synthetic procedures ... 8

3.1 Naphthalene iodination optimisation ... 8

3.2 Synthesis of 2-iodonaphthalene **2a** via $Zn(TMP)_2$/2KO*t*Bu ... 9

3.3 Synthesis of iodoarenes **2b-2o** ... 11

3.4 Synthesis of organometallic compounds **3a, 3b, Ib, 3c**, **4, 5, and 7** ... 30

3.5 Synthesis of acetophenones **6a** and **6b** ... 43

4. NMR Reaction Monitoring ... 46

4.1 Zincation and iodination of Naphthalene using $Zn(TMP)_2$/2KO*t*Bu ... 46

4.2 Zincation and iodination of anthracene and possible competing SET process ... 49

4.3 *in-situ* zincation of 1,3-benzoxazole and generation of **3c** and **4** ... 52

4.4 Zincation of 1,3-benzothiazole and in-situ generation of **Id** ... 54

4.5 THF metalation and decomposition with $Zn(TMP)_2$/2KO*t*Bu ... 58

5. References ... 62

1. General Methods

All procedures were conducted using standard Schlenk and glove box techniques under an inert atmosphere of argon.[1] Solvents were degassed, purified and collected via MBraun SPS 5 and stored over 4 Å molecular sieves for at least 24 hours prior to use. THF was dried by heating to reflux over sodium-wire/benzophenone ketyl radical and stored over 4 Å molecular sieves for 24 hours prior to use. Deuterated benzene (C_6D_6) and THF (D_8-THF) were purchased from Euroisotop and/or Merck (Sigma Aldrich), dried over NaK alloy for 16 hours and then cycled through three rounds of degassing by employing a freeze-pump-thaw method. The deuterated solvents were then collected via a vacuum transfer method and stored under inert atmosphere over 4 Å molecular sieves. $Zn(TMP)_2$ was synthesised according to literature procedures.[2] All other reagents purchased from commercial suppliers and used as received.

Optimal results for iodine quenches obtained with sublimed iodine (I_2) (purchased from Thermo Scientific – 99.5% extra pure, resublimed). Once opened, iodine kept in a desiccator under argon to prevent any H_2O contamination.

NMR spectra were recorded on Bruker spectrometers operating at either 300 or 400 MHz. ^{1}H NMR spectra: 300.1 or 400.1 MHz, ^{7}Li NMR spectra: 116.6 or 155.5 MHz, ^{13}C{^{1}H} NMR spectra: 75.5 or 100.6 MHz.

Elemental analyses (C, H and N) were conducted with a Flash 2000 Organic Elemental Analyser (Thermo Scientific). Samples were prepared in the glovebox under argon atmosphere and sealed in an air-tight container prior to analyses. All results were obtained in triplicate to ensure consistency.

Purification of the final organic products were performed by column chromatography on silica gel using a CombiFlash®Rf system (Teledyne ISCO), with RediSep® Silver Normal-phase Silica Flash Columns as stationary phase and mixtures of hexane and ethyl acetate as mobile phase.

2. X-Ray Crystallographic Details

Single crystal X-ray diffraction (Universität Bern) measurements were made on a *RIGAKU Synergy S* area-detector diffractometer using mirror optics monochromated Cu *K*α radiation (λ = 1.54184 Å). Data reduction was performed using the *CrysAlisPro* program. The intensities were corrected for Lorentz and polarization effects, and an absorption correction based on the multi-scan method using SCALE3 ABSPACK in *CrysAlisPro* was applied. The structures were solved by direct methods using *SHELXT*.[3]

Refinement of the structure was carried out on F^2 using full-matrix least-squares procedures, which minimized the function $\Sigma w(F_o^2 - F_c^2)^2$. The weighting scheme was based on counting statistics and included a factor to downweight the intense reflections. All calculations were performed using the *SHELXL-2014/7* [4] program in OLEX2 [5] which revealed the positions of all non-hydrogen atoms of the title compound. All non-hydrogen atoms were refined anisotropically. H-atoms were assigned in geometrically calculated positions and refined using a riding model where each H-atom was assigned a fixed isotropic displacement parameter with a value equal to 1.2Ueq of its parent atom (1.5 Ueq for methyl groups).

2.1 Selected crystallographic parameters for compounds

Table S 1 Selected crystallographic parameters for compounds.

	**3a**	**3b**	**3c**	**5**	**7**
CCDC Number	2358265	2358266	2358267	2358268	2358269
Empirical formula	$C_{52}H_{82}K_2O_8Zn_2$	$C_{44}H_{78}K_2O_8Zn_2$	$C_{46}H_{76}K_2N_2O_{10}Zn_2$	$C_{34}H_{56}K_2O_4Zn$	$C_{42}H_{92}K_2N_6O_4Zn_2$
Mol. Mass	1044.11	944	1026.02	672.35	954.15
Temperature/K	173.01(10)	173.00(10)	100.0(2)	173.00(10)	173.01(10)
Crystal system	monoclinic	monoclinic	orthorhombic	monoclinic	triclinic
Space group	I2/a	$P2_1/n$	Pbca	$P2_1/c$	P-1
a/Å	18.2697(3)	12.74441(7)	18.3733(3)	15.08965(17)	10.92429(16)
b/Å	17.0018(3)	14.35189(9)	15.7455(3)	26.6881(3)	11.04872(16)
c/Å	20.8902(3)	14.07101(7)	18.5165(3)	18.63986(19)	13.15156(19)
α/°	90	90	90	90	112.4692(14)
β/°	112.7772(18)	94.0280(5)	90	102.2607(10)	92.8879(12)
γ/°	90	90	90	90	107.7984(13)
Volume/$Å^3$	5982.84(18)	2567.32(2)	5356.74(16)	7335.31(14)	1371.22(4)
Z	4	2	4	8	1
$\rho_{calc}g/cm^3$	1.159	1.221	1.272	1.218	1.155
μ/mm^{-1}	2.577	2.946	2.909	0.929	2.733
F(000)	2224	1008	2176	2880.0	516
Crystal size/mm^3	0.131 × 0.069 × 0.067	0.317 × 0.266 × 0.186	0.252 × 0.234 × 0.197	0.257 × 0.226 × 0.156	0.236 × 0.183 × 0.122

Radiation	Cu Kα (λ = 1.54184)	Cu Kα (λ = 1.54184)	Cu Kα (λ = 1.54184)	Mo Kα (λ = 0.71073)	Cu Kα (λ = 1.54184)
2Θ range for data collection/°	6.934 to 142.53	8.812 to 140.918	8.804 to 149.008	4.116 to 61.016	7.41 to 135.808
Index ranges	-22 ≤ h ≤ 22, -20 ≤ k ≤ 20, -25 ≤ l ≤ 21	-15 ≤ h ≤ 15, -17 ≤ k ≤ 17, -16 ≤ l ≤ 17	-17 ≤ h ≤ 22, -19 ≤ k ≤ 19, -23 ≤ l ≤ 23	-21 ≤ h ≤ 21, -38 ≤ k ≤ 38, -26 ≤ l ≤ 26	-13 ≤ h ≤ 13, -13 ≤ k ≤ 13, -15 ≤ l ≤ 15
Reflections collected	59420	50337	50695	222585	51627
Independent reflections	5794 [R_{int} = 0.0602, R_{sigma} = 0.0256]	4926 [R_{int} = 0.0323, R_{sigma} = 0.0134]	5474 [R_{int} = 0.0375, R_{sigma} = 0.0176]	22397 [R_{int} = 0.0432, R_{sigma} = 0.0258]	4979 [R_{int} = 0.0224, R_{sigma} = 0.0086]
Data/restraints/parameters	5794/115/295	4926/18/315	5474/48/306	22397/104/848	4979/5/443
Goodness-of-fit on F^2	1.062	1.049	1.077	1.015	1.074
Final R indexes [I>=2σ (I)]	R_1 = 0.0625, wR_2 = 0.1977	R_1 = 0.0286, wR_2 = 0.0784	R_1 = 0.0473, wR_2 = 0.1317	R_1 = 0.0475, wR_2 = 0.1172	R_1 = 0.0213, wR_2 = 0.0562
Final R indexes [all data]	R_1 = 0.0691, wR_2 = 0.2066	R_1 = 0.0290, wR_2 = 0.0788	R_1 = 0.0525, wR_2 = 0.1367	R_1 = 0.0691, wR_2 = 0.1269	R_1 = 0.0215, wR_2 = 0.0564
Largest diff. peak/hole / e $Å^{-3}$	0.75/-0.48	0.32/-0.26	0.52/-0.65	0.62/-0.47	0.38/-0.38

2.2 X-ray crystal structures of compounds **3b** and **5**

[(THF)$_2$KZn(2-C$_{10}$H$_7$)(O*t*Bu)$_2$]$_2$ (**3b**)

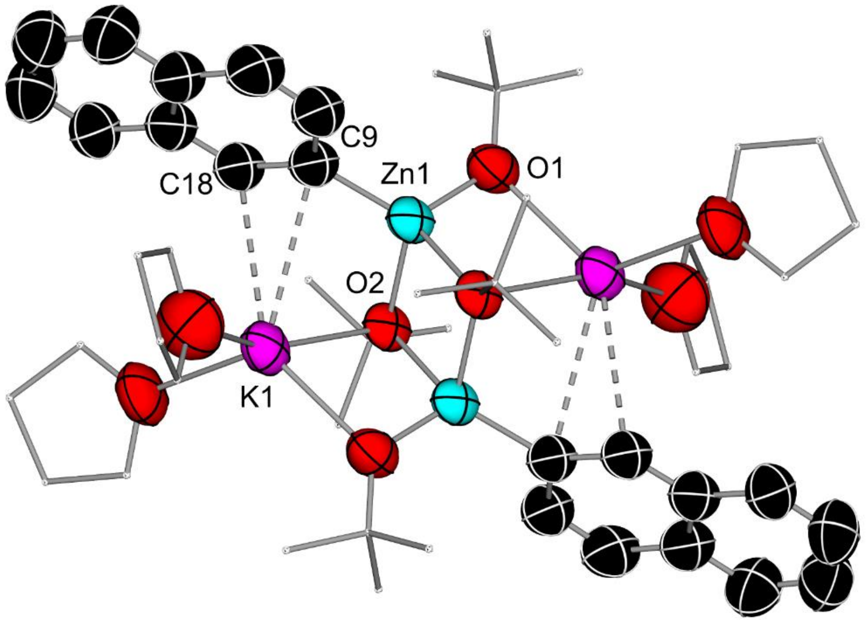

Figure S 1 Molecular structure of [(THF)$_2$KZn(2-C$_{10}$H$_7$)(O*t*Bu)$_2$]$_2$ (3b) with displacement ellipsoids at 50% probability, all H atoms omitted and with C atoms in the alkoxide substituent and THF molecules drawn as wire frames for clarity.

[(THF)$_2$K$_2$Zn(CH$_2$-3,5-Me$_2$-C$_6$H$_3$)$_2$(O*t*Bu)$_2$]$_\infty$ (**5**) – polymeric fragment

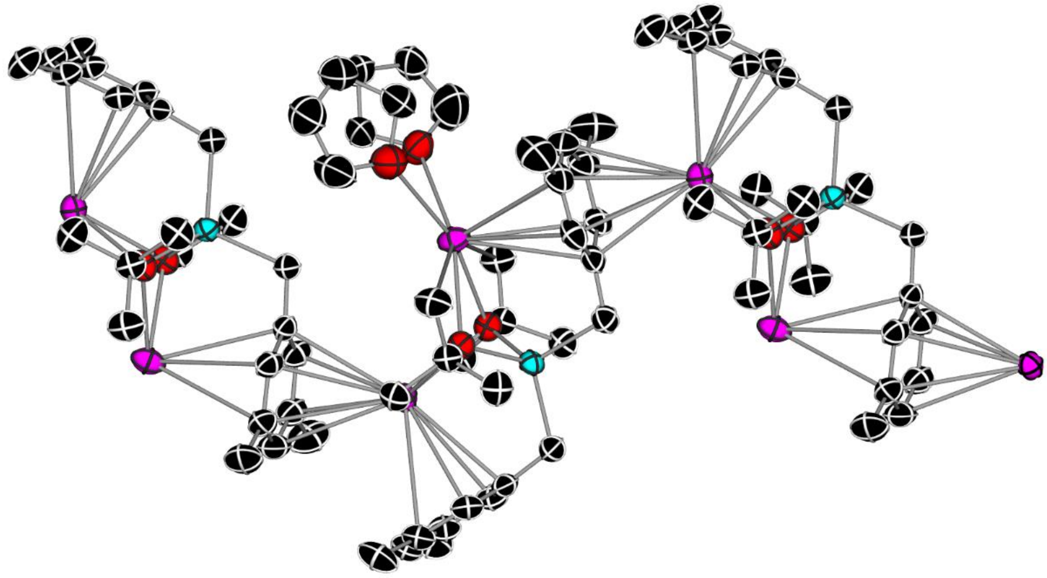

Figure S 2 2D polymeric fragment of [(THF)$_2$K$_2$Zn(CH$_2$-3,5-Me$_2$-C$_6$H$_3$)$_2$(O*t*Bu)$_2$]$_\infty$ (**5**) with displacement ellipsoids at 50% probability, all H atoms omitted for clarity. Structure propagated by K$\cdots\pi$ interactions between the alkali metal in the monomeric unit of **5** and a mesityl moiety in a neighboring unit of **5**.

3. Synthetic procedures

3.1 Naphthalene iodination optimisation

General Procedure

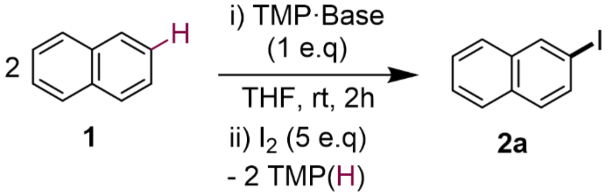

Scheme S 1 Screening of naphthalene (**1**) C2-zincation by different $Zn(TMP)_2$/*n*AMO*t*Bu combinations and subsequent iodine quench forming 2-iodonaphthalene (**2a**).

In an argon flushed Schlenk flask, naphthalene (0.5 mmol, 64 mg) was dissolved in THF (5 mL). To this solution was added the stated base, from Table S2, and left to stir for 2h at room temperature. After the stated time, five equivalents of sublimed I_2 (2.5 mmol, 650 mg) was added and allowed to stir overnight. Reaction mixture quenched with $Na_2S_2O_3$ (10 mL), organics extracted with EtOAc (3 x 10 mL), washed with brine (1 x 10 mL), dried over $MgSO_4$ and filtered. Aliquot taken, all solvent removed, and NMR analysis carried out in $CDCl_3$ using hexamethylbenzene as an internal standard to determine the yield of 2-iodonaphthalene **2a**.

Table S 2 Screening of naphthalene (**1**) C2-zincation by different $Zn(TMP)_2$/2(AM)O*t*Bu (AM = Li, Na, K) pairings and subsequent iodine quench forming 2-iodonaphthalene (**2a**)

Entry	Base	Yield[a] (%)
1	$Zn(TMP)_2$	0[b]
**2**	**$Zn(TMP)_2$ + KO*t*Bu**	**99**
**3**	**$Zn(TMP)_2$ + 2KO*t*Bu**	**99 (89)**[c]
4	$Zn(TMP)_2$ + 2 LiO*t*Bu	0
5	$Zn(TMP)_2$ + 2 NaO*t*Bu	0
6	$Zn(TMP)_2$ + 2 KO*t*Bu + 2 (18-Crown-6)	0
7	KTMP	0[d]
8	LiTMP + KO*t*Bu	<5%[d]
9	LiTMP + $Zn(TMP)_2$	<5%
10	$Zn(TMP)_2$·$2MgCl_2$·2LiCl	<5%

[a] Conversions determined by ^1H NMR monitoring using hexamethylbenzene as internal standard; [b] Reaction refluxed for 2h; [c] isolated yield after purification by column chromatography; [d] 1 equivalent of naphthalene used

3.2 Synthesis of 2-iodonaphthalene **2a** via $Zn(TMP)_2$/2KO*t*Bu

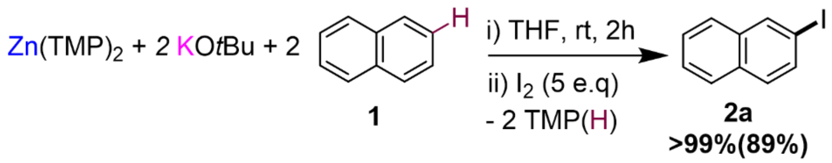

Scheme S 2. Zincation and subsequent iodination of naphthalene via $Zn(TMP)_2$/2KO*t*Bu forming 2-iodonaphthalene **2a**.

In an argon flushed Schlenk flask, naphthalene (2 mmol, 260 mg), $Zn(TMP)_2$ (1 mmol, 350 mg) and KO*t*Bu (2 mmol, 220 mg) were dissolved in THF (5 mL). After 2h at room temperature, I_2 (5 mmol, 1.2 g or 5 mL of a 1M solution in THF) was added and stirred overnight. Reaction mixture quenched with $Na_2S_2O_3$ (10 mL), organics extracted with EtOAc (3 x 10 mL), washed with brine (1 x 10 mL), dried over $MgSO_4$ and filtered. The mixture concentrated and purified by filtration through a plug of silica gel using hexane as eluent to yield 2-iodonaphthalene **2a** (450 mg, 89%) as an off-white solid. Spectroscopic data are an identical match for those previously reported.[6]

Note: Identical yields are achieved when using just 1 equivalent of KO*t*Bu.

^{1}H-NMR (300.1 MHz, $CDCl_3$, 298 K): δ / ppm = 8.24 (s, 1H), 7.81-7.78 (m, 1H), 7.74-7.71 (m, 2H), 7.58 (d,J= 8.5 Hz, 1H), 7.51-7.49 (m, 2H) ppm.

^{13}C{^{1}H}-NMR (101 MHz, $CDCl_3$, 298 K): δ / ppm = 136.7, 135.1, 134.5, 132.2, 129.6, 128.0, 126.9, 126.8, 126.6, 91.6 ppm.

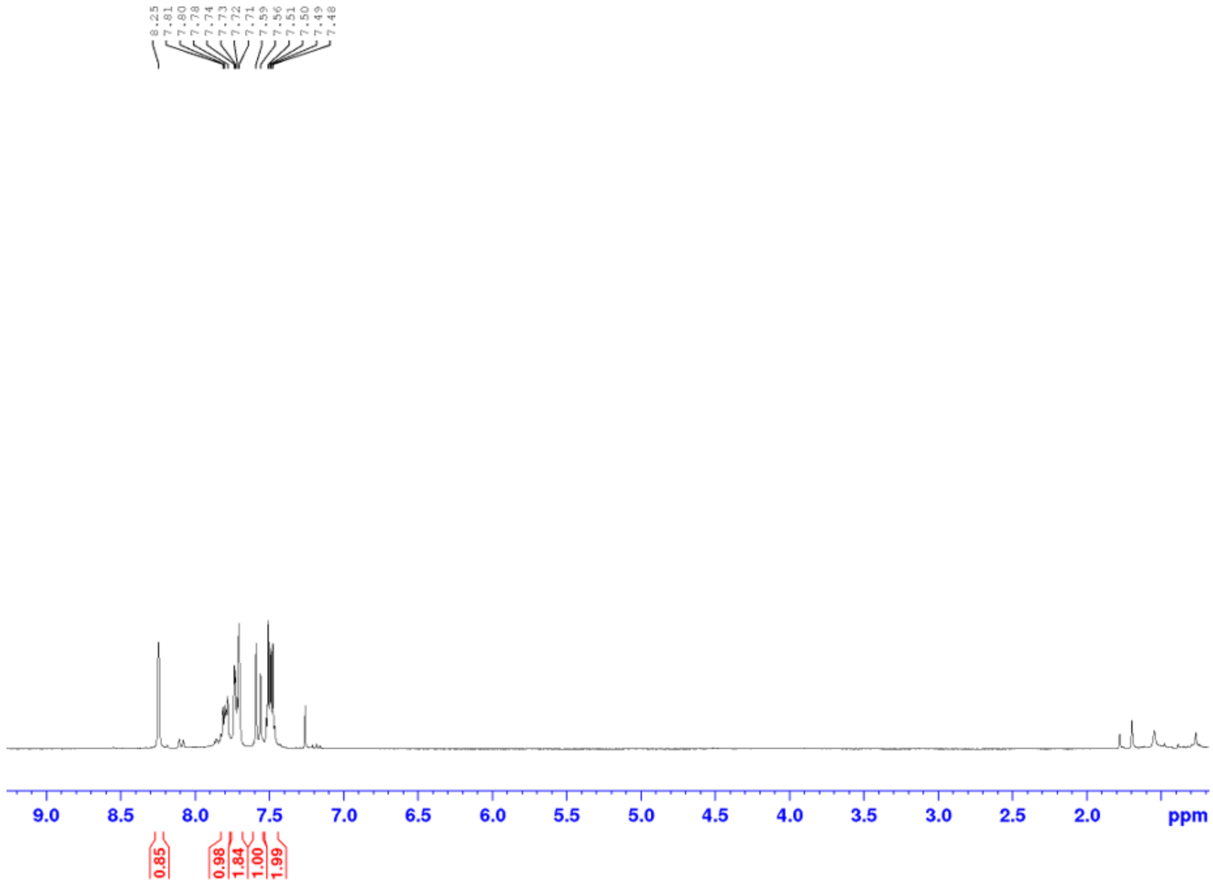

Figure S 3. ^{1}H NMR spectra of 2-iodonaphthalene **2a** in $CDCl_3$.

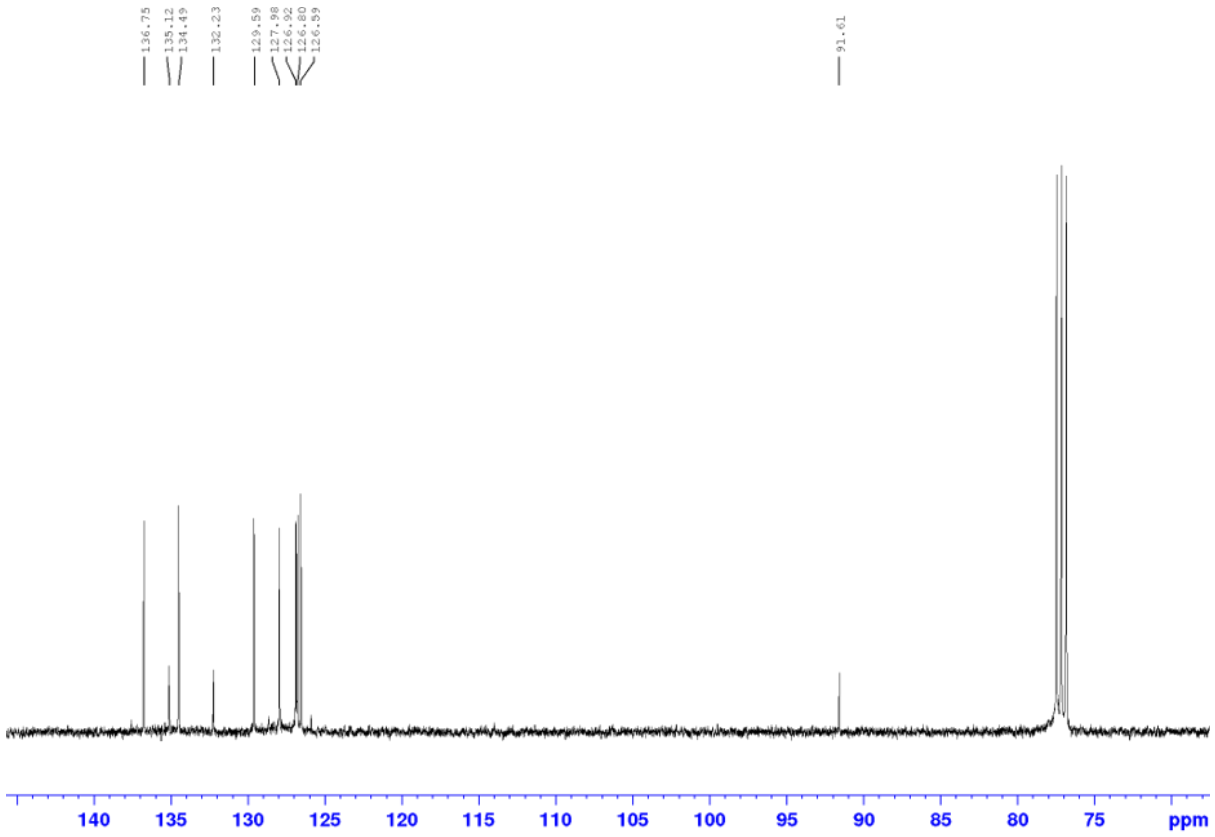

Figure S 4. $^{13}C\{^1H\}$ NMR spectra of 2-iodonaphthalene **2a** in $CDCl_3$.

<u>3.3 Synthesis of iodoarenes **2b-2o**</u>

<u>General Procedure</u>

In an argon flushed Schlenk flask, $Zn(TMP)_2$ (1 e.q) and KO*t*Bu (2 e.q) were dissolved in THF (5 mL). To this solution was added the desired substrate (2 e.q) at room temperature and allowed to stir for the allotted time. Sublimed I_2 (5 e.q) was then added to the solution and then stirred overnight. Reaction mixture quenched with $Na_2S_2O_3$ (10 mL) to remove excess iodine, organics extracted with EtOAc (3 x 10 mL), washed with brine (1 x 10 mL), dried over $MgSO_4$ and filtered. Crude reaction mixture then purified by column chromatography to obtain isolated yields of the relevant iodinated compounds or in the case of **2b** and **2g** yields were determined using hexamethylbenzene as an internal standard (see below).

<u>Synthesis of iodobenzene **2b**</u>

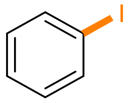

Higher order potassium zincate $K_2Zn(Ph)_2(OtBu)_2$ was prepared as described . A portion of $K_2Zn(Ph)_2(OtBu)_2$ (16 mg, 0.04 mmol) and hexamethylbenzene (10 mg, 15 mol%) were dissolved in d_8-THF and to this solution was added I_2 (5 e.q, 0.15 mmol, 40 mg) giving a deep black solution. After 2 hours at room temperature, ^1H-NMR spectroscopy confirmed a quantitative (>95%) conversion to iodobenzene **2b**. **^1H-NMR (300.1 MHz, d_8-THF, 298 K):** δ / ppm = 7.68 (d, J = 7.85, 2H, Ar-H), 7.32 (m, 1H, Ar-H), 7.10 (t, J = 8.35, 2H, Ar-H). NMR spectroscopic data consistent with literature reports.[7]

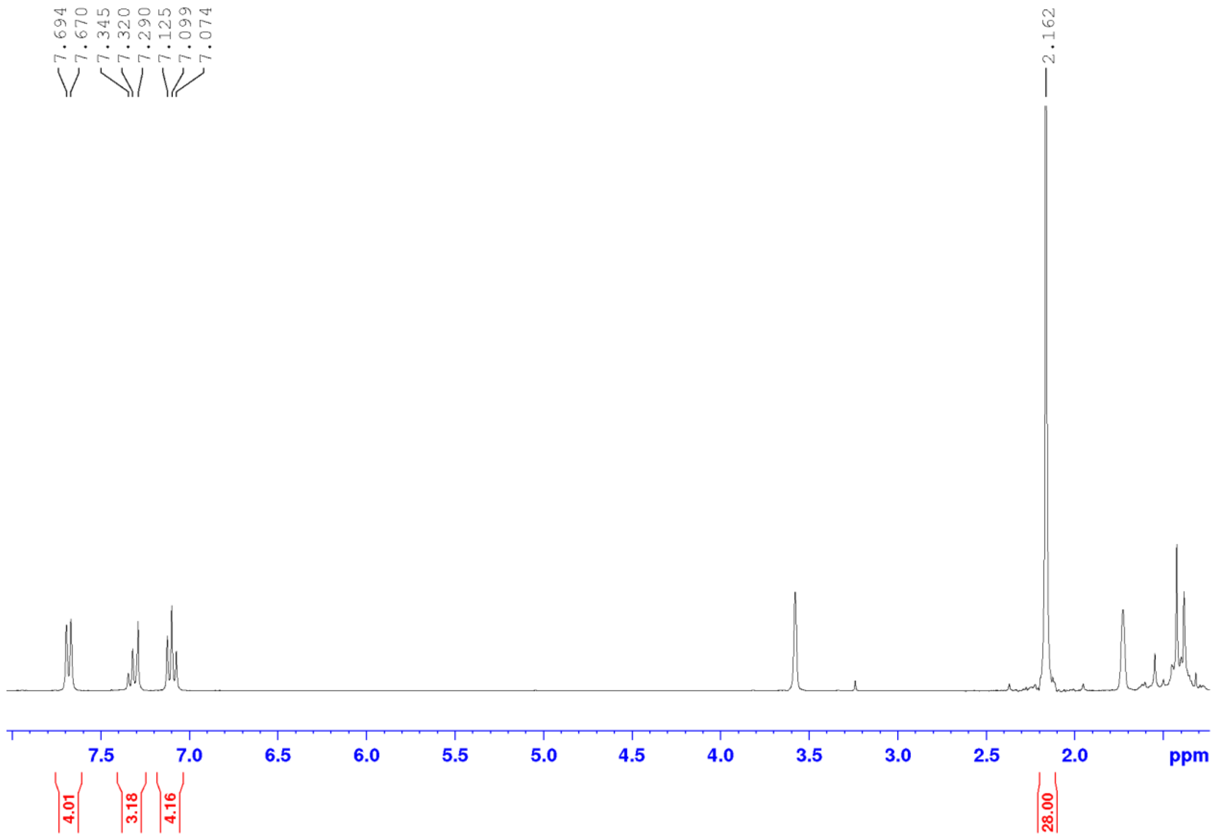

Figure S 5 ^{1}H NMR spectra of iodobenzene **2b** in D_8-THF.

<u>Synthesis of 2-iodoanthracene **2c**</u>

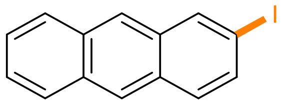

2c was synthesised according to the general procedure using anthracene (2 e.q, 0.5 mmol, 90 mg) stirring the reaction at room temperature for 2 hours giving a deep orange solution. Reaction then quenched with iodine and left to stir overnight. Crude reaction mixture purified by Kugelrohr distillation to give 2-iodoanthracene **2c** as a white crystalline solid (62 mg, 42% yield). NMR spectroscopic data consistent with literature reports.[8] **^{1}H-NMR (300.1 MHz, $CDCl_3$, 298 K):** δ / ppm = 8.42 (s, 1H, Ar-H), 8.38 (s, 1H, Ar-H), 8.29 (s, 1H, Ar-H), 8.01-7.97 (m, 2H, Ar-H), 7.74 (d, 1H, J = 9Hz, Ar-H), 7.65 (dd, 1H, J = 9 Hz, 2 Hz, Ar-H), 7.49 (m, 2H, Ar-H). **^{13}C{^{1}H}-NMR (101 MHz, $CDCl_3$, 298 K):** δ / ppm = 137.0 (s, C_{Ar}-H), 133.8 (s, C_{Ar}-H), 133.1 (s, C_q), 132.1 (s, C_q), 132.0 (s, C_q), 130.1 (s, C_q), 129.8 (s, C_{Ar}-H), 128.4 (s, C_{Ar}-H), 128.3 (s, C_{Ar}-H), 126.7 (s, C_{Ar}-H), 126.1 (s, C_{Ar}-H), 126.0 (s, C_{Ar}-H), 125.3 (s, C_{Ar}-H), 91.3 (s, C_q-I).

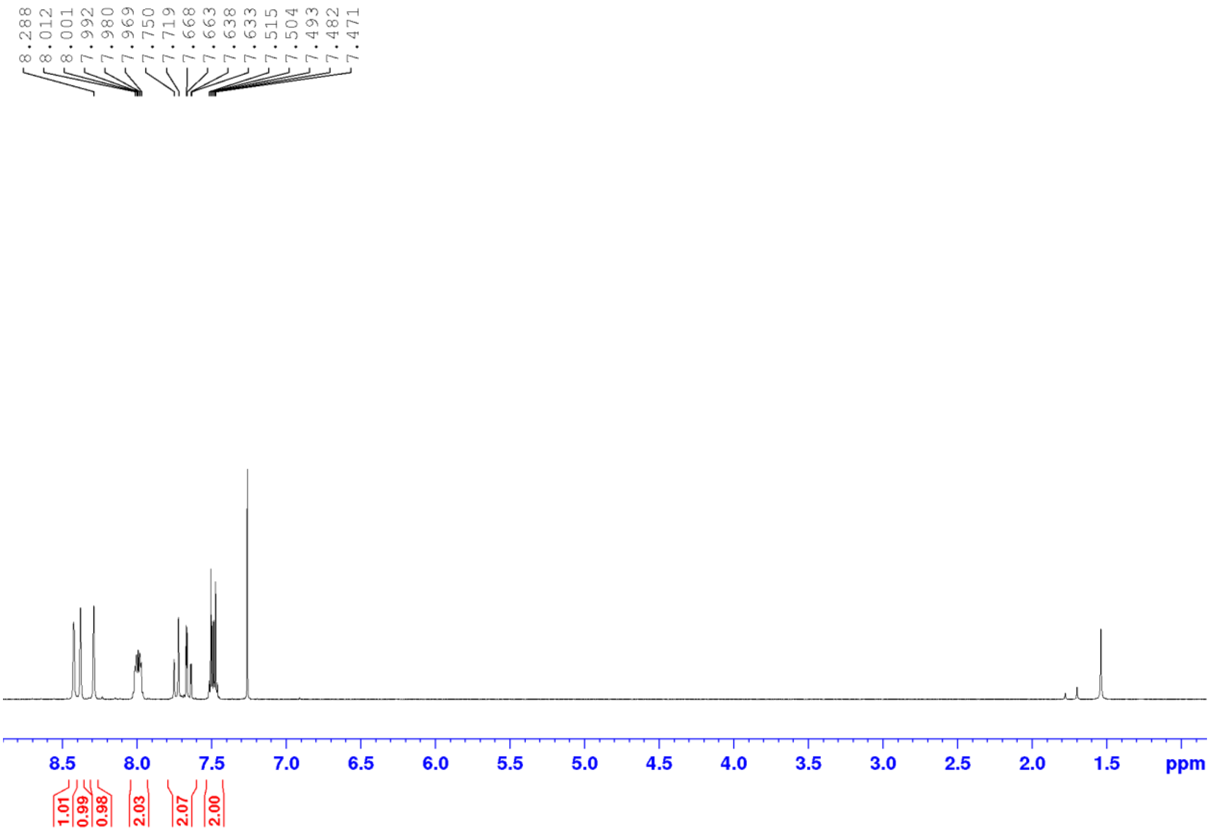

Figure S 6 ^{1}H NMR spectra of 2-iodoanthracene **2c** in $CDCl_3$.

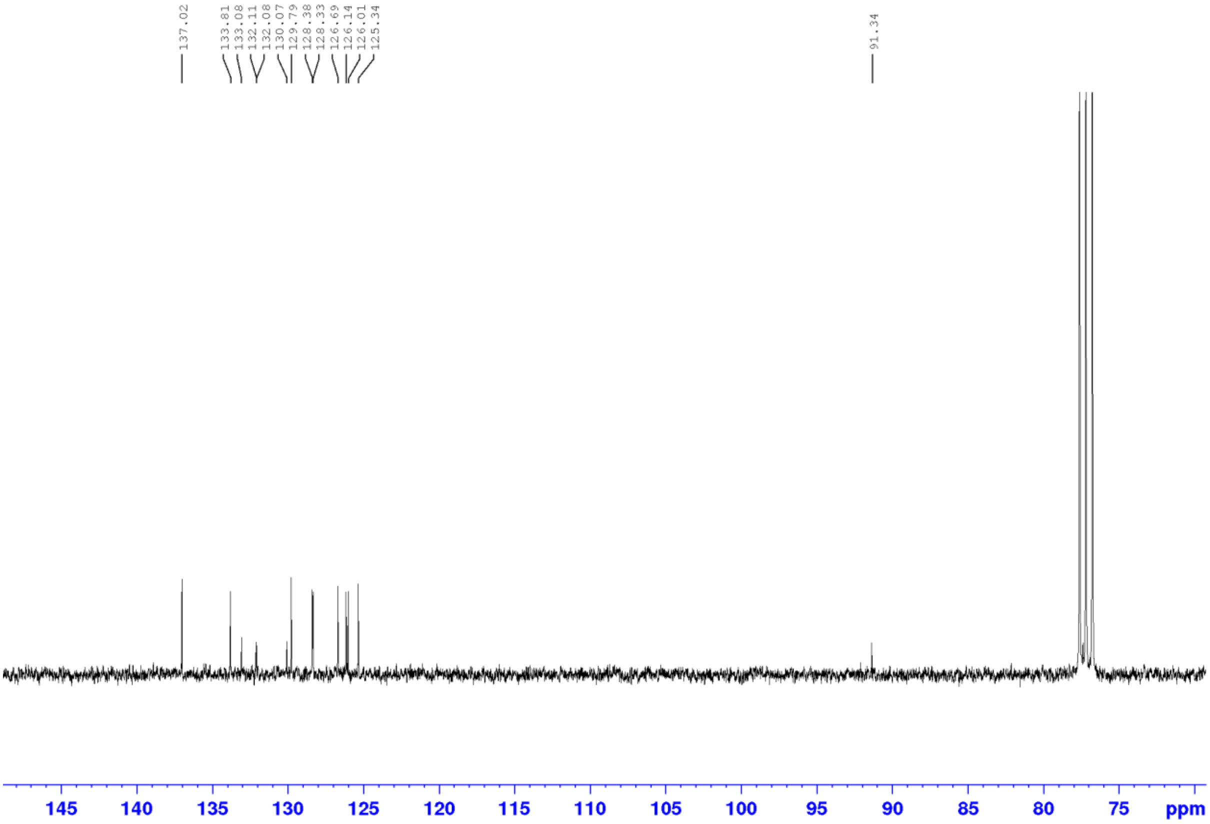

Figure S 7 ^{13}C{^{1}H} NMR spectra of 2-iodoanthracene **2c** in $CDCl_3$.

Synthesis of 1-iodobiphenylene **2d**

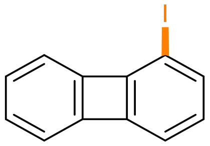

2d was synthesised according to the general procedure using biphenylene (2 e.q, 0.5 mmol, 76 mg) stirring the reaction at room temperature for 24 hours giving a pale orange solution before quenching with iodine. The resulting mixture was concentrated and purified by filtration by column chromatography using hexane as eluent to yield 1-iodobiphenylene **2d** (100 mg, 71%) as a yellow oil. **^{1}H-NMR (300.1 MHz, $CDCl_3$, 298 K):** δ / ppm = 6.95 (d, J = 8.32 Hz, 1H), 6.82 (m, 3H), 6.65 (m, 1H), 6.56 (d, J = 7.06, 1H), 6.46 (m, 1H). **^{13}C{^{1}H}-NMR (101 MHz, $CDCl_3$, 298 K):** δ / ppm = 156.8 (s, C_q, Ar), 152.9 (s, C_q, Ar), 151.2 (s, C_q, Ar), 149.7 (s, C_q, Ar), 136.3 (s, C_q, C-H), 129.8 (s, Ar, C-H), 129.3 (s, Ar, C-H), 128.7 (s, Ar, C-H), 117.9 (s, Ar, C-H), 116.6 (s, Ar, C-H), 80.8 (s, Ar, C_q-I). **HRMS (EI, 70 eV)** m/z: calc. for **$C_{12}H_7I$** 277.9587; found: 277.9590.

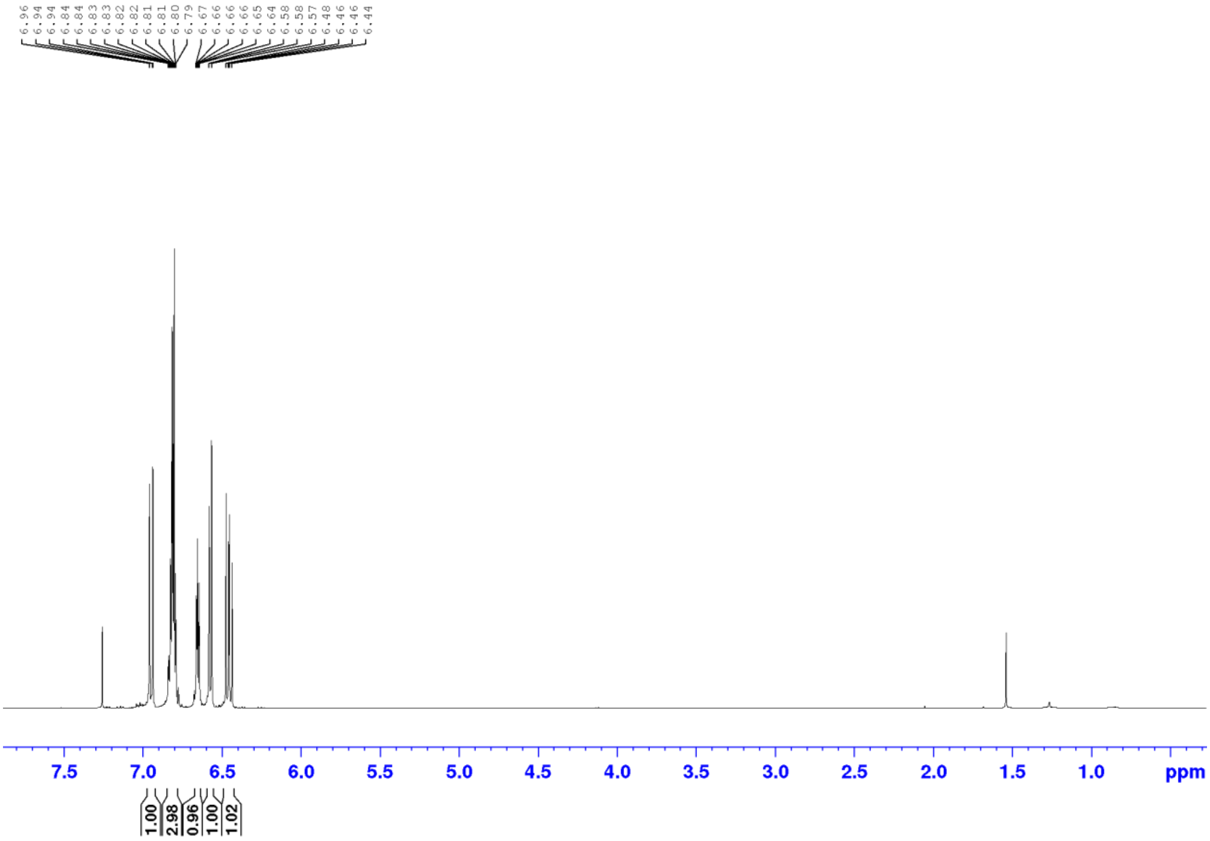

Figure S 8 ^{1}H NMR spectra of 1-iodobiphenylene **2d** in $CDCl_3$.

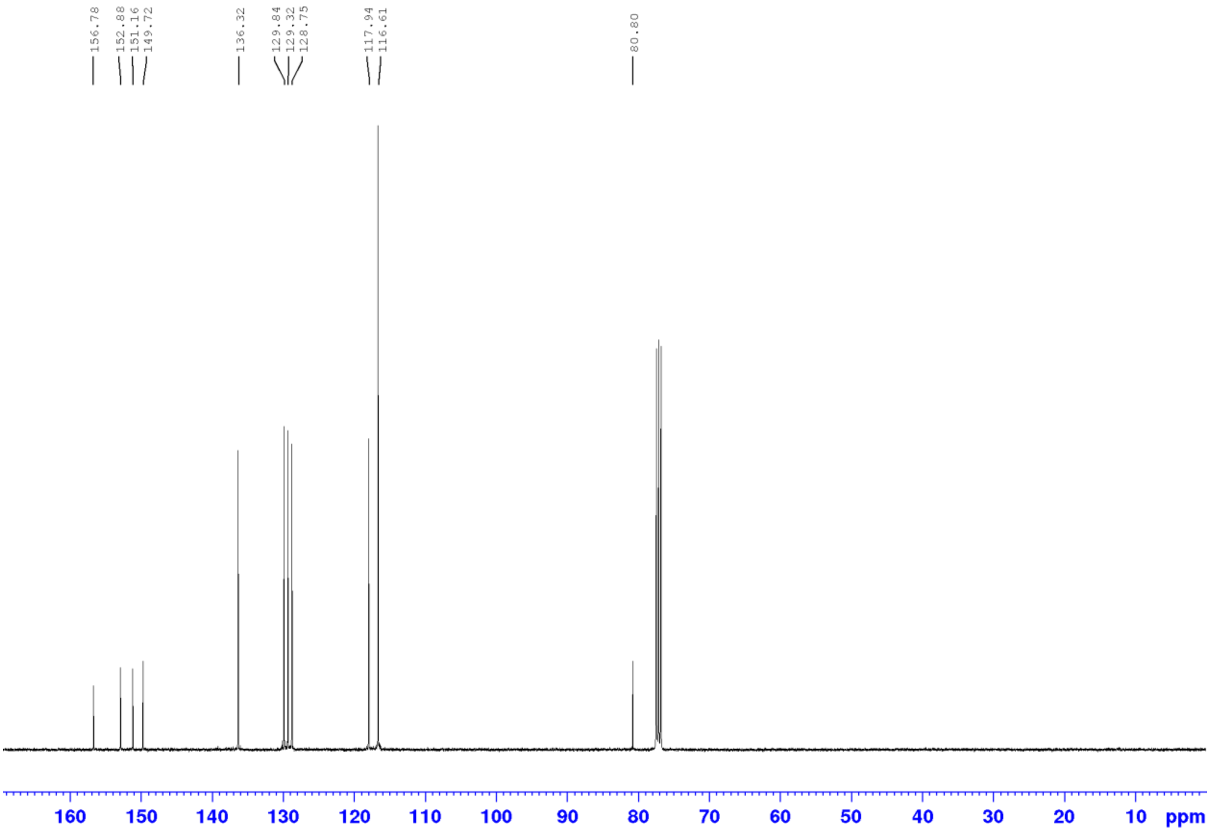

Figure S 9 $^{13}C\{^1H\}$ NMR spectra of 1-iodobiphenylene **2d** in $CDCl_3$.

<u>Synthesis of 3-iodo-2-methoxynaphthalene **2e**</u>

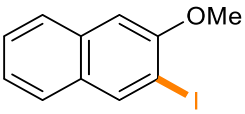

2e was synthesised according to the general procedure using 2-methoxynaphthalene (2 e.q, 79 mg, 0.5 mmol) stirring the reaction at room temperature for 3 hours giving a pale-yellow solution before quenching with iodine. Crude reaction mixture purified by column chromatography (distilled pentane, using fine silica gel, 0.015-0.04 mm) affording of 3-iodo-2-methoxynaphthalene **2e** as a white solid (175 mg, 62%). NMR spectroscopic data consistent with literature reports.[9] **^{1}H-NMR (300.1 MHz, $CDCl_3$, 298 K):** δ / ppm = 8.33 (s, 1H, Ar-H), 7.73-7.66 (m, 1H, Ar-H), 7.64 (m, 1H, Ar-H), 7.50 (t, J = 7.37 Hz, 1H, Ar-H), 7.35 (t, J = 7.62, 1H, Ar-H), 3.99 (s, 3H, OMe). **$^{13}C\{^1H\}$-NMR (101 MHz, $CDCl_3$, 298 K):** δ / ppm = 155.2 (s, C_q), 139.3 (s, C_{Ar}-H), 134.4 (s, C_q), 130.5 (s, C_q), 127.0 (s, C_{Ar}-H), 126.7 (s, C_{Ar}-H), 126.6 (s, C_{Ar}-H), 124.4 (s, C_{Ar}-H), 105.6 (s, C_{Ar}-H), 88.2 (s, Ar, C_q-I), 55.5 (s, OMe_3).

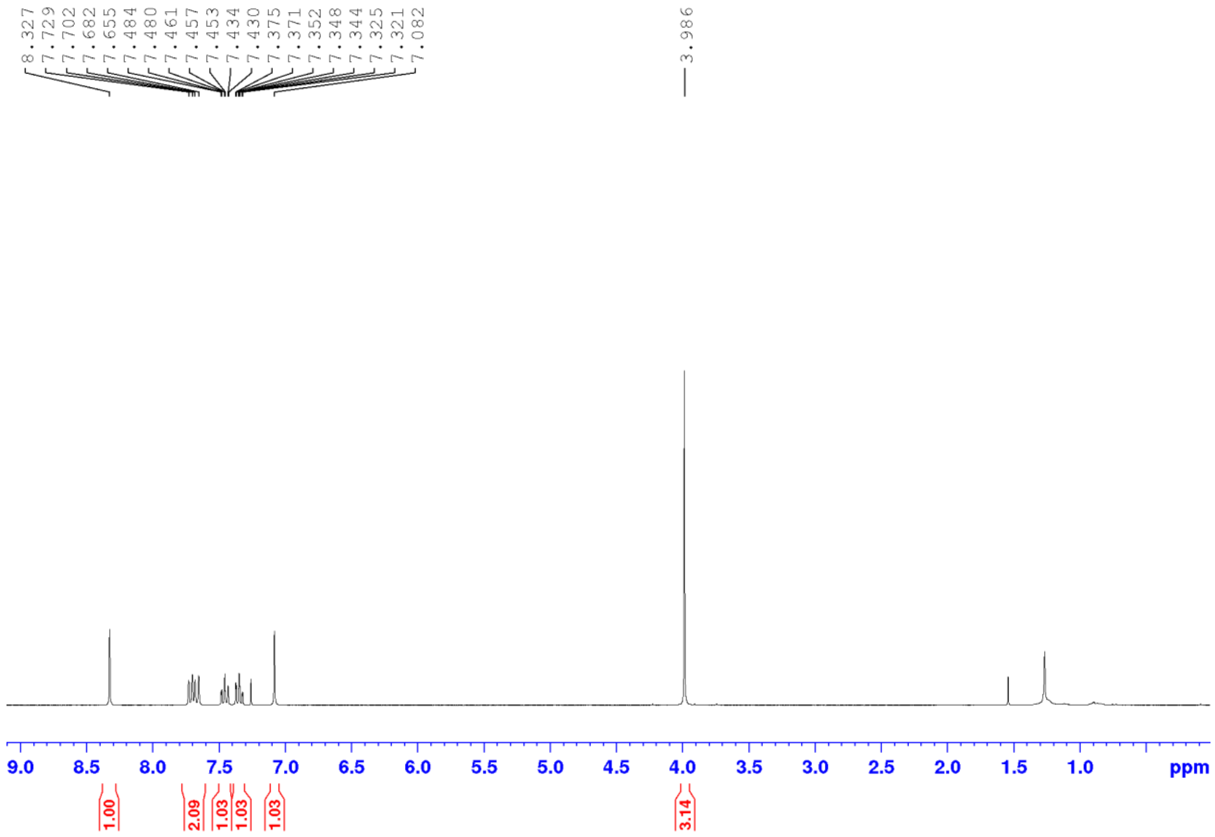

Figure S 10 ^{1}H NMR spectra of 3-iodo-2-methoxynaphthalene **2e** in $CDCl_3$.

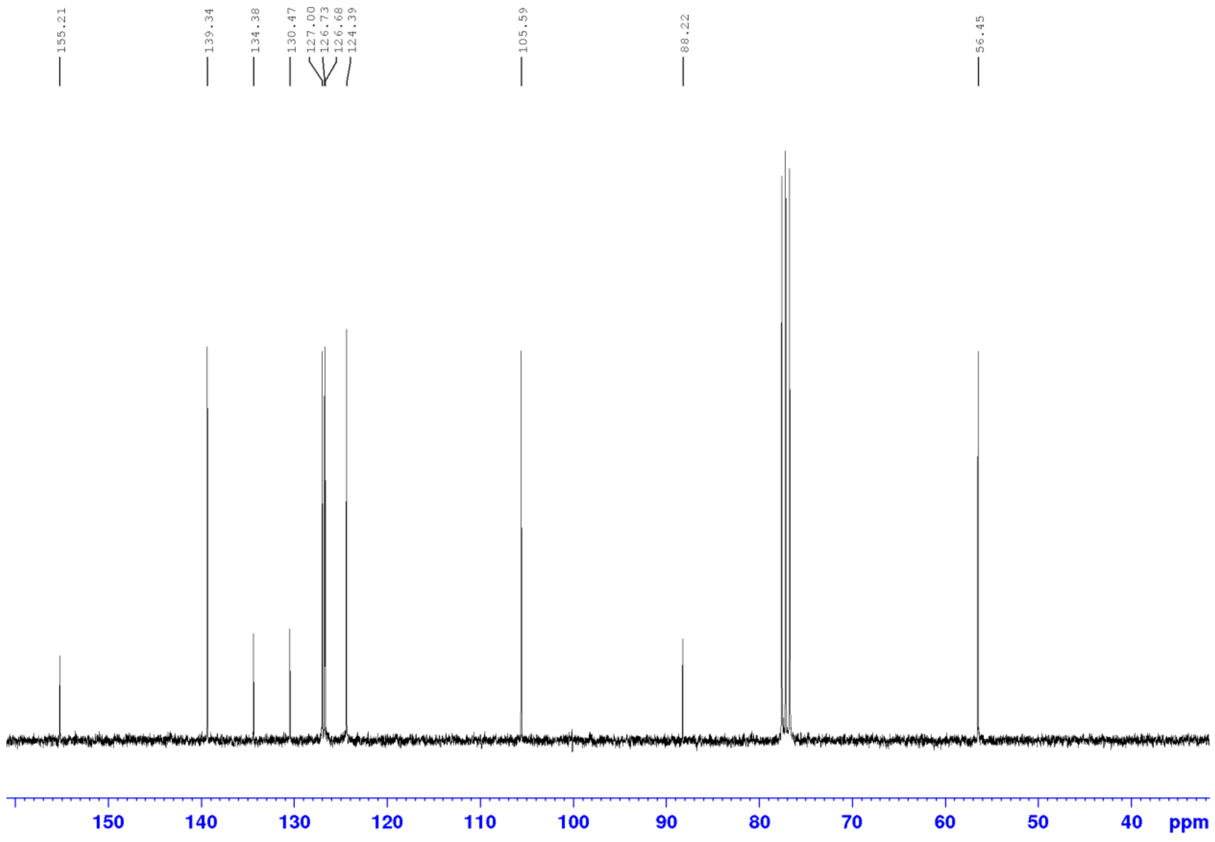

Figure S 11 $^{13}C\{^{1}H\}$ NMR spectra of 3-iodo-2-methoxynaphthalene **2e** in $CDCl_3$.

Synthesis of 1-iodoferrocene **2f**

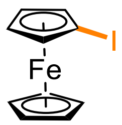

2f was synthesised according to the general procedure using ferrocene (2 e.q, 93 mg, 0.5 mmol) stirring the reaction at room temperature for 24 hours giving a bright orange solution before quenching with iodine. Crude reaction mixture purified by column chromatography (hexane 100%) affording 1-iodoferrocene **2f** as an orange oil (235 mg, 75%). NMR spectroscopic data consistent with literature reports.[10] Trace amounts of ferrocene unable to be separated by column chromatography present in ^{1}H and ^{13}C NMR spectra. **^{1}H-NMR (300.1 MHz, CDCl$_3$, 298 K):** δ / ppm = 4.41 (t, J = 1.80 Hz, 2H, C_5H_4, βH's), 4,18 (s, 5H, C_5H_5), 4.15 (m, 2H, C_5H_4, γH's). **^{13}C{^{1}H}-NMR (101 MHz, CDCl$_3$, 298 K):** δ / ppm = 74.6 (s, 2 x $C_{Cp\text{-}I}$), 71.2 (s, 5 x C_{cp}), 68.9 (s, 2 x $C_{Cp\text{-}I}$), 39.9 (s, C_q-I).

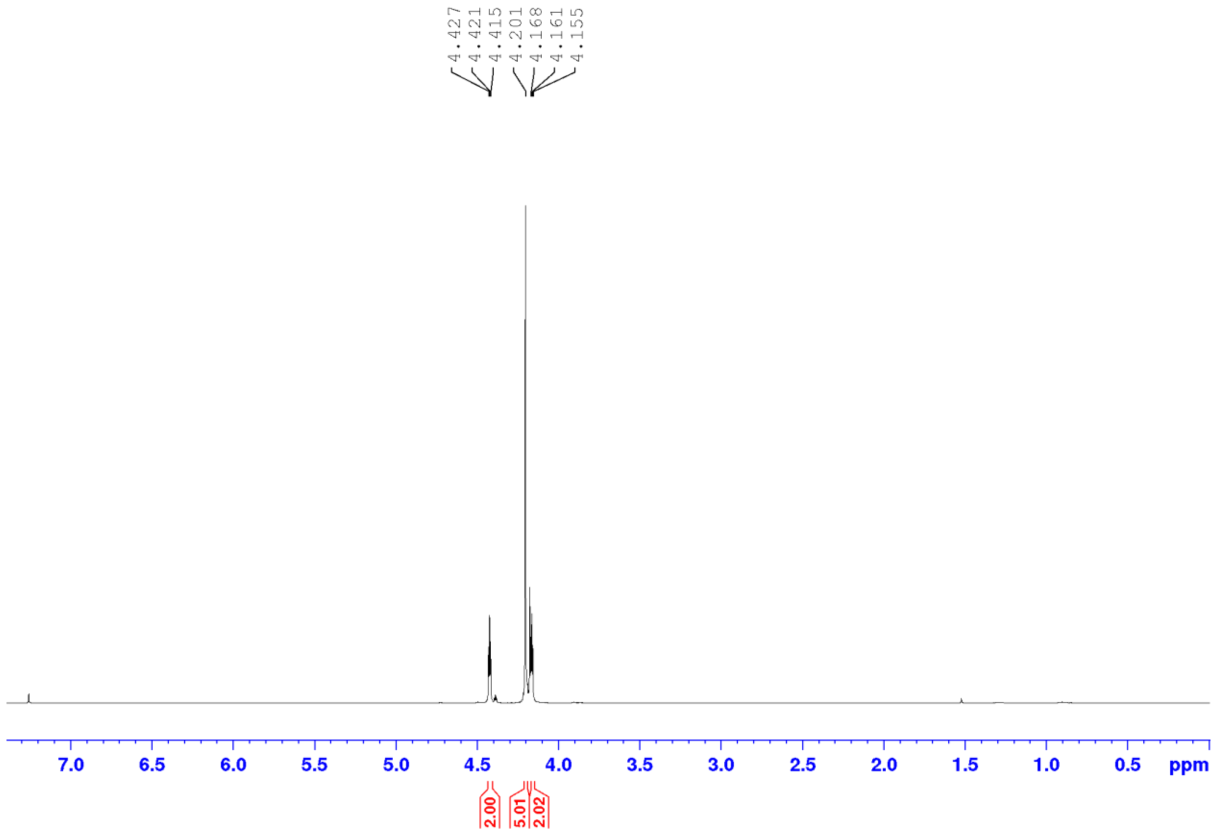

Figure S 12 ^{1}H NMR spectra of 1-iodoferrocene **2f** in CDCl$_3$.

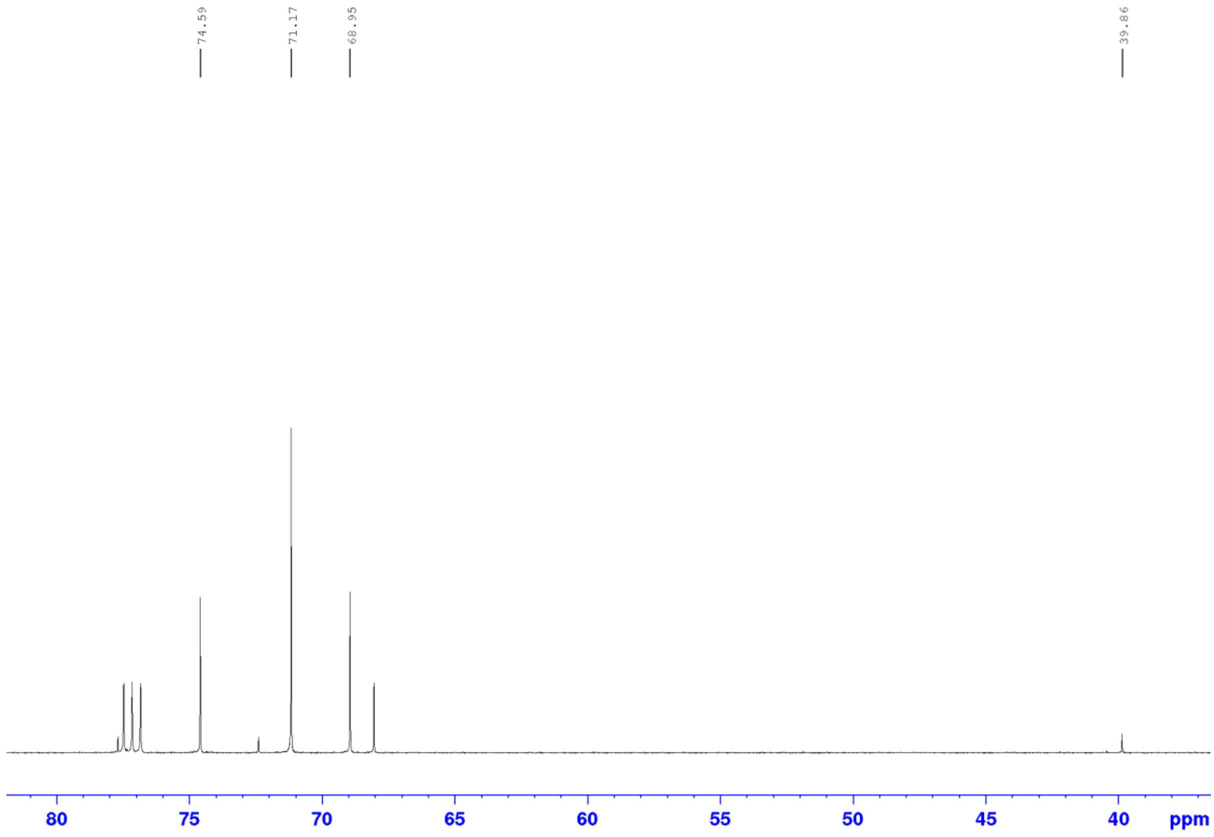

Figure S 13 $^{13}C\{^1H\}$ NMR spectra of 1-iodoferrocene **2f** in $CDCl_3$.

Synthesis of 3-iodo and 4-iodo-trimethyl(phenyl)silane **2g**

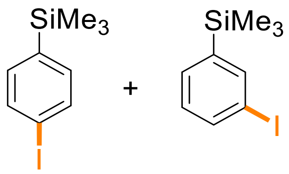

Reaction carried out following the general procedure using 5 mL trimethyl(phenyl)silane stirring the reaction for 24 hours at room temperature giving a greyish solution before quenching with iodine. Yield was determined using hexamethylbenzene (10 mol%) as an internal standard affording a mixture of 3-iodo-trimethyl(phenyl)silane (25%) and 4-iodo-trimethyl(phenyl)silane (37%). 3-iodo-trimethyl(phenyl)silane, **^{1}H-NMR (300.1 MHz, $CDCl_3$, 298 K):** δ / ppm = 7.81 (s, 1H, Ar-H), 7.70 (m, 1H, Ar-H), 7.47 (dt, J = 7.2 Hz, 1H, Ar-H), 7.10 (t, J = 7.61, 1H, Ar-H), 0.28 (s, 9H, $SiMe_3$). NMR spectroscopic data is consistent with literature reports.[11] 4-iodo-trimethyl(phenyl)silane, **^{1}H-NMR (300.1 MHz, $CDCl_3$, 298 K):** δ / ppm = 7.68 (d, J = 8.13 Hz, 2H, Ar-H), 7.24 (d, J = 8.12 Hz, 2H, Ar-H), 0.25 (s, 9H, $SiMe_3$).[12]

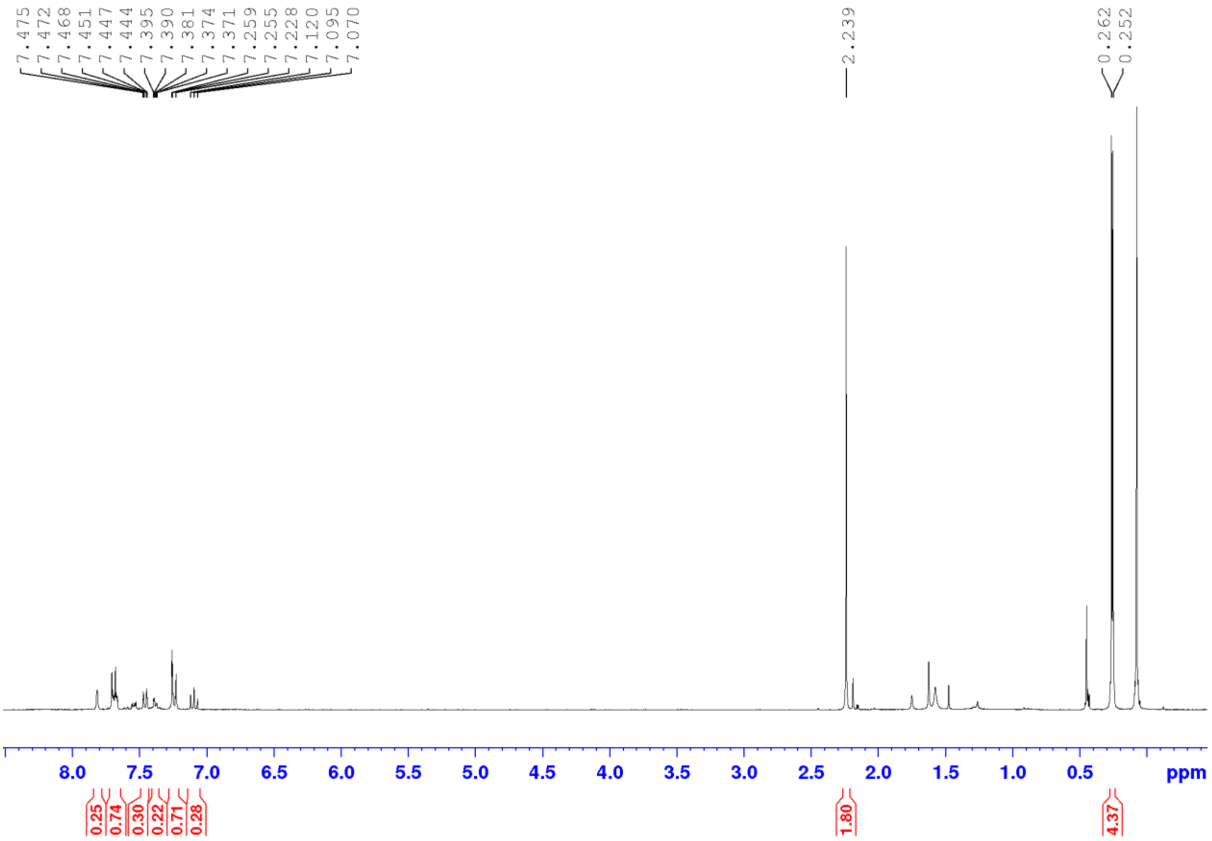

Figure S 14 ^{1}H-NMR spectra of 3-iodo and 4-iodo-trimethyl(phenyl)silane **2g** in $CDCl_3$ with 10 mol% hexamethylbenzene as internal standard.

Synthesis of 2-iodobenzoxazole **2i**

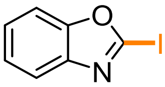

2i was synthesised according to the general procedure using 1,3-benzoxazole (2 e.q, 120 mg, 1 mmol) stirring the reaction at room temperature for 20 mins giving a bright red solution before quenching with iodine. Crude reaction mixture purified by column chromatography (hexane/EtOAc, 90:10) affording 2-iodobenzoxazole **2i** as a yellow powder (225 mg, 92%). Note that upon standing **2i** rapidly turns to a brownish powder. NMR spectroscopic data consistent with literature reports.[13] **^{1}H-NMR (300.1 MHz, $CDCl_3$, 298 K):** δ / ppm = 7.73-7.69 (m, 1H, Ar-H), 7.57-7.53 (m, 1H, Ar-H), 7.33-7.30 (m, 2H, Ar-H). **^{13}C{^{1}H}-NMR (101 MHz, $CDCl_3$, 298 K):** δ / ppm = 154.2 (s, C_q), 142.8 (s, C_q), 125.4 (s, C_{Ar}-H), 124.8 (s, C_{Ar}-H), 119.4 (s, C_{Ar}-H), 110.2 (s, C_{Ar}-H), 108.2 (s, 2 x $C_{Cp\text{-}I}$).

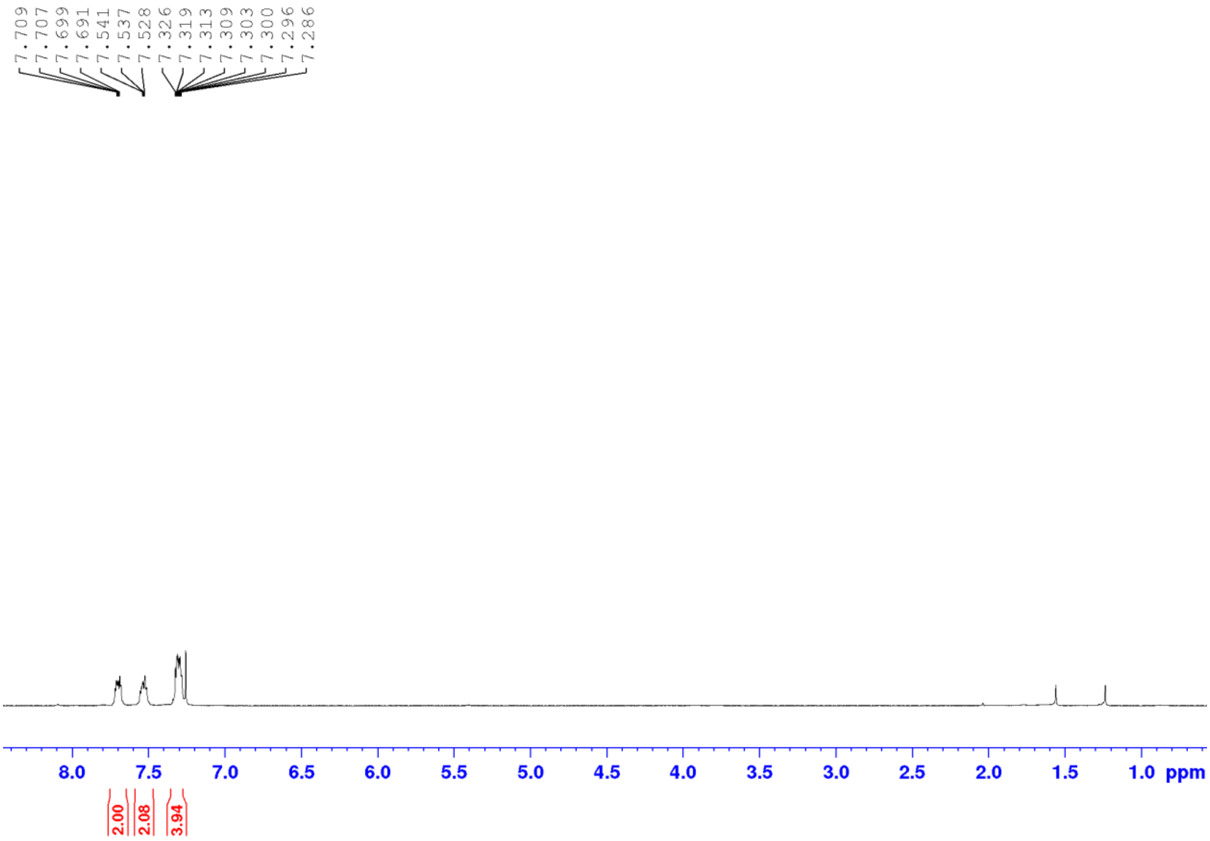

Figure S 15 ^{1}H NMR spectra of 2-iodobenzoxazole **2i** in $CDCl_3$.

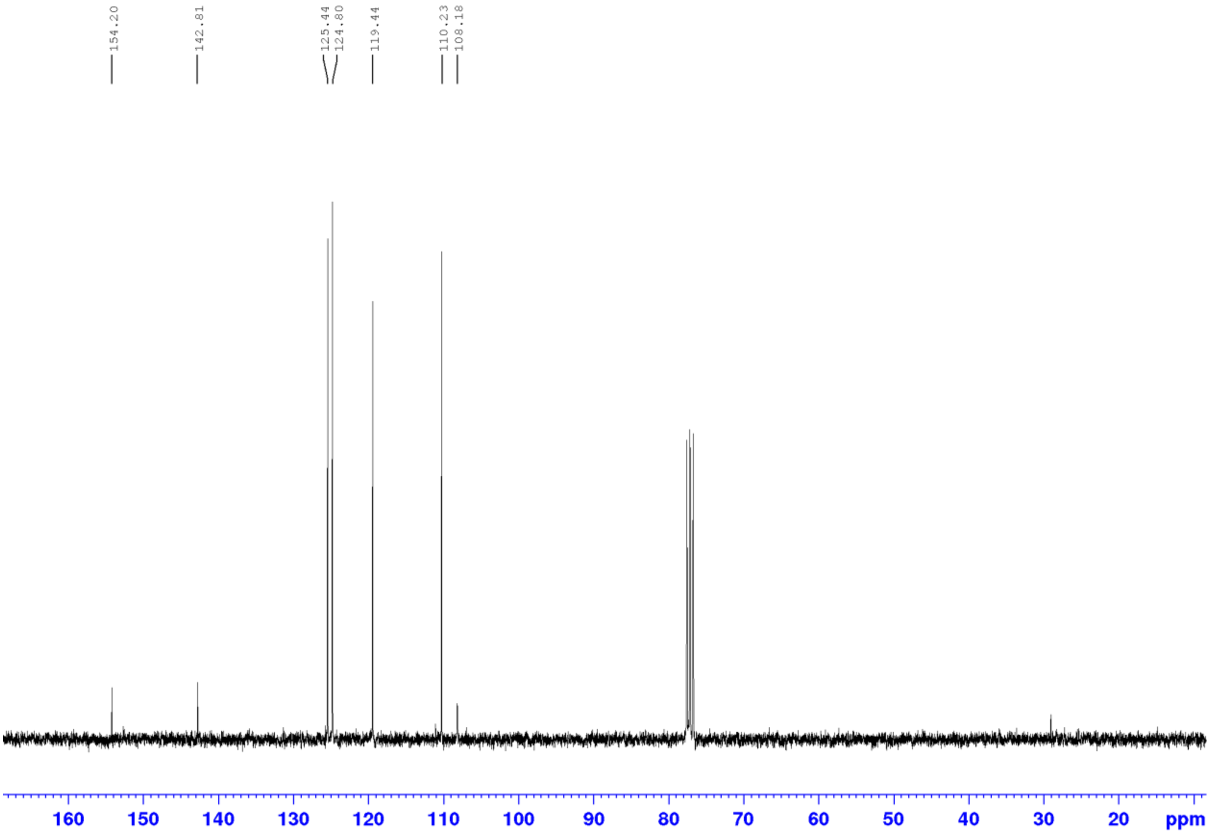

Figure S 16 $^{13}C\{^{1}H\}$ NMR spectra of 2-iodobenzoxazole **2i** in $CDCl_3$.

Synthesis of 2-iodobenzothiazole **2j**

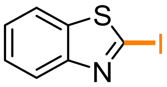

2j was synthesised according to the general procedure using 1,3-benzothiazole (2 e.q, 110 µL, 1 mmol) stirring the reaction at room temperature for 20 mins giving a yellow solution before quenching with iodine. Crude reaction mixture purified by column chromatography (hexane/EtOAc, 80:20) affording 2-iodobenzothiazole **2j** as a yellow powder (230 mg, 88%). NMR spectroscopic data consistent with literature reports.[13] **^{1}H-NMR (300.1 MHz, $CDCl_3$, 298 K):** δ / ppm = 8.03 (dd, J = 8.32 Hz, 1.73 Hz, 1H, Ar-H), 7.85 (dd, J = 7.63 Hz, J = 1.72, 1H, Ar-H), 7.46-7.35 (m, 2H, Ar-H). **^{13}C{^{1}H}-NMR (101 MHz, $CDCl_3$, 298 K):** δ / ppm = 154.4 (s, C_q), 139.3 (s, C_q), 126.5 (s, C_{Ar}-H), 125.7 (s, C_{Ar}-H), 122.7 (s, C_{Ar}-H), 120.5 (s, C_{Ar}-H), 105.8 (s, C_q-I).

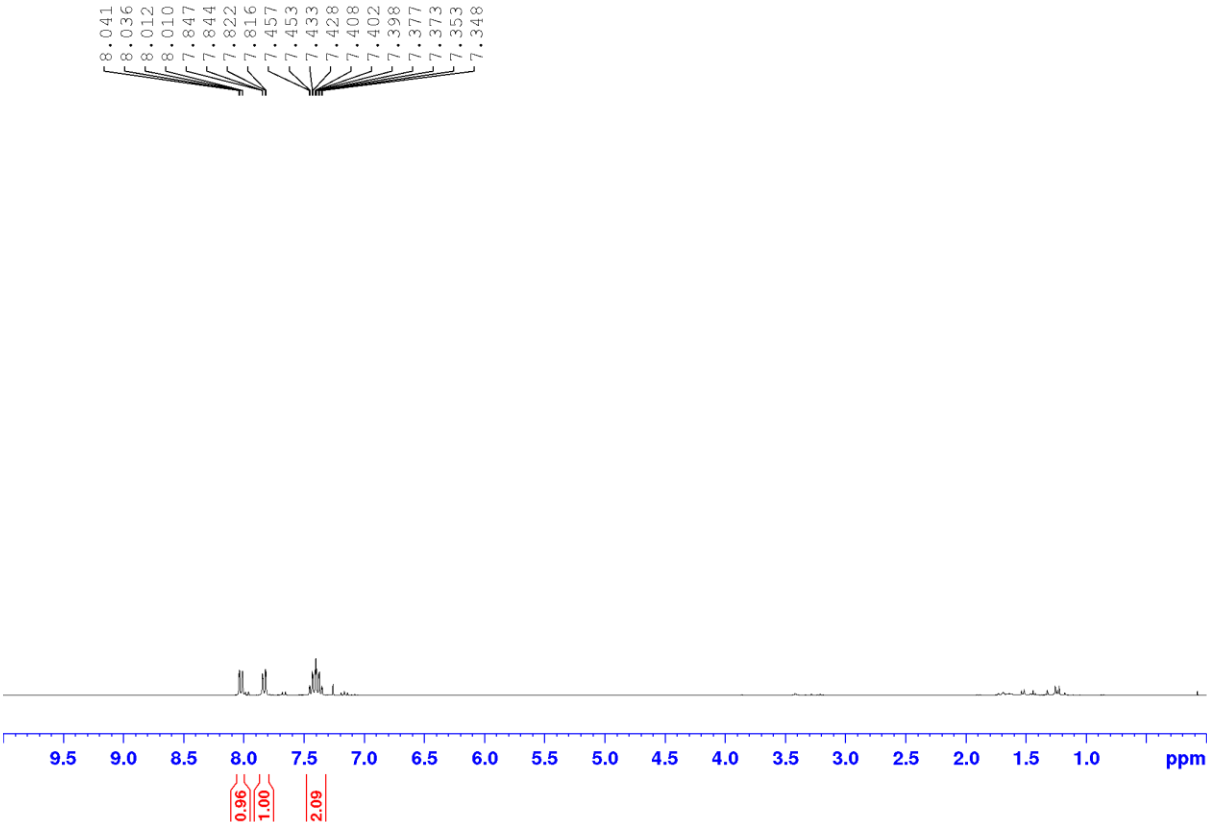

Figure S 17 ^{1}H NMR spectra of 2-iodobenzothiazole **2j** in $CDCl_3$.

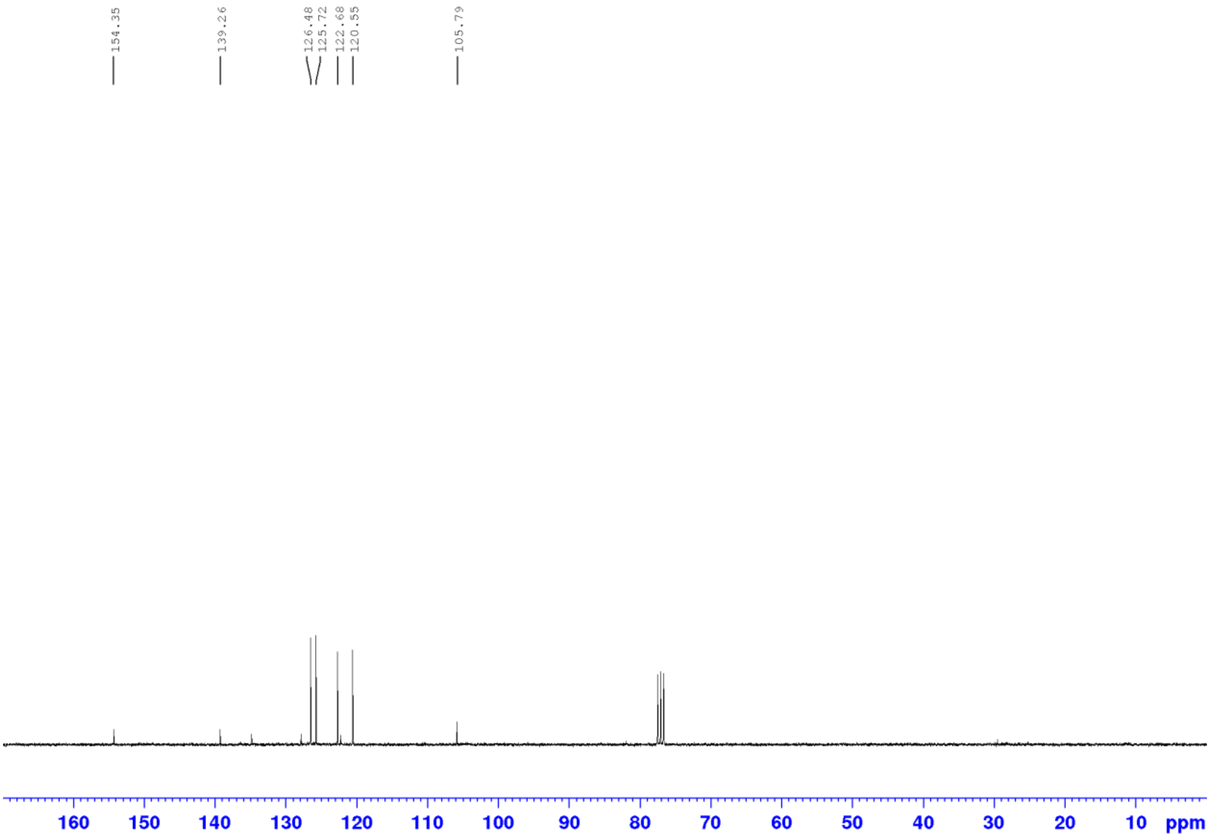

Figure S 18 $^{13}C\{^1H\}$ NMR spectra of 2-iodobenzothiazole **2j** in $CDCl_3$.

<u>Synthesis of 2-iodobenzothiophene **2k**</u>

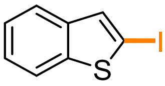

2k was synthesised according to the general procedure using benzothiophene (2 e.q, 134 mg, 1 mmol) stirring the reaction at room temperature for 20 mins giving a yellow solution before quenching with iodine. Crude reaction mixture purified by column chromatography (hexane 100%) affording 2-iodobenzothiophene **2k** as a light-yellow powder (250 mg, 96%). NMR spectroscopic data consistent with literature reports.[13] **^{1}H-NMR (300.1 MHz, $CDCl_3$, 298 K):** δ / ppm = 7.78-7.70 (m, 2H, Ar-H), 7.54 (s, 1H, Ar-H), 7.31-7.21 (m, 2H, Ar-H). **$^{13}C\{^1H\}$-NMR (101 MHz, $CDCl_3$, 298 K):** δ / ppm = 144.5 (s, C_q), 140.9 (s, C_q), 133.9 (s, C_{Ar}-H), 124.6 (s, C_{Ar}-H), 124.5 (s, C_{Ar}-H), 122.4 (s, C_{Ar}-H), 121.4 (s, C_{Ar}-H), 78.5 (s, C_q-I).

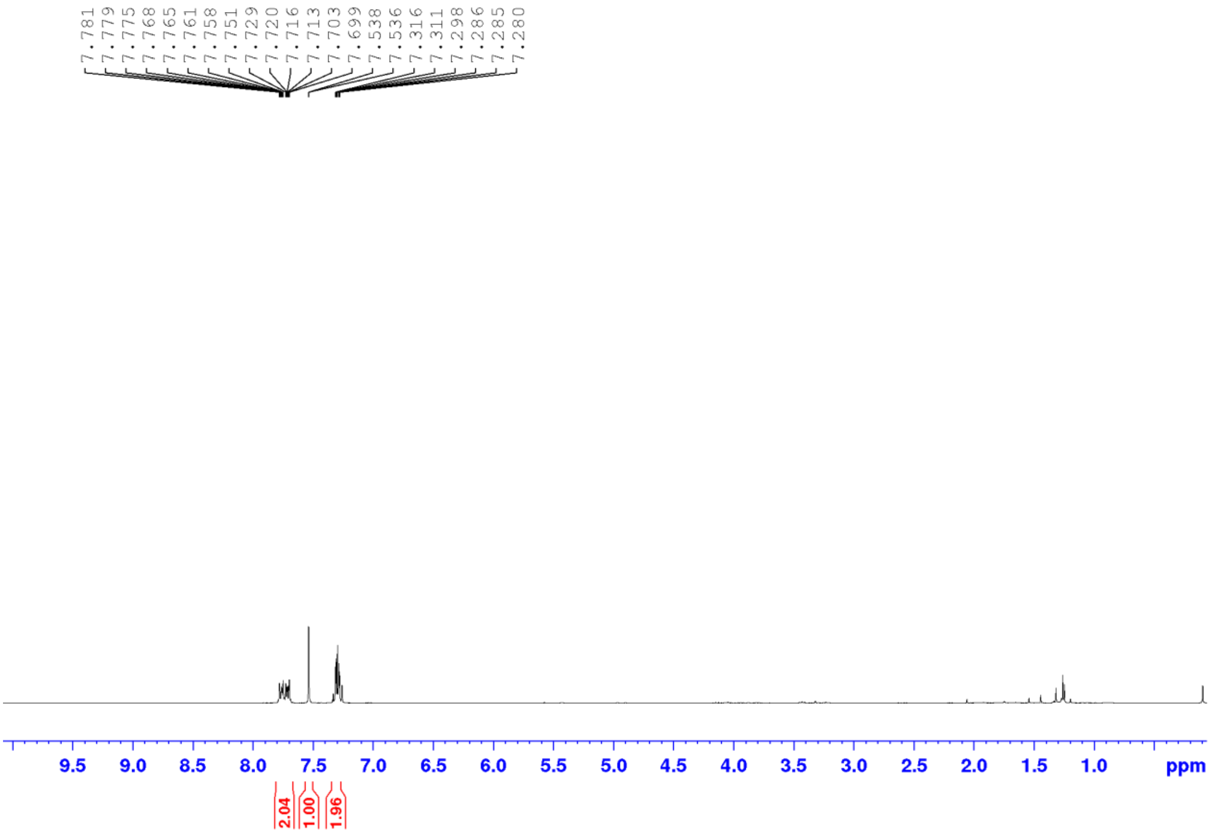

Figure S 19 ^{1}H NMR spectra of 2-iodobenzothiophene **2k** in $CDCl_3$.

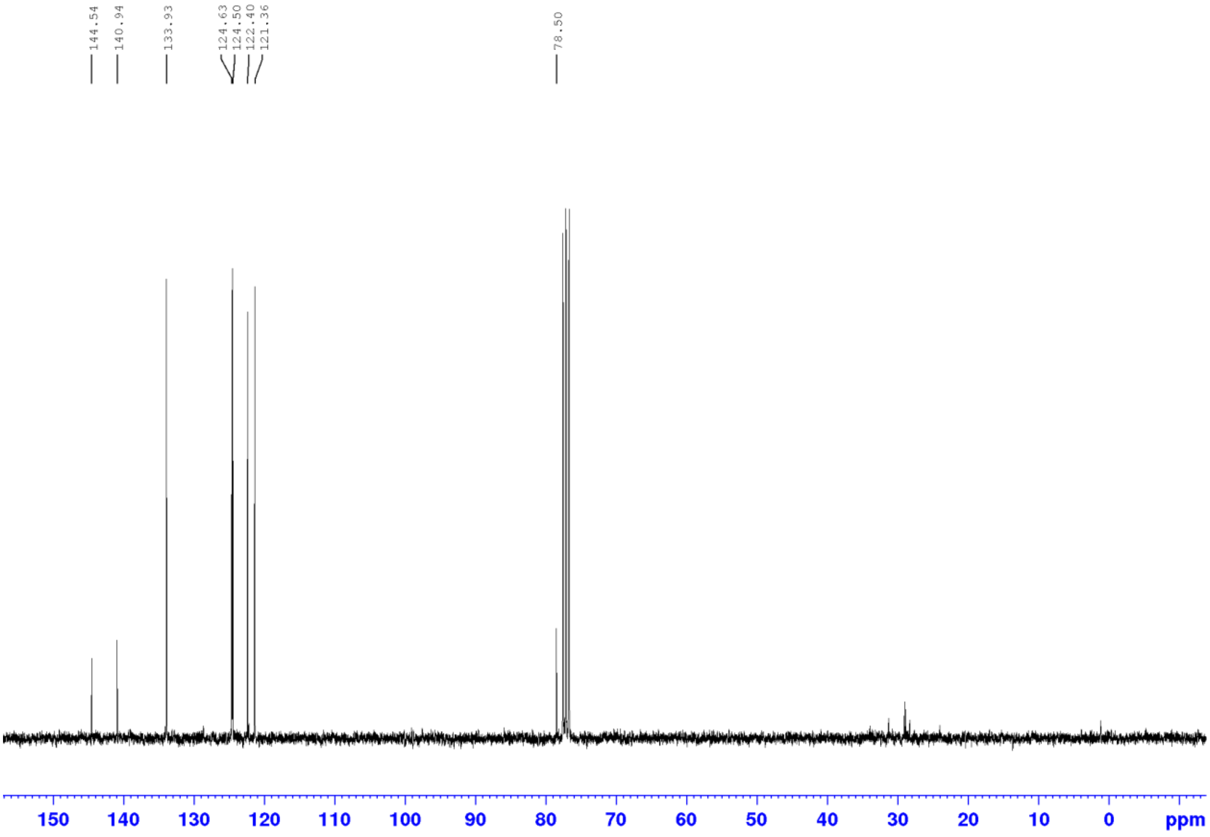

Figure S 20 $^{13}C\{^1H\}$ NMR spectra of 2-iodobenzothiophene **2k** in $CDCl_3$.

Synthesis of 2-iodobenzofuran **2l**

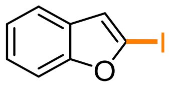

2l was synthesised according to the general procedure using benzofuran (2 e.q, 110 µL, 1 mmol) stirring the reaction at room temperature for 20 mins giving a yellow solution before quenching with iodine. Crude reaction mixture purified by column chromatography (hexane 100%) yield of 2-iodobenzofuran **2l** as a yellow oil (185 mg, 81%). NMR spectroscopic data consistent with literature reports.[13] **^{1}H-NMR (300.1 MHz, $CDCl_3$, 298 K):** δ / ppm = 7.53-7.46 (m, 2H, Ar-H), 7.23-7.20 (m, 2H, Ar-H), 6.96 (d, J = 0.81 Hz, 1H, Ar-H). **^{13}C{^{1}H}-NMR (101 MHz, $CDCl_3$, 298 K):** δ / ppm = 158.4 (s, C_q), 129.4 (s, C_q), 124.4 (s, C_{Ar}-H), 123.3 (s, C_{Ar}-H), 119.8 (s, C_{Ar}-H), 117.4 (s, C_{Ar}-H), 110.9 (s, C_{Ar}-H), 95.9 (s C_q-I).

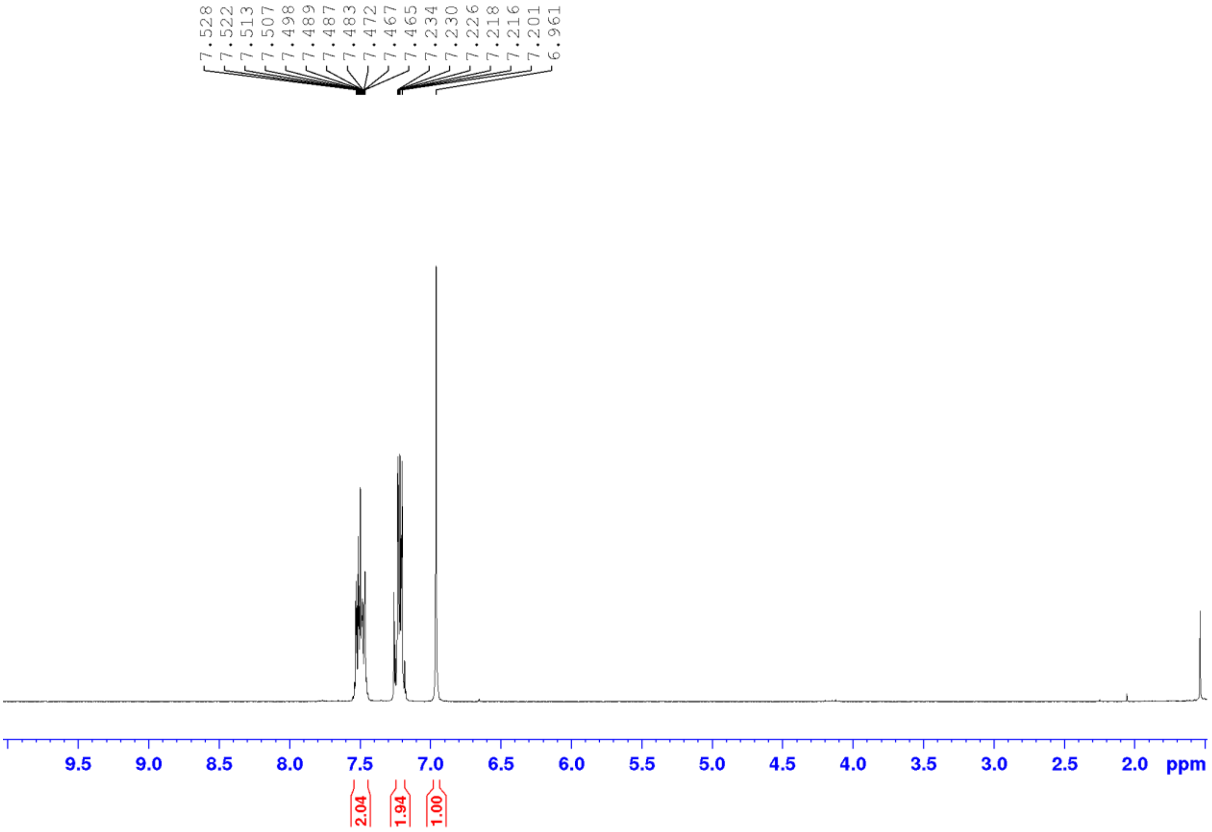

Figure S 21 ^{1}H NMR spectra of 2-iodobenzofuran **2l** in $CDCl_3$.

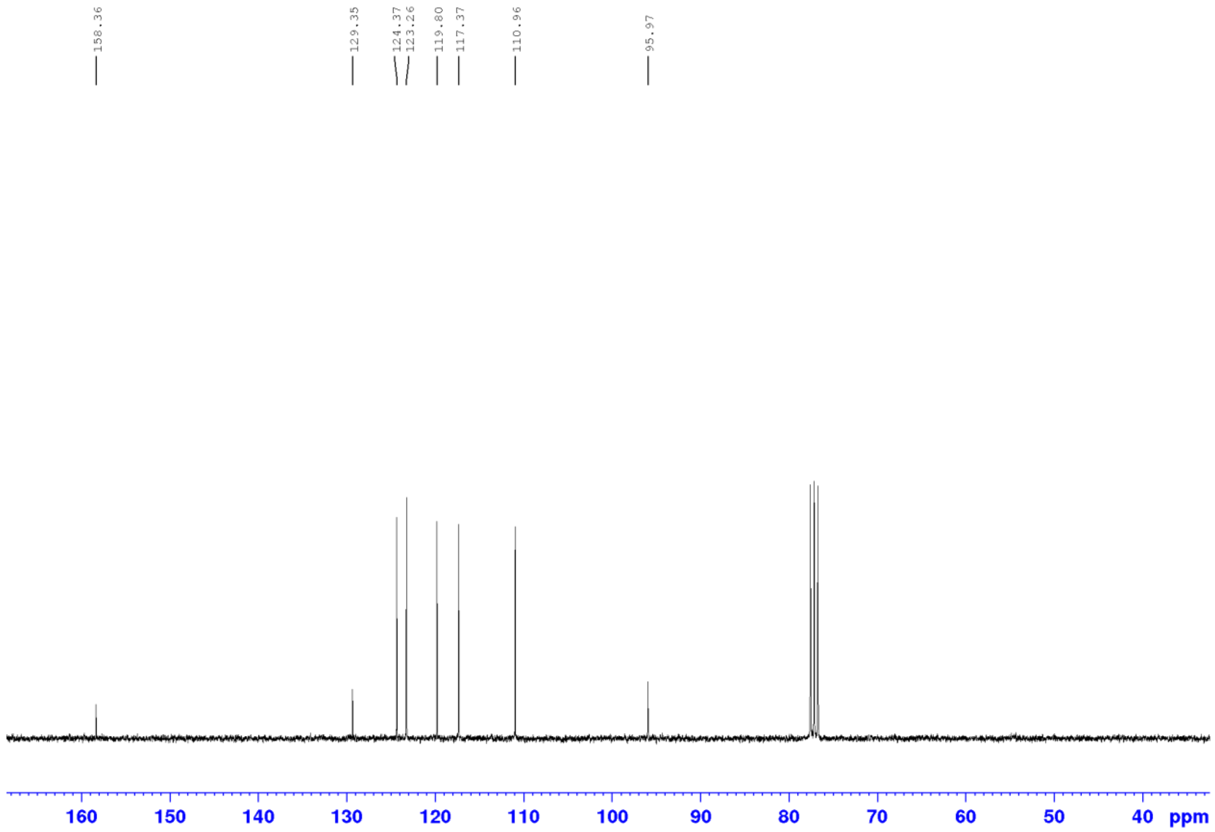

Figure S 22 $^{13}C\{^1H\}$ NMR spectra of 2-iodobenzofuran **2l** in $CDCl_3$.

Synthesis of 2-iodo-1-methylbenzimidazole **2m**

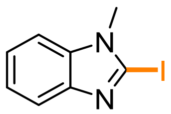

2m was synthesised according to the general procedure using 1-methylbenzimidazole (2 e.q, 132 mg, 1 mmol) stirring the reaction at room temperature for 20 mins giving a yellow solution before quenching with iodine. Crude reaction mixture purified by column chromatography (EtOAc, 100%) affording 2-iodo-1-methylbenzimidazole **2m** as a yellow powder (210 mg, 81%). NMR spectroscopic data consistent with literature reports.[14] **^{1}H-NMR (300.1 MHz, $CDCl_3$, 298 K):** δ / ppm = 7.64-7.60 (m, 1H, Ar-H), 7.25-7.22 (m, 1H, Ar-H), 7.18-7.11 (m, 2H, Ar-H), 3.67 (s, 3H, NMe). **$^{13}C\{^1H\}$-NMR (101 MHz, $CDCl_3$, 298 K):** δ / ppm = 145.6 (s, C_q), 136.4 (s, C_q), 123.2 (s, C_{Ar}-H), 122.3 (s, C_{Ar}-H), 119.3 (s, C_{Ar}-H), 109.5 (s, C_{Ar}-H), 104.4 (s, C_q-I), 33.8 (s, N*Me*).

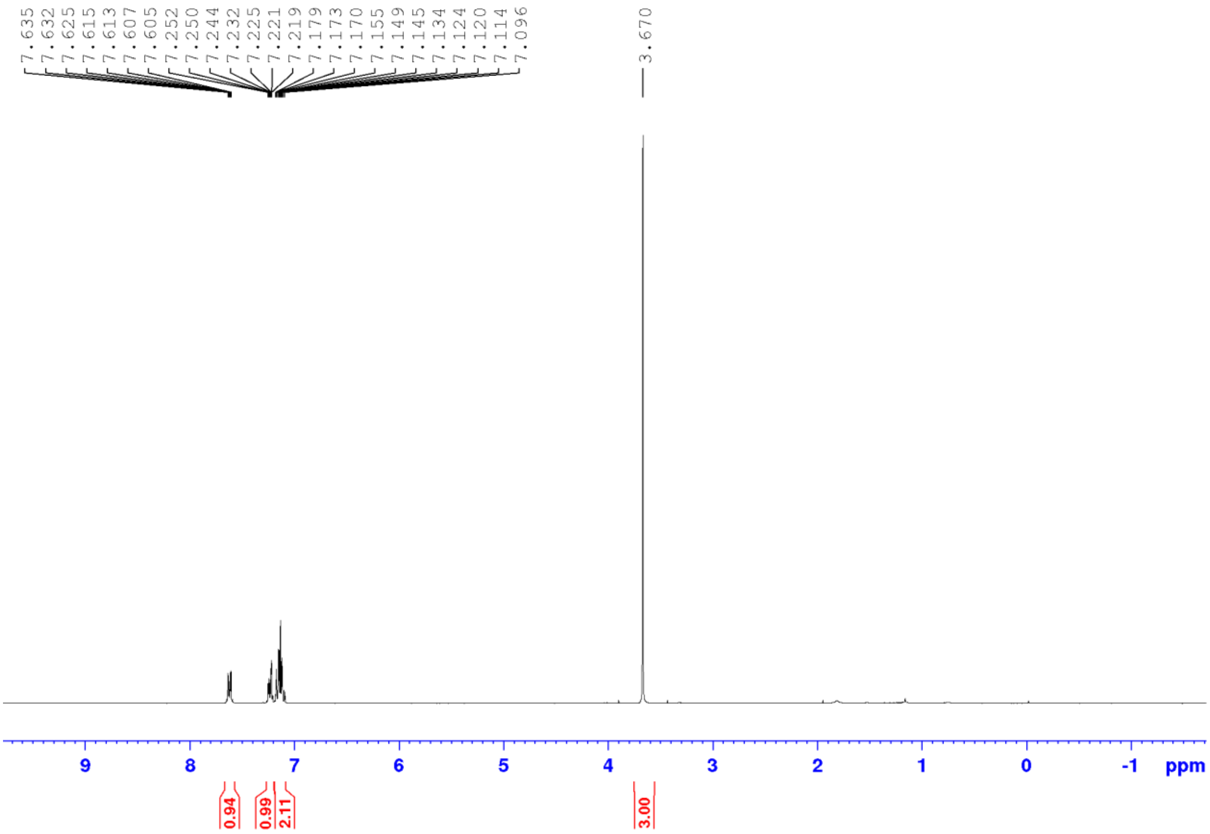

Figure S 23 ^{1}H NMR spectra of 2-iodo-1-methylbenzimidazole **2m** in $CDCl_3$.

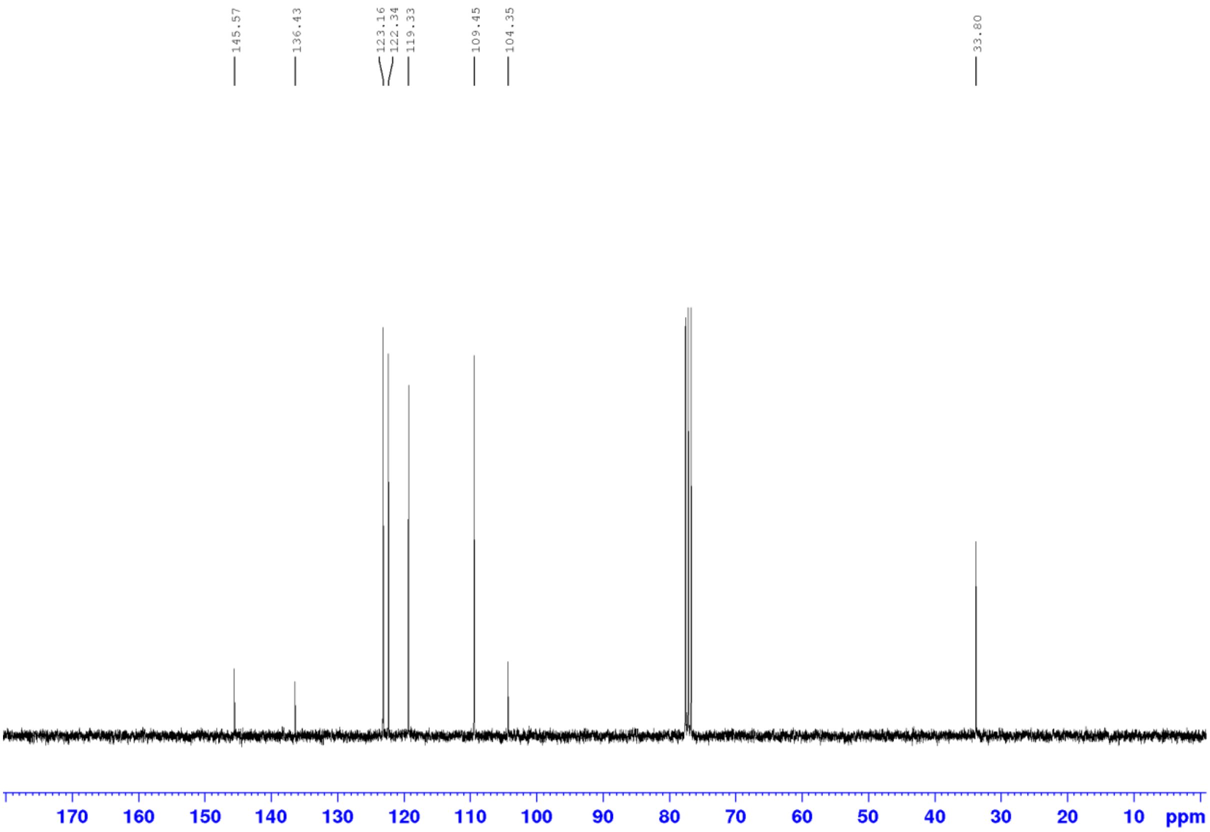

Figure S 24 $^{13}C\{^{1}H\}$ NMR spectra of 2-iodo-1-methylbenzimidazole **2m** in $CDCl_3$.

Synthesis of 5-iodo-1-methyl-1,2,4-triazole **2n**

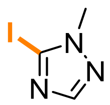

2n was synthesised according to the general procedure using 1-methyl-1,2,4-triazole (2 e.q, 57 µL, 1 mmol) stirring the reaction at room temperature for 20 mins giving a yellow solution before quenching with iodine. Crude reaction mixture purified by column chromatography (hexane/EtOAc 40:60 to 60:40) affording 5-iodo-1-methyl-1,2,4-triazole **2n** as an off-white solid (190 mg, 91%). NMR spectroscopic data consistent with literature reports.[15] **^{1}H-NMR (300.1 MHz, $CDCl_3$, 298 K):** δ / ppm = 7.88 (s, 1H, Ar-H), 3.90 (s, 3H, NMe). **^{13}C{^{1}H}-NMR (101 MHz, $CDCl_3$, 298 K):** δ / ppm = 154.3 (s, C_{Ar}-H), 100.2 (s, C_q-I), 38.0 (s, N*Me*).

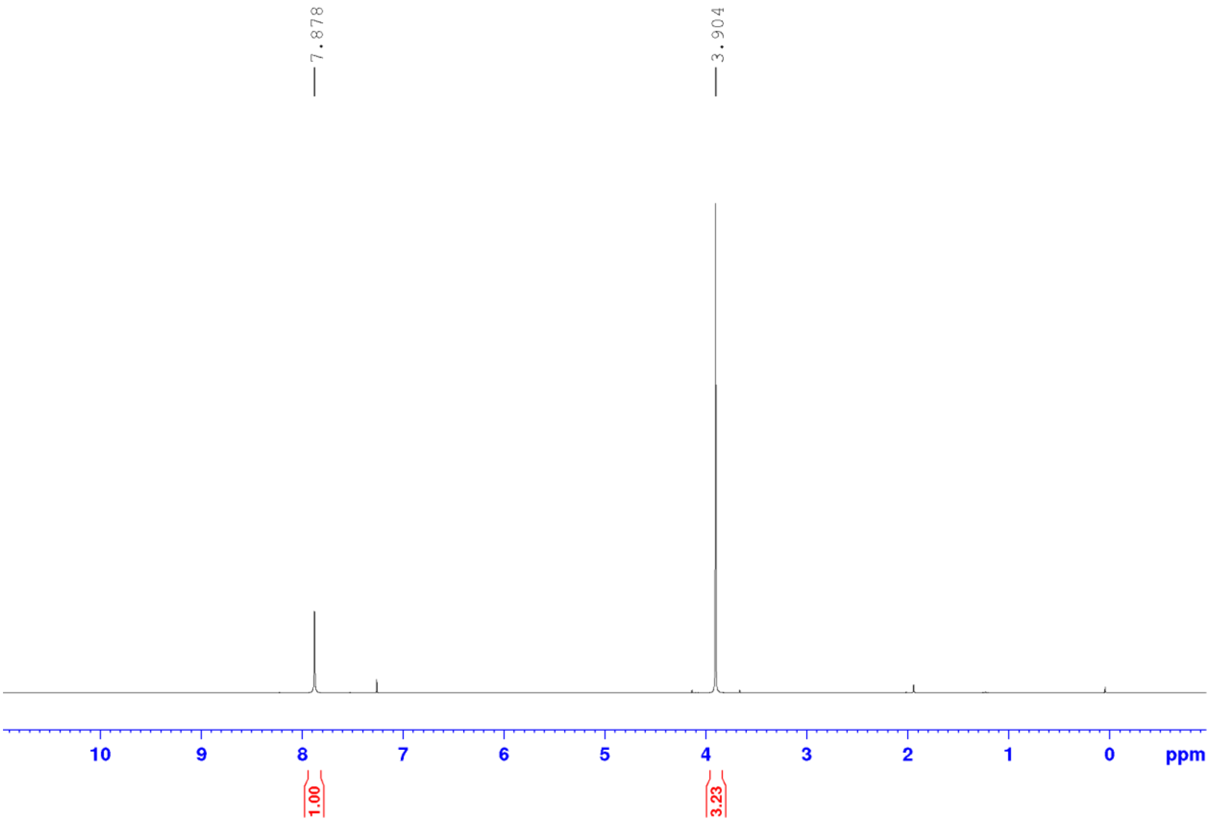

Figure S 25 ^{1}H NMR spectra of 5-iodo-1-methyl-1,2,4-triazole **2n** in $CDCl_3$.

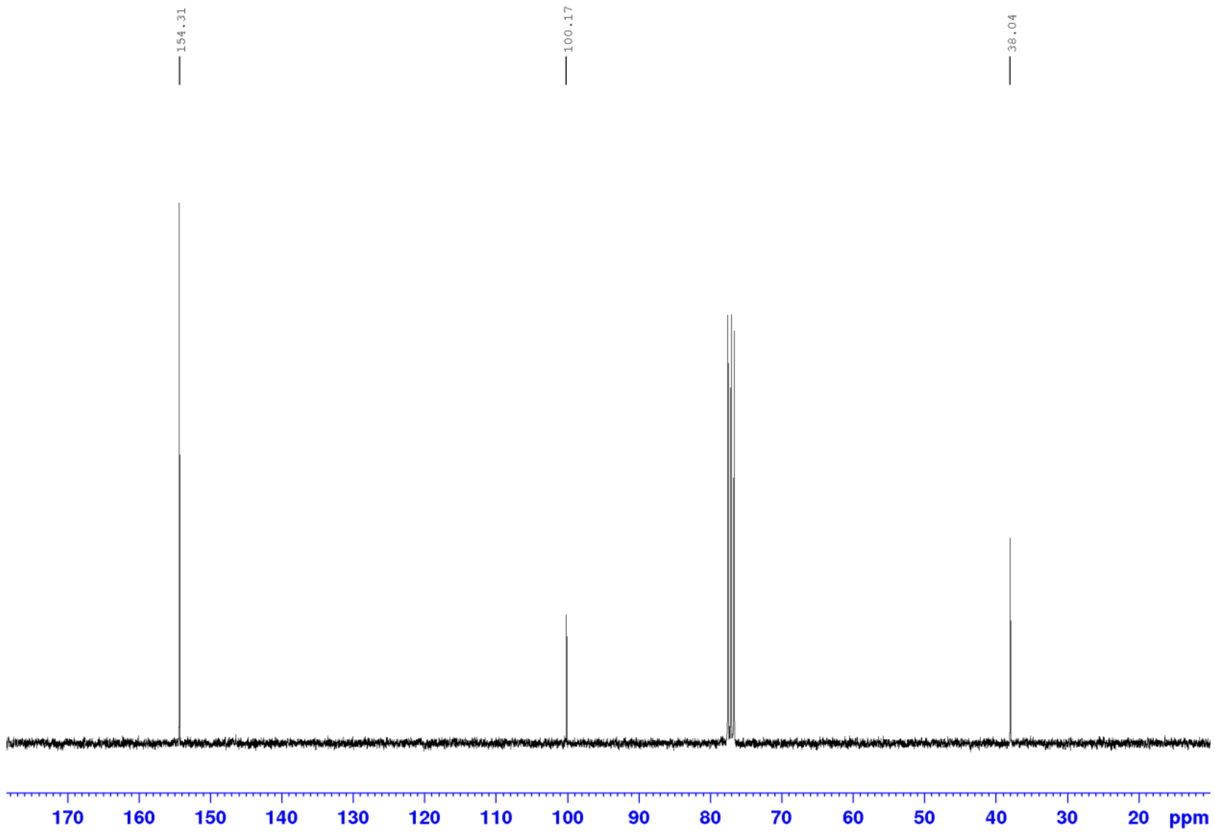

Figure S 26 $^{13}C\{^{1}H\}$ NMR spectra of 5-iodo-1-methyl-1,2,4-triazole **2n** in $CDCl_3$.

<u>Synthesis of iodocaffeine **2o**</u>

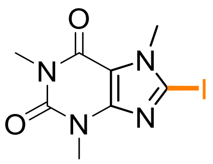

2o was synthesised according to the general procedure using caffeine (2 e.q, 194 mg, 1 mmol) stirring the reaction at room temperature for 10 mins giving a white suspension before quenching with iodine. Crude reaction mixture filtered through a plug of silica gel and washed with DCM affording iodocaffeine **2o** as a white solid (270 mg, 84%). NMR spectroscopic data consistent with literature reports.[16] **^{1}H-NMR (300.1 MHz, $CDCl_3$, 298 K):** δ / ppm = 3.94 (s, CH_3, NMe), 3.55 (s, CH_3, NMe), 3.38 (s, CH_3, NMe). **$^{13}C\{^{1}H\}$-NMR (101 MHz, $CDCl_3$, 298 K):** δ / ppm = 154.3 (s, C_q), 151.4 (s, C_q), 149.7 (s, C_q), 110.9 (s, C_q), 101.2 (s, C_q-I), 36.3 (s, *C*H_3, N*Me*), 29.9 (s, *C*H_3, N*Me*), 28.2 (s, *C*H_3, N*Me*).

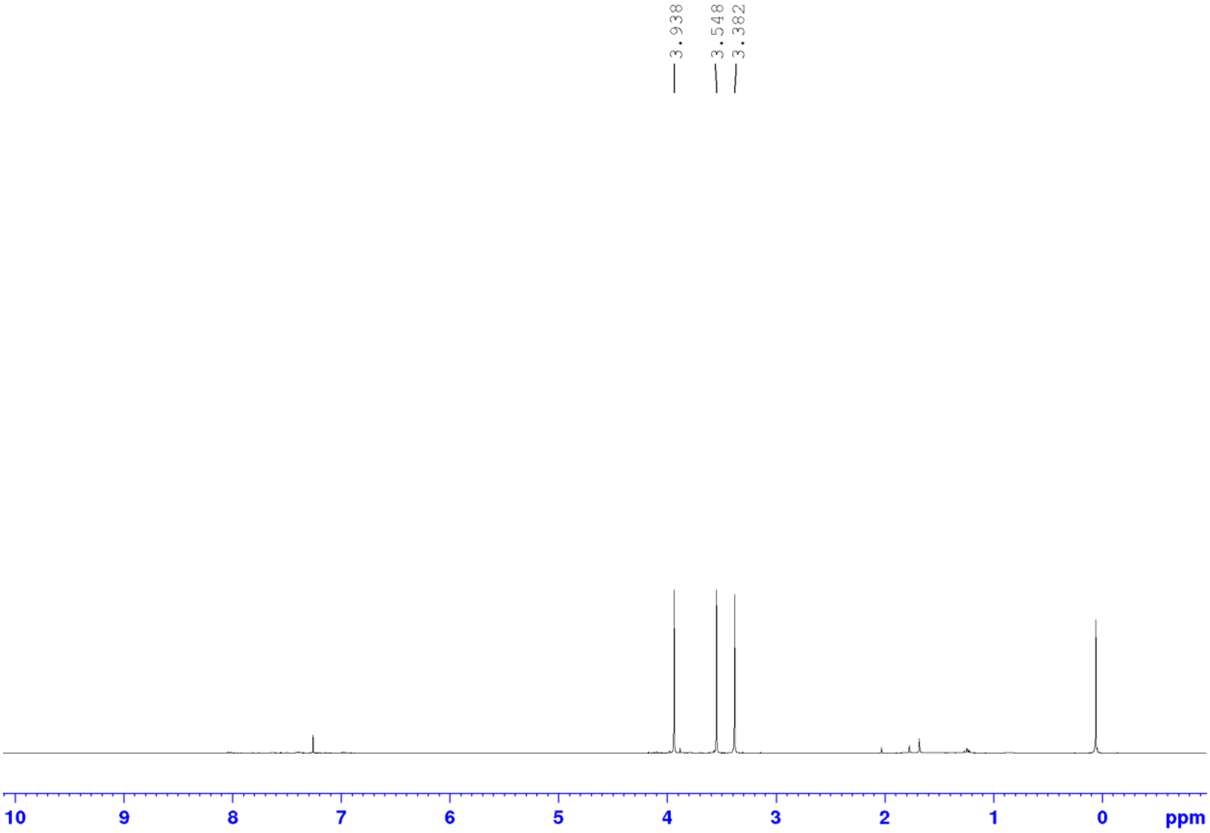

Figure S 27 ^{1}H NMR spectra of iodocaffeine **2p** in $CDCl_3$.

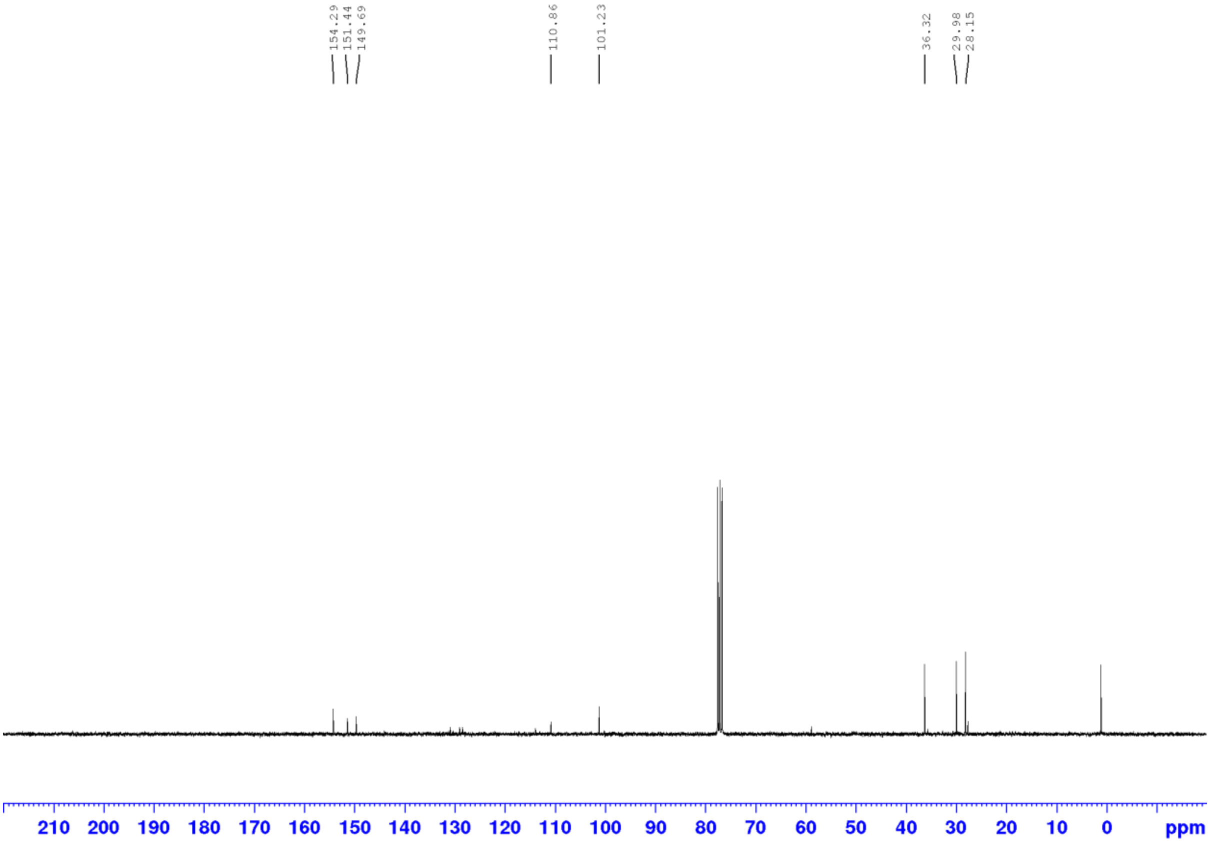

Figure S 28 $^{13}C\{^1H\}$ NMR spectra of iodocaffeine **2o** in $CDCl_3$.

3.4 Synthesis of organometallic compounds **3a, 3b, Ib, 3c, 4, 5, and 7**

Synthesis of $[(THF)_2KZn(C_{10}H_7)(OtBu)_2]_2$ (**3a**)

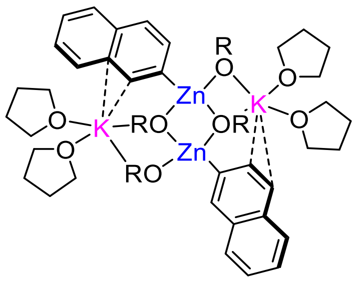

To an argon flushed Schlenk flask, $Zn(TMP)_2$ (350 mg, 1 mmol), KO*t*Bu (220 mg, 2 mmol) and naphthalene (260 mg, 2 mmol) were dissolved in THF (5 mL). This solution was allowed to stir at room temperature for 2 hours affording a bright yellow solution. The solvent was reduced to approximately 0.5 mL and hexane was layered on top of the THF solution. The solution was then stored at -30 °C for 4 days affording a colourless crop of crystals – compound **3a**. Yield 300 mg, 29%. **^{1}H-NMR (300.1 MHz, D_8-THF, 298 K):** δ / ppm = 8.15 (s, 1H, Ar-H), 7.91 (d, J = 7.32 Hz, 1H, Ar-H), 7.60 (t, J = 8.75 Hz, 2H, Ar-H), 7.49 (d, J = 7.3 Hz, 1H, Ar-H), 7.18 (m, 2H, Ar-H), 1.22 (s, 18H, 2 x O*t*Bu). **^{13}C{^{1}H}-NMR (101 MHz, D_8-THF, 298 K):** δ / ppm = 163.0 (s, C_q-Zn), 138.6 (s, Ar, C-H), 134.4 (s, Ar, C-H), 134.3 (s, Ar, C-H), 132.9 (s, Ar, C-H), 127.8 (s, Ar, C-H), 127.7 (s, Ar, C-H). 124.4 (s, Ar, C-H), 124.0 (s, Ar, C-H), 123.3 (s, Ar, C-H), 35.5 (s, CH_3, 2 x O*t*Bu). Repeated washing with hexane to remove excess naphthalene (formed from the decomposition of potassium naphthalenide with THF, not incomplete metalation) proved unsuccessful therefore signals for naphthalene are present in the ^{1}H and ^{13}C NMR spectra.

Note: Compound **3a** could not be accessed from direct zincation of naphthalene with *in-situ* prepared $[(THF)_nKZn(TMP)(OtBu)_2]$ (prepared via addition of one equivalent of *t*BuOH to $KZn(TMP)_2OtBu$).

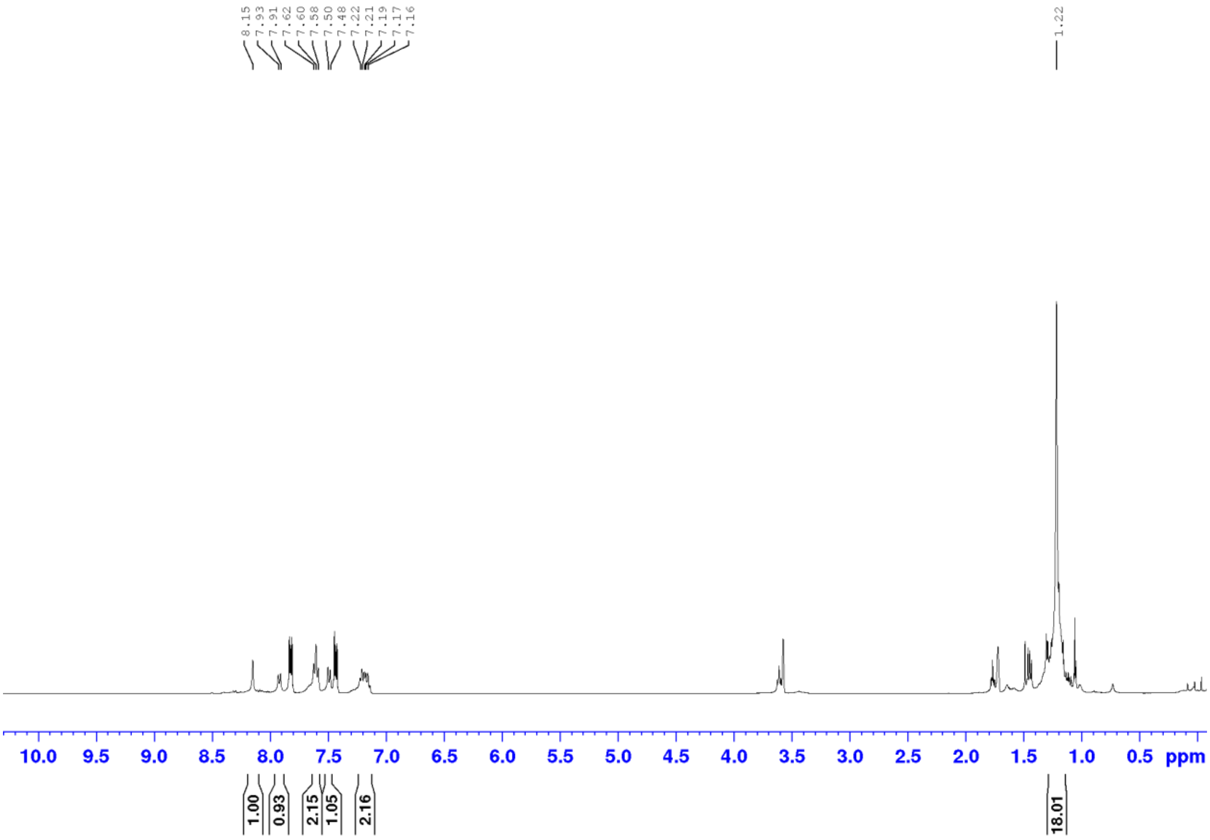

Figure S 29 ^{1}H-NMR spectra of [(THF)$_2$KZn(C$_{10}$H$_7$)(O*t*Bu)$_2$]$_2$ **3a** in d$_8$-THF.

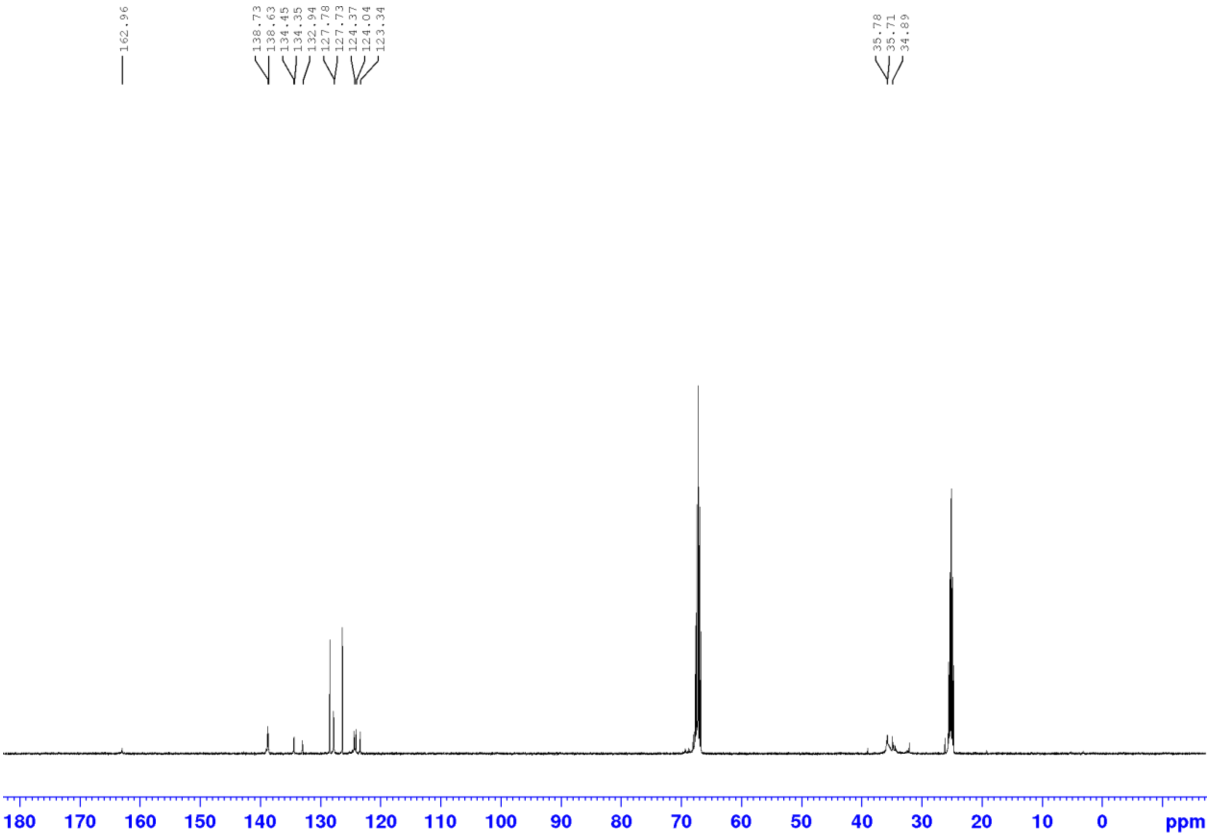

Figure S 30 ^{13}C{^{1}H} NMR spectra of [(THF)$_2$KZn(C$_{10}$H$_7$)(O*t*Bu)$_2$]$_2$ **3a** in d$_8$-THF.

Synthesis of $[(THF)_2KZn(C_6H_5)(OtBu)_2]_2$ (**3b**)

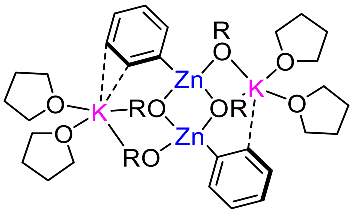

To an argon flushed Schlenk flask, $Zn(TMP)_2$ (180 mg, 0.5 mmol), KO*t*Bu (110 mg, 1 mmol) were dissolved in benzene (5 mL) and left to stir at room temperature for 24 hours. All solvent was removed under vacuum and the resulting white solid was suspended in hexane (5 mL). THF was dripped into solution until a pale-yellow solution was formed. The solution was then stored at -30 °C overnight affording a colourless crop of crystals – compound **3b**. Yield 110 mg, 29%. Anal. Calcd. for $C_{36}H_{64}K_2O_6Zn_2$ (2 THF molecules coordinated) C, 53.92; H, 8.05. Found: C, 53.55; H, 7.49. **^{1}H-NMR (300.1 MHz, D_8-THF, 298 K):** δ / ppm = 7.81 (br. m, 2H, Ar-H), 7.00 (t, J = 7.85 Hz, 2H, Ar-H), 6.89 (m, 1H, Ar-H), 3.61 (THF), 1.77 (THF), 1.30 (s, 9H,O*t*Bu) 1.06 (s, 9H, O*t*Bu). **^{13}C{^{1}H}-NMR (101 MHz, D_8-THF, 298 K):** δ / ppm = 165.7 (s, C_q-Zn), 140.4 (s, Ar, C-H), 126.9 (s, Ar, C-H), 124.9 (s, Ar, C-H), 68.5 (br. s, 2 x C_q, 2 x O*t*Bu), 68.0 (THF), 37.0 (s, CH_3, O*t*Bu), 35.8 (s, CH_3, O*t*Bu), 26.1 (THF). Large singlet peak at 7.31 ppm correlates to free benzene (1/3 of a molecule relative to **3b**) which could not be removed under vacuum (possibly due to coordination to the K atom and then liberated once in d_8-THF).

Note: ^{1}H-NMR spectra in D_8-THF at room temperature shows two broad peaks (δ = 1.30 and 1.06 ppm) for the O*t*Bu signals however, at 60 °C (333K) this signal coalesces to form a single broad singlet (δ = 1.15 ppm) suggesting some fluxionality of this dimer in d_8-THF solution (possibly monomer/dimer equilibrium). Hence the broad peaks in the ^{13}C-NMR at room temperature.

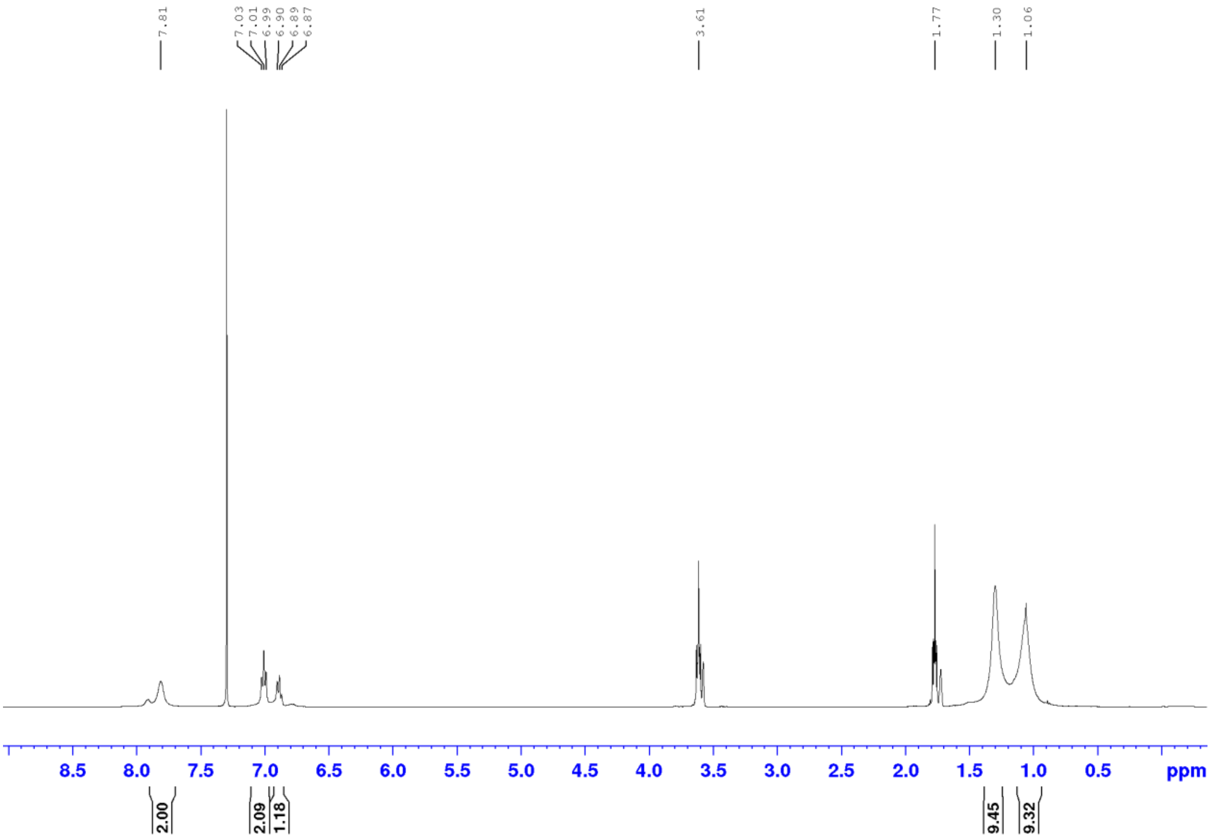

Figure S 31 ^{1}H-NMR spectra of [(THF)$_2$KZn(Ph)(O*t*Bu)$_2$]$_2$ **3b** in d$_8$-THF.

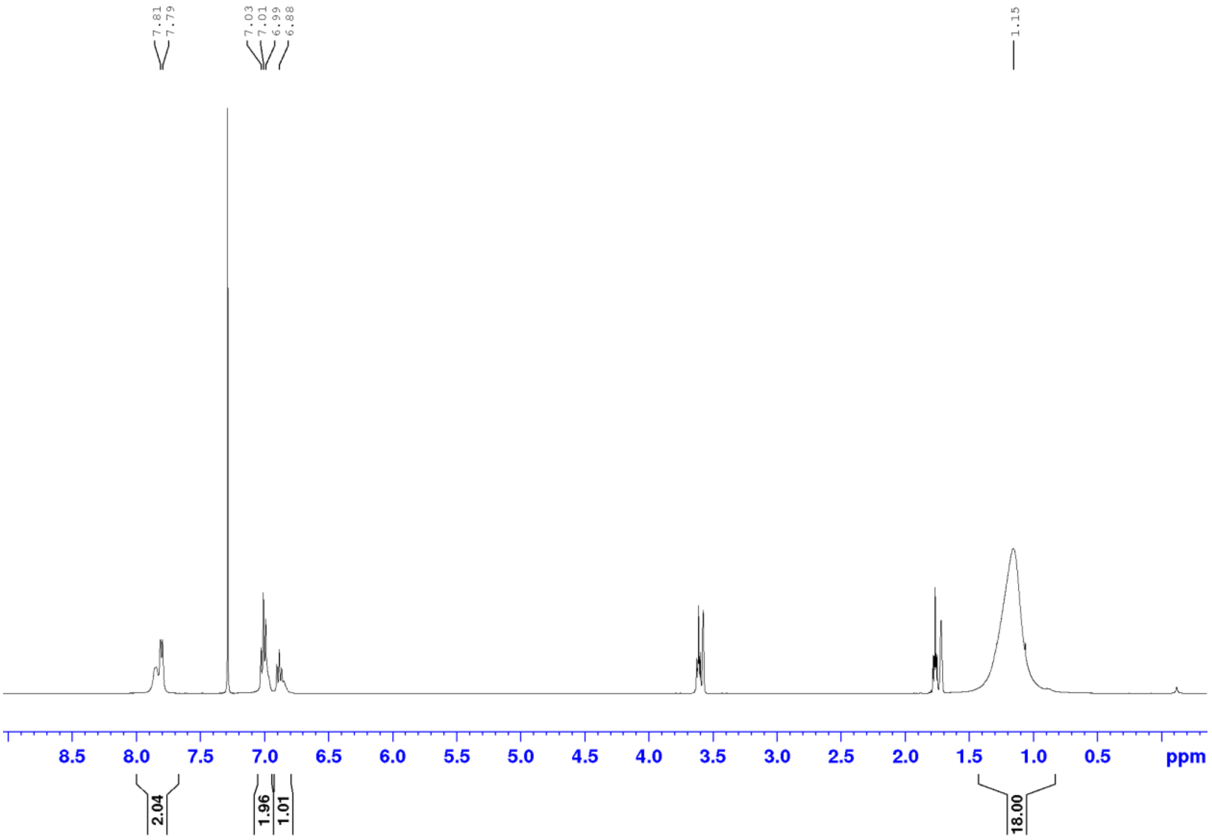

Figure S 32 1H-NMR spectra of [(THF)$_2$KZn(Ph)(O*t*Bu)$_2$]$_2$ **3b** in d8-THF at 333 K.

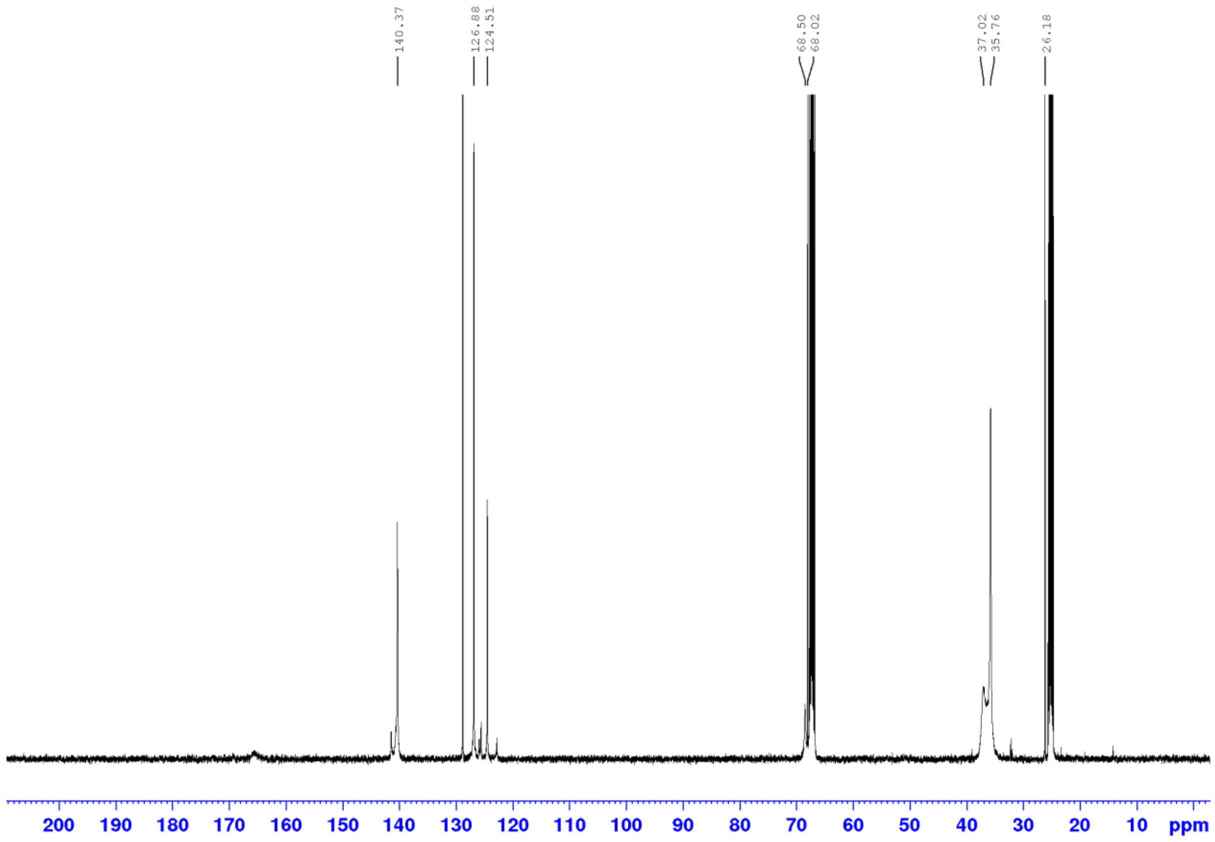

Figure S 33 $^{13}C\{^1H\}$ NMR spectra of $[(THF)_2KZn(Ph)(OtBu)_2]_2$ **3b** in d_8-THF.

<u>Synthesis of $[K_2Zn(Ph)_2(OtBu)_2]$ (**Ib**)</u>

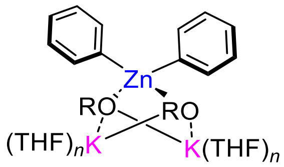

In an argon flushed Schlenk flask, $Zn(TMP)_2$ (1 mmol, 350 mg) and KO*t*Bu (2 mmol, 220 mg) were suspended in benzene (5 mL) at room temperature and left to stir for 24h. All solvent removed under vacuum and the resulting white solid washed with hexane (5 x 10 mL). All solvent removed under vacuum to afford $K_2Zn(Ph)_2(OtBu)_2$ **Ib** (400 mg, 91%). **^{1}H-NMR (300.1 MHz, D_8-THF, 298 K):** δ / ppm = 7.84 (br. m, 4H, 4 x Ar-H), 6.97 (br. m, 4H, 4 x Ar-H), 6.84 (br. m, 2H, 2 x Ar-H), 1.13 (s, 18H, CH_3, 2 x O*t*Bu). **$^{13}C\{^1H\}$-NMR (101 MHz, D_8-THF, 298 K):** δ / ppm = 172.0 (s, 2 x C_q-Zn), 140.9 (s, 2 x C-H), 126.0 (s, 2 x C-H), 123.4 (s, 2 x C-H), 36.2 (s, CH_3, 2 x O*t*Bu). C_q carbon signal for O*t*Bu moiety under THF solvent peak. Large singlet peak at 7.31 ppm correlates to free benzene (1/3 of a molecule relative to **Ib**) which could not be removed under vacuum (possibly due to coordination to the K atom and then liberated once in d_8-THF).

^{1}H-DOSY NMR spectroscopic studies indicate the formation of a single molecular potassium zincate entity $(THF)_2K_2Zn(Ph)_2(OtBu)_2$ **Ib** based on the independent diffusion coefficients of the chemical shifts in the ^{1}H NMR spectra representing the aryl moiety and the alkoxide fragment.

$(THF)_nK_2Zn(Ph)_2(OtBu)_2$ (**Ib**): $D = 6.1\times10^{-10}$ m^2s^{-1}

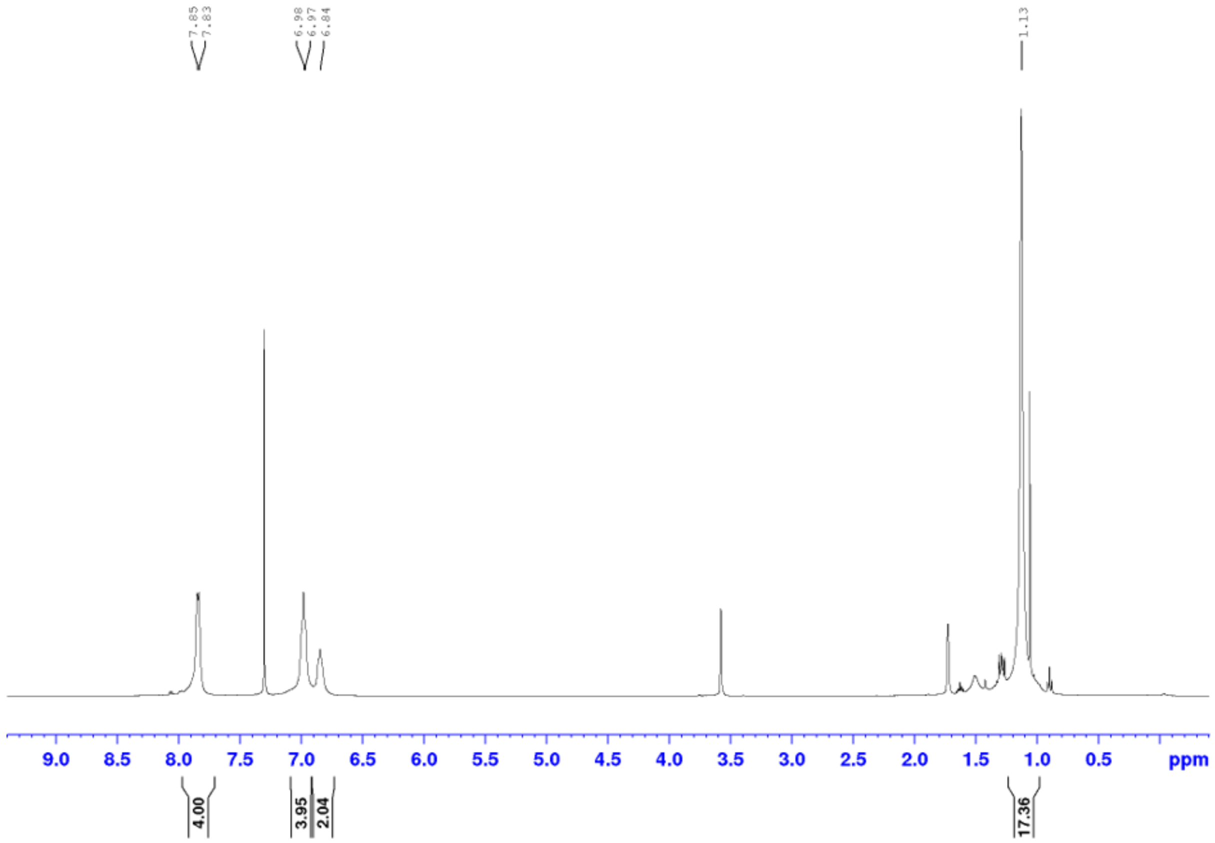

Figure S 34 ^{1}H-NMR spectra of $K_2Zn(Ph)_2(O\textit{t}Bu)_2$ **Ib** in d_8-THF.

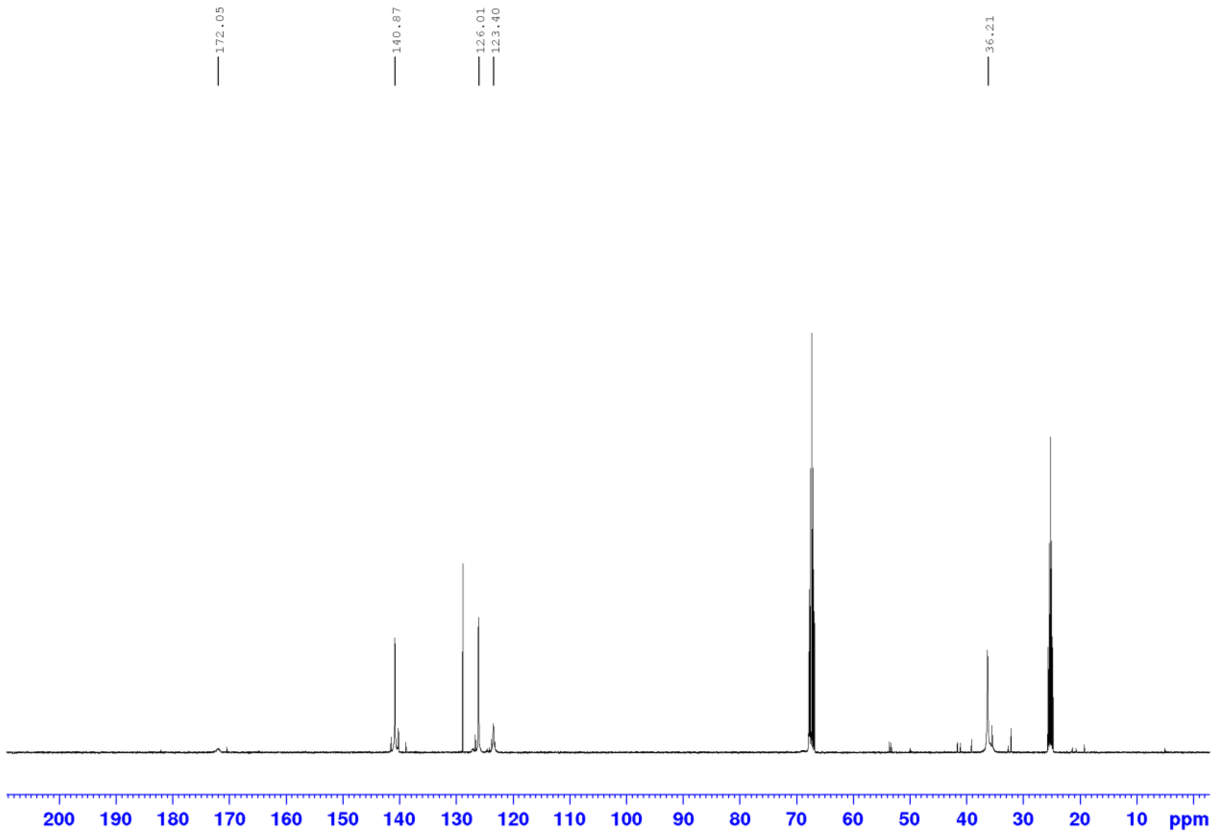

Figure S 35 ^{13}C{^{1}H}-DOSY NMR spectra of $K_2Zn(Ph)_2(O\textit{t}Bu)_2$ **Ib** in d_8-THF.

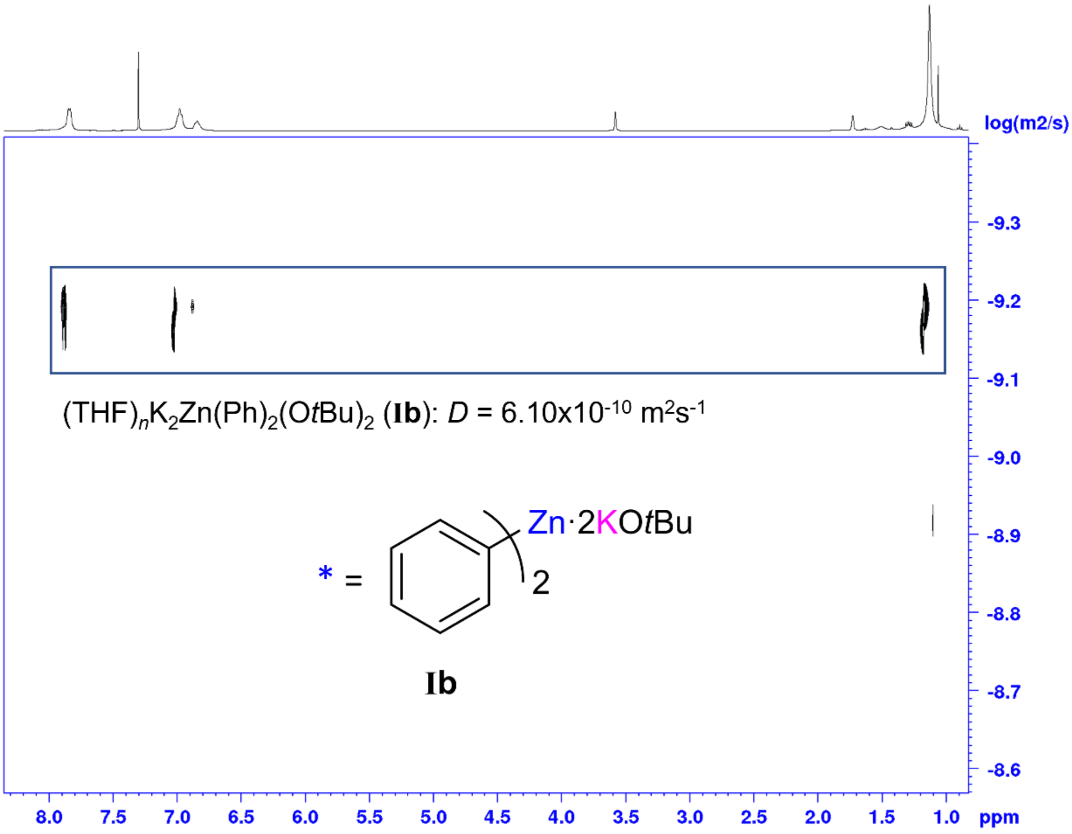

Figure S 36 ^{1}H-DOSY NMR spectra of $K_2Zn(Ph)_2(OtBu)_2$ **Ib** in d_8-THF.

Synthesis of $[(THF)_2KZn(2\text{-benzoxazolyl})(O\textit{t}Bu)_2]_2$ (3c)

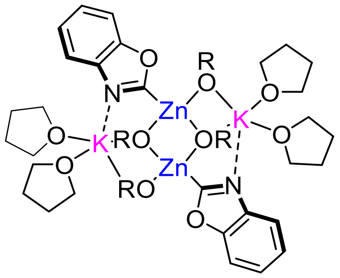

To an argon flushed Schlenk flask, $Zn(TMP)_2$ (350 mg, 1 mmol), KO*t*Bu (220 mg, 2 mmol) and 1,3-benzoxazole (240 mg, 2 mmol) were dissolved in THF (5 mL) giving a red solution. After 20 mins stirring at room temperature solvent half volume under vacuum and hexane (2 mL) layered on top of the THF solution. The solution was then stored at -30 ˚C overnight affording crop of colourless crystals – compound **3c**. Yield 250 mg, 24%. Anal. Calcd. for $C_{34}H_{52}N_2K_2O_7Zn_2$ (1 THF molecule coordinated) C, 50.43; H, 6.47. Found: C, 49.66; H, 6.49. **^{1}H-NMR (300.1 MHz, D_8-THF, 298 K):** δ / ppm = 7.49 (m, 2H, Ar-H), 7.10 (m, 2H, Ar-H), 3.61 (THF), 1.76 (THF), 1.30 (2 x s overlapping, s, 18H, 2 x O*t*Bu). **^{13}C{^{1}H}-NMR (101 MHz, D_8-THF, 298 K):** δ / ppm = 197.2 (s, C_q-Zn), 152.3 (s, C_q), 142.3 (s, C_q), 122.1 (s, Ar, C-H), 121.5 (s, Ar, C-H), 117.7 (s, Ar, C-H), 109.6 (s, Ar, C-H), 69.3 (s, C_q, O*t*Bu), 68.1 (s, C_q, O*t*Bu), 67.2 (THF), 34.8 (s, CH_3, O*t*Bu), 33.4 (s, CH_3, O*t*Bu), 25.4 (THF).

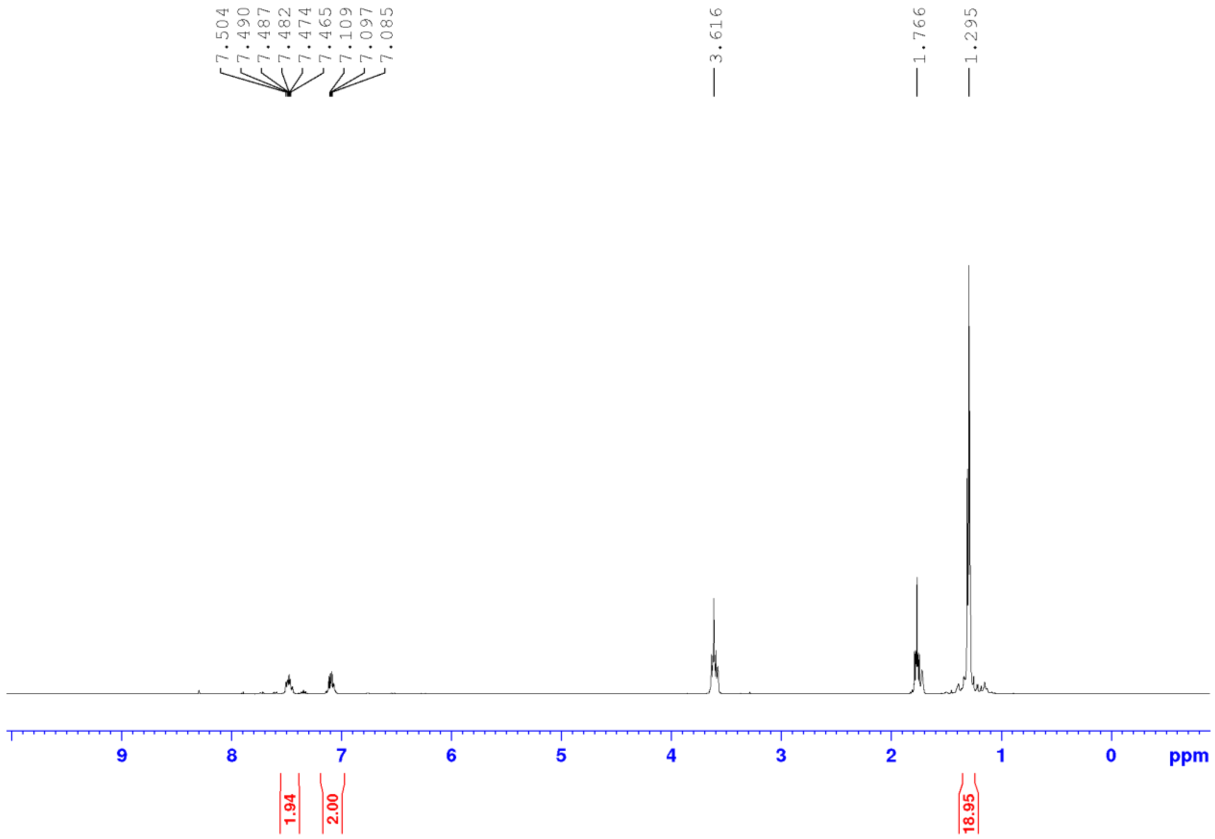

Figure S 37 ^{1}H-NMR spectra of [(THF)$_2$KZn(2-benzoxazolyl)(O*t*Bu)$_2$]$_2$ **3c** in d_8-THF.

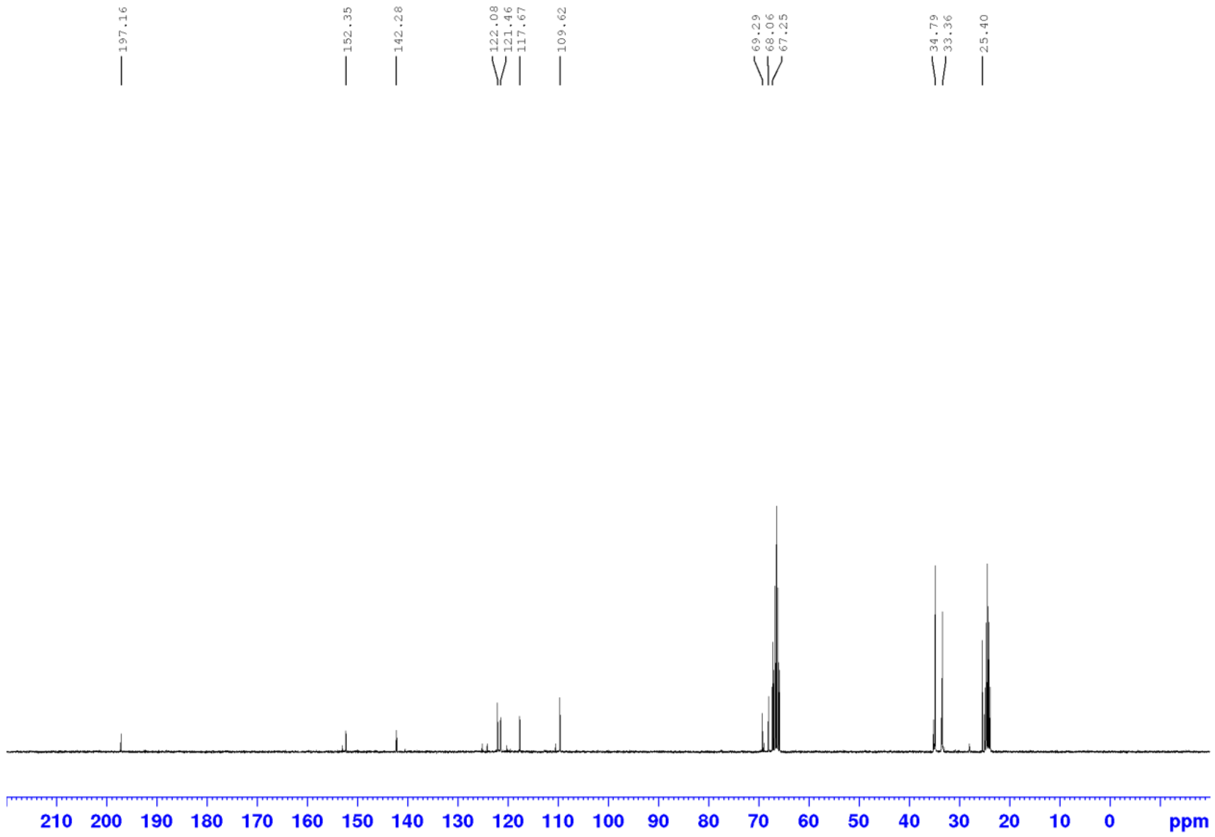

Figure S 38 $^{13}C\{^{1}H\}$ NMR spectra of [(THF)$_2$KZn(2-benzoxazolyl)(O*t*Bu)$_2$]$_2$ **3c** in d_8-THF.

Synthesis of potassium isocyanophenolate [K(1,2-O-C_6H_4-NC)]$_\infty$ (**4**)

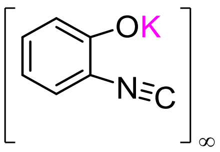

To an argon flushed Schlenk flask, 1,3-benzoxazole (0.5 mmol, 60 mg) was dissolved in benzene (2.5 mL) and cooled to 0 °C. K(CH_2SiMe_3) (0.5 mmol, 63mg) was then added via a solid addition tube giving a red/brownish suspension. After 10 mins, all solvent was removed, and the resulting solid was dried under vacuum affording a red/brown solid – compound **4**. Yield 75 mg, 48%. **^{1}H-NMR (300.1 MHz, D_8-THF, 298 K):** δ / ppm = 6.94 (dd, J = 7.70 Hz, 1.85 Hz, 1H, Ar-H), 6.85 (td, J = 7.76 Hz, 1.91, 1H, Ar-H), 6.50 (d, J = 8.30 Hz, 1H, Ar-H), 5.98 (td, J = 7.54 Hz, 1.15 Hz, 1H, Ar-H). **^{13}C{^{1}H}-NMR (101 MHz, D_8-THF, 298 K):** δ / ppm = 168.8 (s, Ar, C_q), 163.8 (s, Ar, C_q), 130.7 (s, Ar, C-H), 128.8 (s, Ar, C-H), 121.3 (s, Ar, C-H), 117.4 (s, *C*N), 108.5 (s, Ar, C-H). NMR spectroscopy fits well with those reported for lithium 2-isocyanophenolate.[17]

Note: Taking a portion of this isolated solid and quenching with iodine affords the quantitative conversion to 2-iodobenzoxazole **2i**.

^{1}H-DOSY NMR of **4** in d_8-THF displayed a diffusion coefficient (*D*) of 6.15 x10^{-10} m^2s^{-1}. Using Stalke's external calibration curve (ECC) method for molecular weight determinations of organometallic species in solution, the molecular weight determined, MW_{det}, for **4** was 676 g/mol. The calculated molecular weight, MW_{calc}, of **4** existing in d_8-THF as a tetrasolvated dimer was 637 g/mol giving an error of -6% for this particular aggregate using tetraphenylnaphthalene (D = 7.41 x10^{-10} m^2s^{-1}) as an internal standard (Figure S41). [18] This is in good agreement with our previous ^{1}H-DOSY studies for KO*t*Bu which was also predicted to exist as a tetrasolvated dimer in d_8-THF solution.[19]

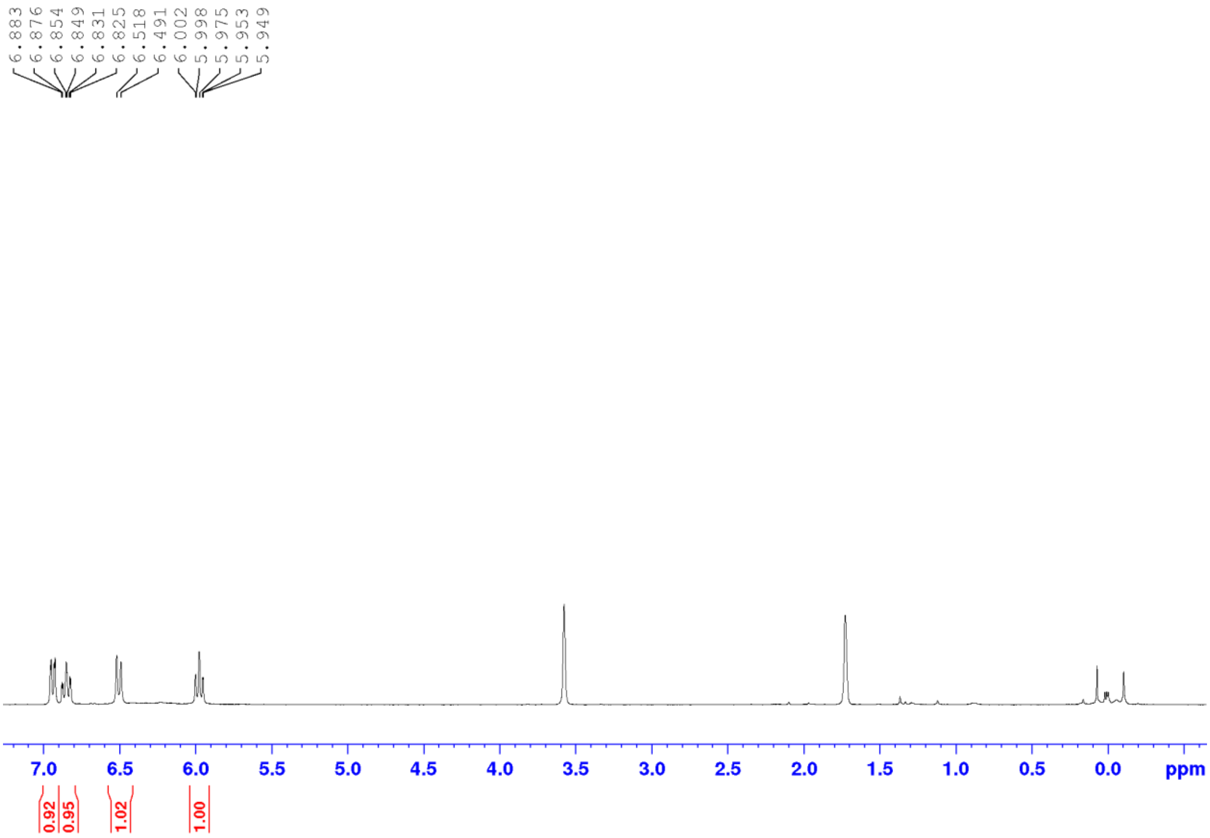

Figure S 39 ^{1}H-NMR spectra of [(THF)$_2$K(1,2-O-C$_6$H$_4$-NC)]$_2$ **4** in d$_8$-THF.

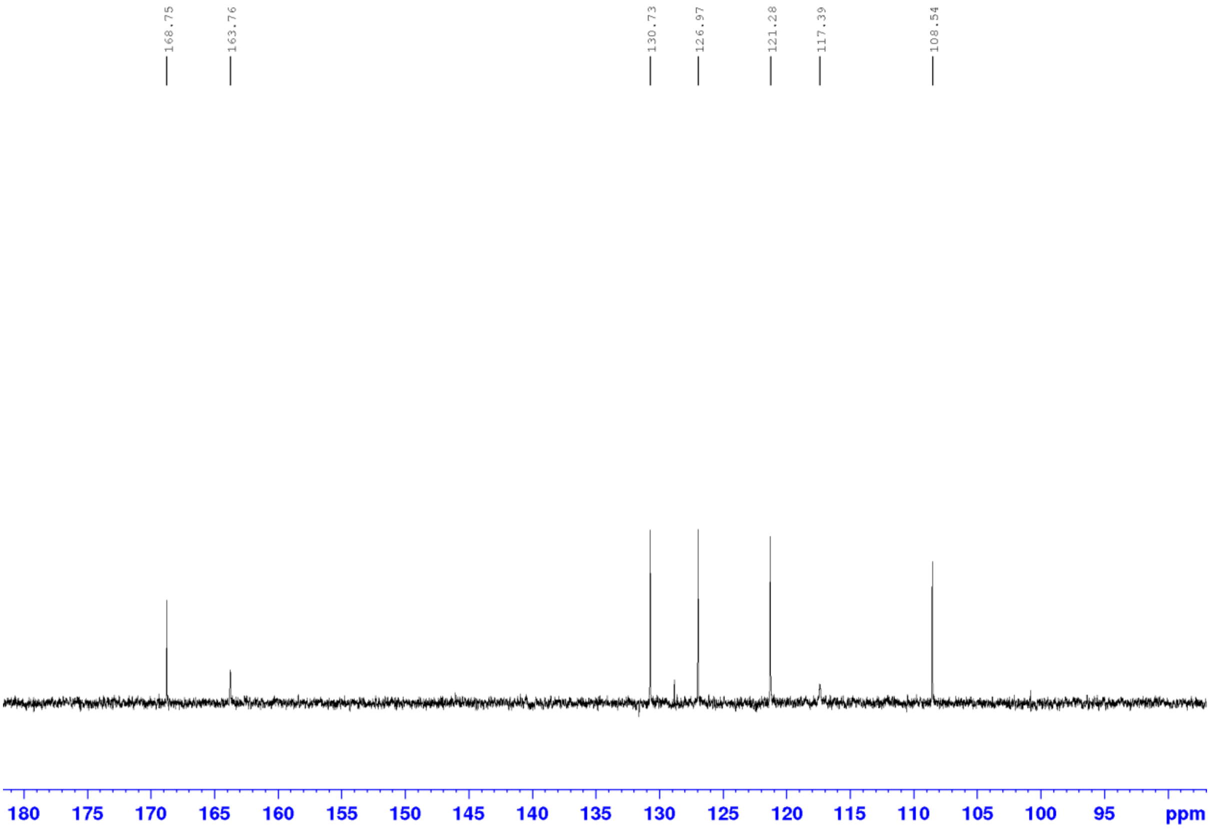

Figure S 40 ^{13}C{^{1}H} NMR spectra of [(THF)$_2$K(1,2-O-C$_6$H$_4$-NC)]$_2$ **4** in d$_8$-THF.

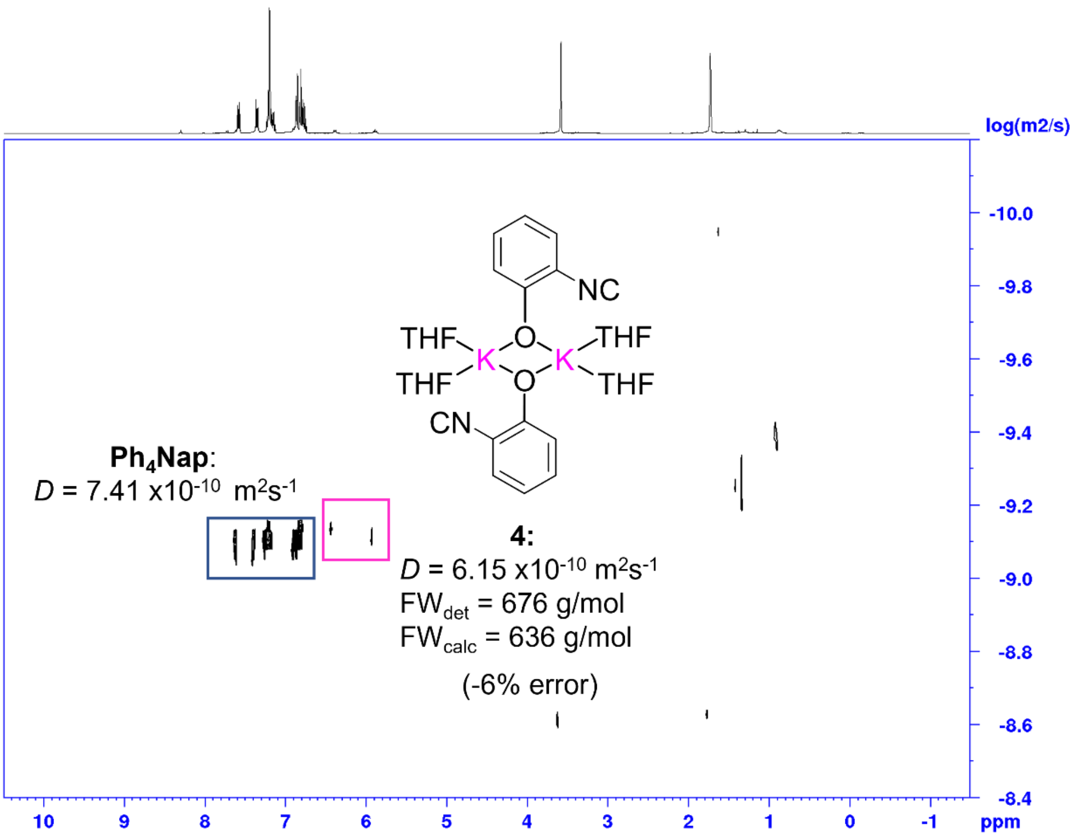

Figure S 41 ^{1}H-DOSY NMR spectra of $[(THF)_2K(1,2\text{-}O\text{-}C_6H_4\text{-}NC)]_2$ **4** in d_8-THF.

Synthesis of $[(THF)_2K_2Zn(CH_2\text{-}3,5\text{-}Me_2\text{-}C_6H_3)_2(OtBu)_2]_\infty$ (**5**)

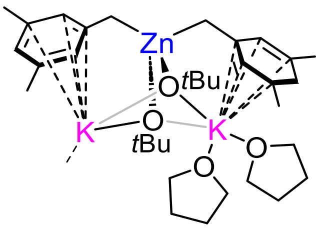

To an argon flushed Schlenk flask, $Zn(TMP)_2$ (180 mg, 0.5 mmol) and KO*t*Bu (110 mg, 0.5 mmol) were suspended in mesitylene (5 mL) and allowed to stir overnight, forming an orange suspension. The solvent was then removed *in-vacuo* and the resulting solid re-suspended in hexane (10 mL). Solid washed with hexane (3 x 10 mL) to remove excess mesitylene and then dried under vacuum to give a white solid – compound **5**. Yield 170 mg, 64%. A portion of this solid was suspended in hexane in a vial in the glovebox and THF was dripped into the suspension until an orange solution was obtained. Storing this solution at -30 °C afforded a crop of crystals which were then measured by X-ray crystallography. Anal. Calcd. for $C_{26}C_{40}K_2O_2Zn$ (0 THF molecule coordinated – THF removed under vacuum) C, 59.12; H, 7.63. Found: C, 58.68; H, 7.47. **^{1}H-NMR (300.1 MHz, D_8-THF, 298 K):** δ / ppm = 6.56 (s, 4H, 4 x Ar-H), 5.96 (s, 2H, 2 x Ar-H), 3.61 (THF), 2.06 (s, 12H, 4 x CH_3), 1.77 (THF), 1.59 (s, 4H, 2 x Zn-CH_2), 1.15 (s, 18H, 2 x O*t*Bu). **^{13}C{^{1}H}-NMR (101 MHz, D_8-THF, 298 K):** δ / ppm = 159.1 (s, Ar, C_q), 137.9 (s, Ar, *C*-H), 122.1 (s, Ar, *C*-H), 116.9 (s, Ar, C_q), 68.0 (THF), 35.0 (s, CH_3, 2 x O*t*Bu), 30.5 (s, 2 x *C*H_2-Zn), 26.2 (THF), 21.5 (s, 4 x CH_3).

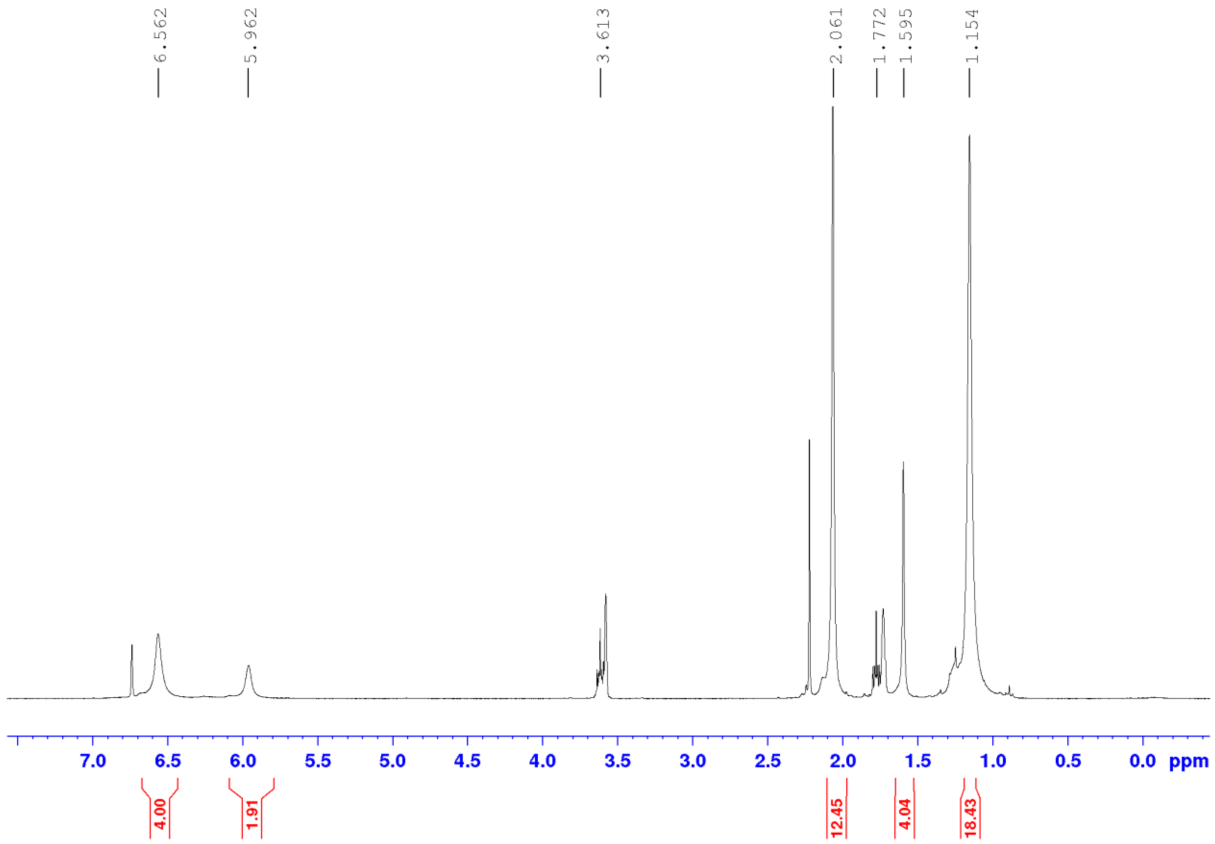

Figure S 42 ^{1}H-NMR spectra of $[(THF)_2K_2Zn(CH_2\text{-}3,5\text{-}Me_2\text{-}C_6H_3)_2(O\textit{t}Bu)_2]_\infty$ in D_8-THF.

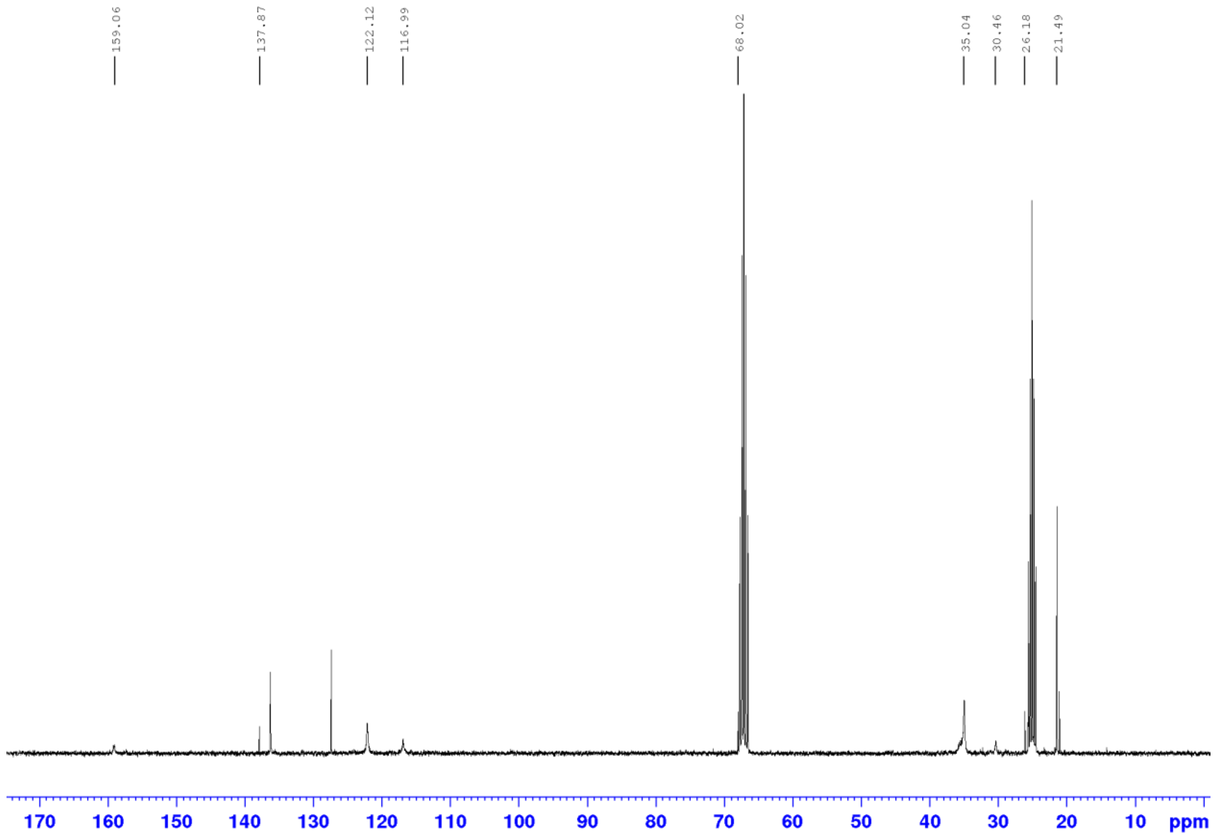

Figure S 43 ^{13}C{^{1}H} NMR spectra of $[(THF)_2K_2Zn(CH_2\text{-}3,5\text{-}Me_2\text{-}C_6H_3)_2(O\textit{t}Bu)_2]_\infty$ in D_8-THF.

Synthesis of compound [(PMDETA)$_2$KZn(C_4H_5)(O*t*Bu)$_2$]$_2$ (**7**)

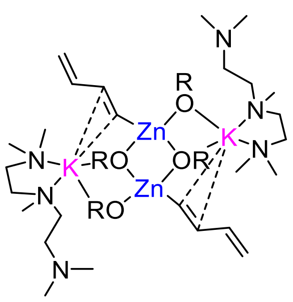

To an argon flushed Schlenk flask, Zn(TMP)$_2$ (350 mg, 1 mmol) and KO*t*Bu (220 mg, 2 mmol) were allowed to stir in THF (5 mL) for 3 days at room temperature giving a deep purple/black solution. All solvent removed and the resulting oil was reconstituted in hexane (5 mL) giving a wispy suspension. PMDETA (0.42 mL, 2 mmol) was then added to give a pale-yellow solution. The solution was then stored at -30 °C overnight affording crop of colourless crystals – compound **7**. Yield 350 mg, 37%. **^{1}H-NMR (300.1 MHz, D$_8$-THF, 298 K):** δ / ppm = 6.68 (2 x overlapping m, 2H), 6.23 (m, 2H), 4.73 (dd, J = 17.08 Hz, 2.27 Hz, 1H), 4.54 (dd, J = 10 Hz, 2.5 Hz, 1H), 2.35 (m, 8H, 2 x (CH_2)$_2$, PMDETA), 2.18 (s, 3H, NMe, PMDETA), 2.14 (s, 12H, 2 x NMe$_2$, PMDETA), 12.5 (s, 9H, O*t*Bu), 1.15 (s, 9H, O*t*Bu). **^{13}C{^{1}H}-NMR (101 MHz, D$_8$-THF, 298 K):** δ / ppm = 164.2 (s, Zn-*C*=CH), 146.3 (s, *C*H=CH), 143.7 (s, CH_2), 107.5 (s, *C*H=CH), 58.7 (s, CH_2-PMDETA), 57.3 (s, CH_2-PMDETA), 46.0 (s, CH_3-PMDETA), 46.0 (s, CH_3-PMDETA), 35.4 (s, CH_3, O*t*Bu), 33.8 (s, CH_3, O*t*Bu).

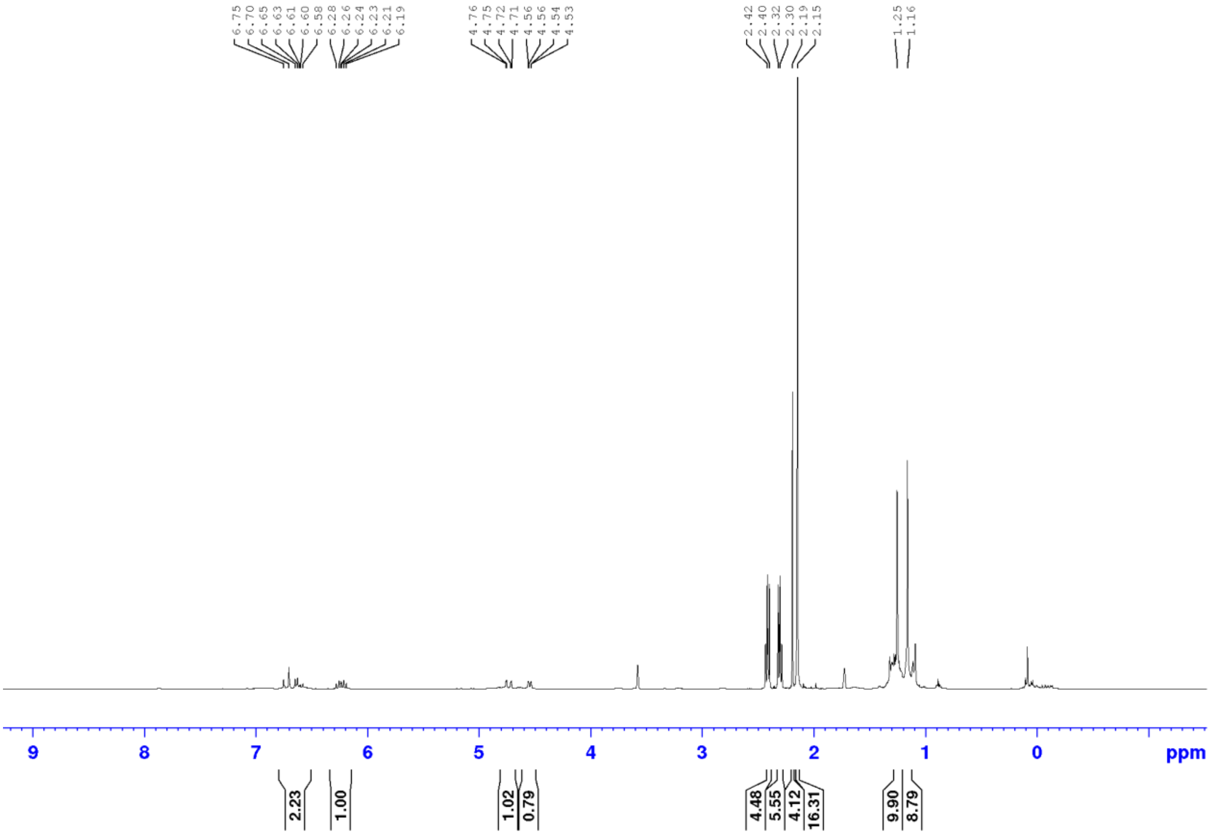

Figure S 44 ^{1}H-NMR spectra [(PMDETA)$_2$KZn(C_4H_5)(O*t*Bu)$_2$]$_2$ (**7**) in d$_8$-THF.

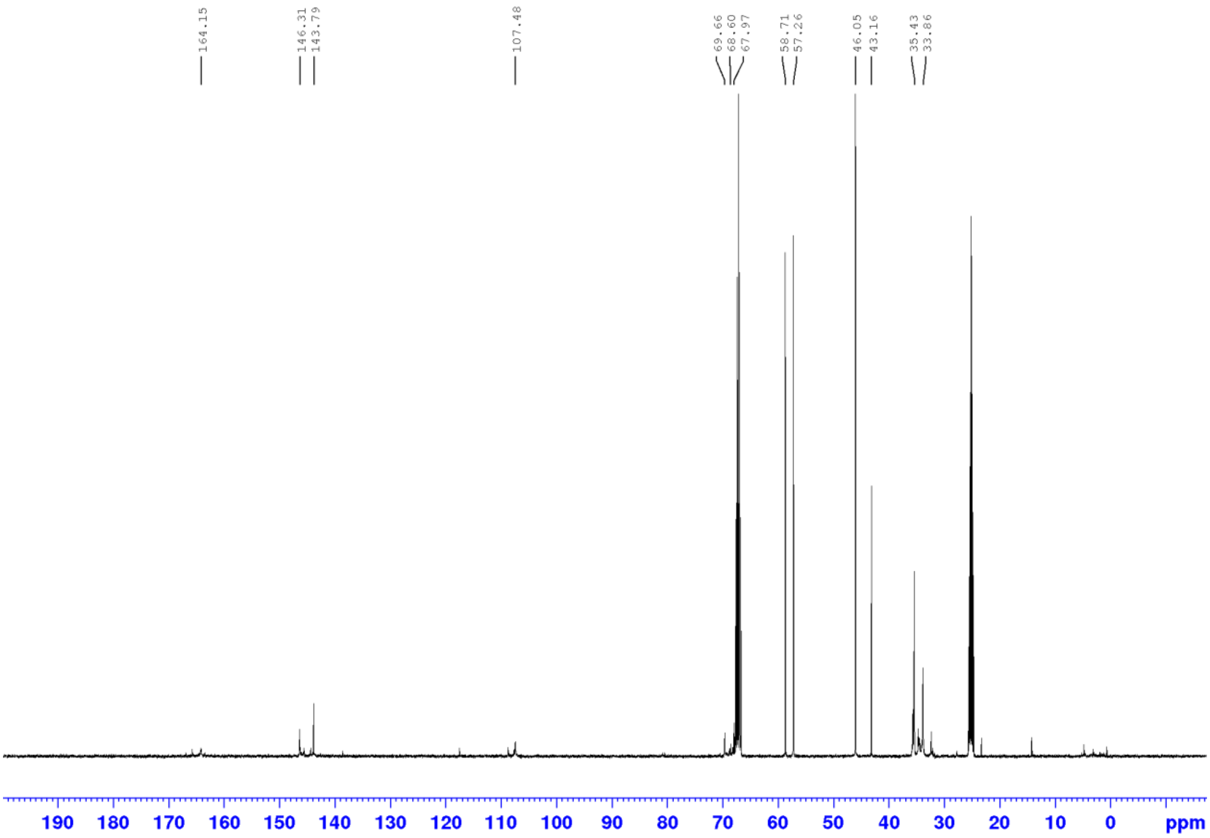

Figure S 45 $^{13}C\{^1H\}$-NMR spectra [(PMDETA)KZn(C_4H_5)(O*t*Bu)$_2$]$_2$ (**7**) in d_8-THF.

3.5 Synthesis of acetophenones **6a** and **6b**

Synthesis of 2-(3,5-dimethylphenyl)-1-phenylethan-1-one **6a**

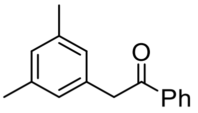

In an argon flushed Schlenk flask, Zn(TMP)$_2$ (0.5 mmol, 180 mg) and KO*t*Bu (1 mmol, 110 mg) were suspended in mesitylene (5 mL) at room temperature and left to stir for 24h at room temperature resulting in an orange suspension. All solvent removed under vacuum and the resulting solid dissolved in THF (5 mL). N-Methoxy-N-methylbenzamide (1 mmol, 0.15 mL) was subsequently added via syringe immediately forming a colourless solution which was stirred at ambient temperature for 2 hours. The reaction was quenched with H_2O and extracted from aq. NH_4Cl with EtOAc. The organics were collected, dried over $MgSO_4$ and concentrated in vacuo. Purified via silica column chromatography (Hexane:EtOAc 100:0 to 80:20) to produce **6a** as a colourless oil (171 mg, 76%). **^{1}H-NMR (300.1 MHz, CDCl$_3$, 298 K):** δ / ppm = 8.03-8.00 (m, 2H, Ar-H), 7.55 (tt, J = 7.3 Hz, 2.1 Hz, 1H, Ar-H), 7.46 (tt, J = 7.6 Hz, 1.6 Hz, 2H, Ar-H), 6.89 (s, 3H, Ar-H), 4.20 (s, 2H, C*H*$_2$), 2.29 (s, 3H, CH_3 x 2). **$^{13}C\{^1H\}$-NMR (101 MHz, CDCl$_3$, 298 K):** δ / ppm = 198.0 (s, C_q, *C*=O), 138.3 (s, 2 x *C*-H, Ar), 136.8 (s, C_q, Ar), 134.4 (s, 2 x C_q, Ar), 133.2 (s, *C*-H, Ar), 128.8 (s, 2 x *C*-H, Ar), 128.7 (s, 2 x *C*-H, Ar), 127.3 (s, 2 x *C*-H, Ar), 45.5 (s, *C*H$_2$, Ph*C*H$_2$), 21.2 (s, 2 x *C*H$_3$). NMR spectroscopic data consistent with literature reports.[20]

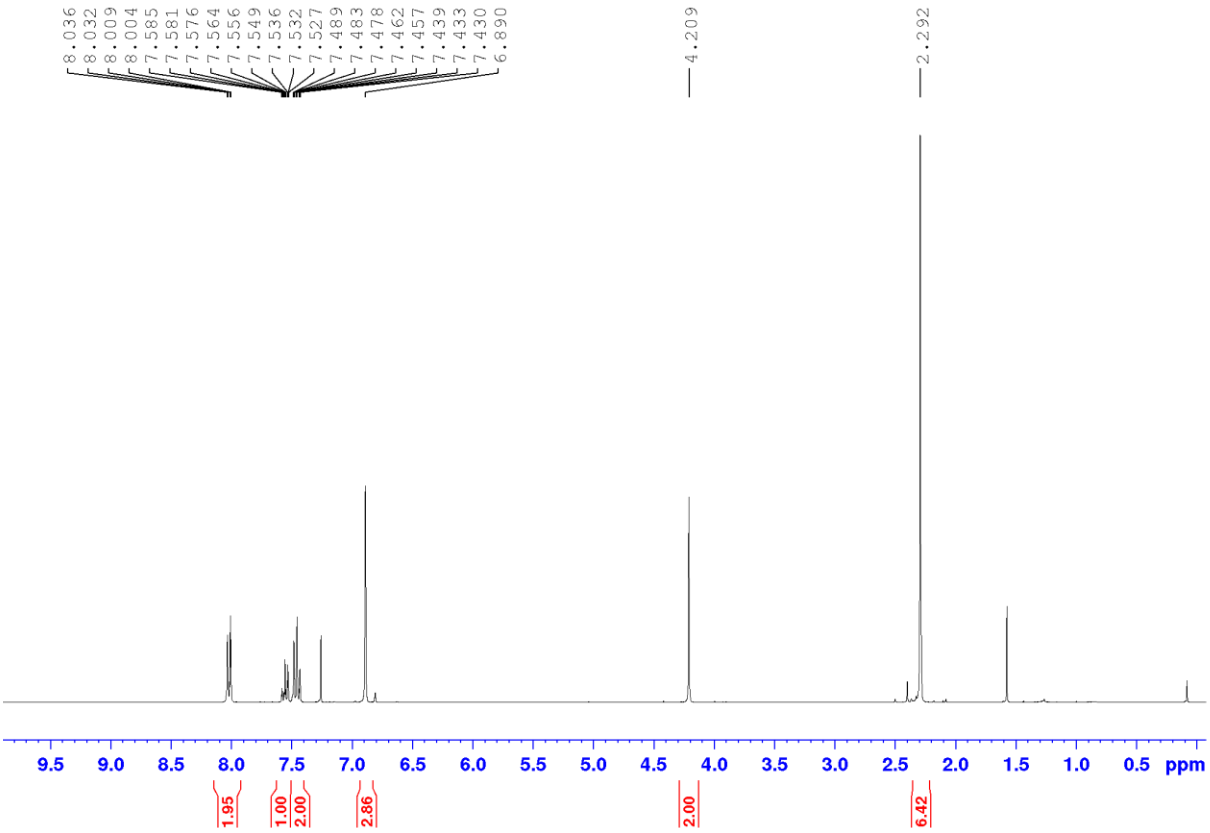

Figure S 46 ^{1}H-NMR spectra of 2-(3,5-dimethylphenyl)-1-phenylethan-1-one **6a** in $CDCl_3$.

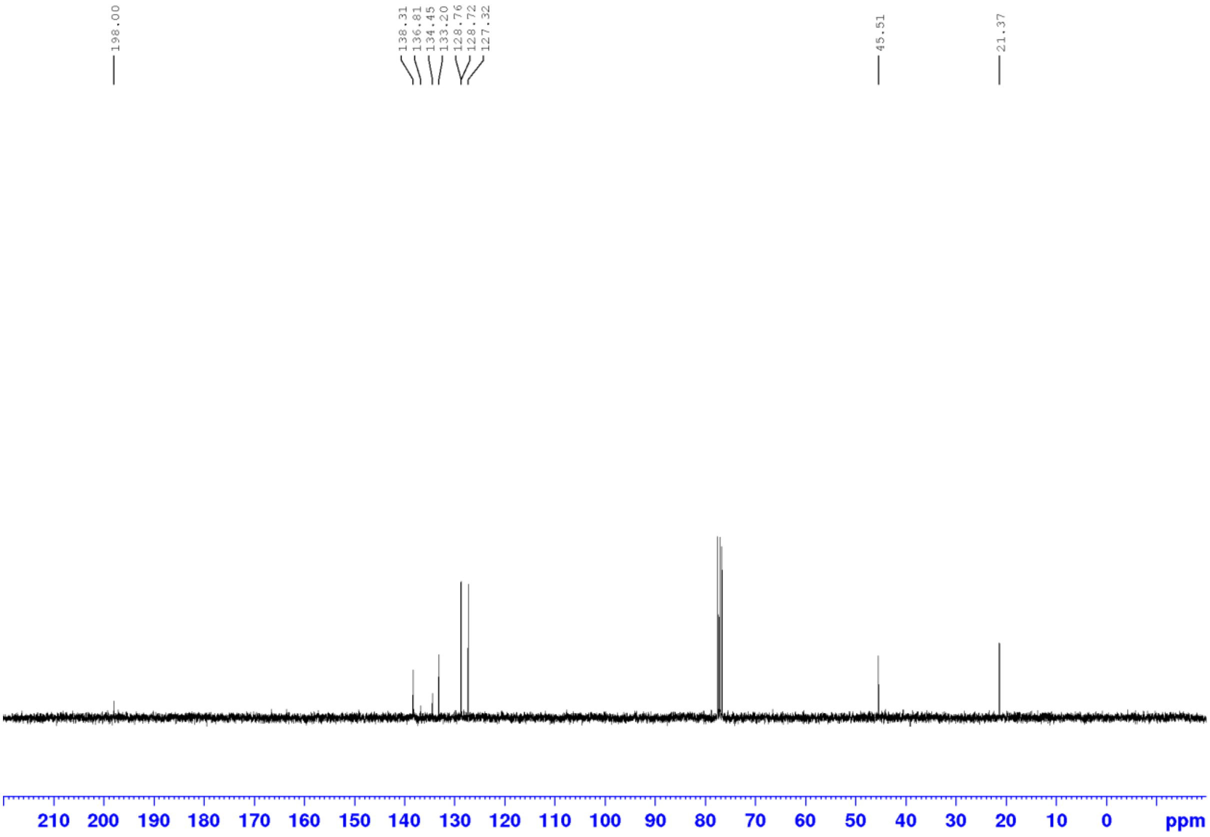

Figure S 47 ^{13}C{^{1}H}-NMR spectra of 2-(3,5-dimethylphenyl)-1-phenylethan-1-one **6a** in $CDCl_3$.

Synthesis of 2-(*m*-tolyl)phenylethan-1-one **6b**

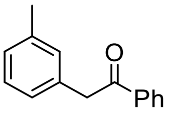

In an argon flushed Schlenk flask, $Zn(TMP)_2$ (0.5 mmol, 180 mg) and KO*t*Bu (1 mmol, 110 mg) were suspended in mesitylene (5 mL) at room temperature and left to stir for 24h at room temperature resulting in an orange suspension. All solvent removed under vacuum and the resulting solid dissolved in THF (5 mL). N-Methoxy-N-methylbenzamide (1 mmol, 0.15 mL) was subsequently added via syringe immediately forming a colourless solution which was stirred at ambient temperature for 2 hours. The reaction was quenched with H_2O and extracted from aq. NH_4Cl with EtOAc. The organics were collected, dried over $MgSO_4$ and concentrated in vacuo. Purified via silica column chromatography (Hexane:EtOAc 100:0 to 95:5) to produce **6b** as a colourless oil (150 mg, 71%).**^{1}H-NMR (300.1 MHz, $CDCl_3$, 298 K):** δ / ppm = 8.04-8.01 (m, 2H, Ar-H), 7.59-7.53 (m, 1H, Ar-H), 7.49-7.44 (m, 2H, Ar-H), 7.26-7.20 (m, 1H, Ar-H), 7.09-7.06 (br.m, 3H, Ar-H), 4.26 (s, 2H, PhC*H*$_2$), 2.34 (s, 3H, CH_3). **^{13}C{^{1}H}-NMR (101 MHz, $CDCl_3$, 298 K):** δ / ppm = 197.8 (s, C_q, *C*=O), 138.4 (s, C_q, Ar), 136.8 (s, C_q, Ar), 133.2 (s, C_q, Ar), 134.5 (s, C_q, Ar), 133.2 (s, *C*-H, Ar), 130.3 (s, *C*-H, Ar), 128.7 (s, 4 x *C*-H, Ar), 128.6 (s, *C*-H, Ar), 127.8 (s, *C*-H, Ar), 126.6 (s, *C*-H, Ar), 45.5 (s, *C*H$_2$, PhCH$_2$), 21.5 (s, CH_3). NMR spectroscopic data consistent with literature reports.[21]

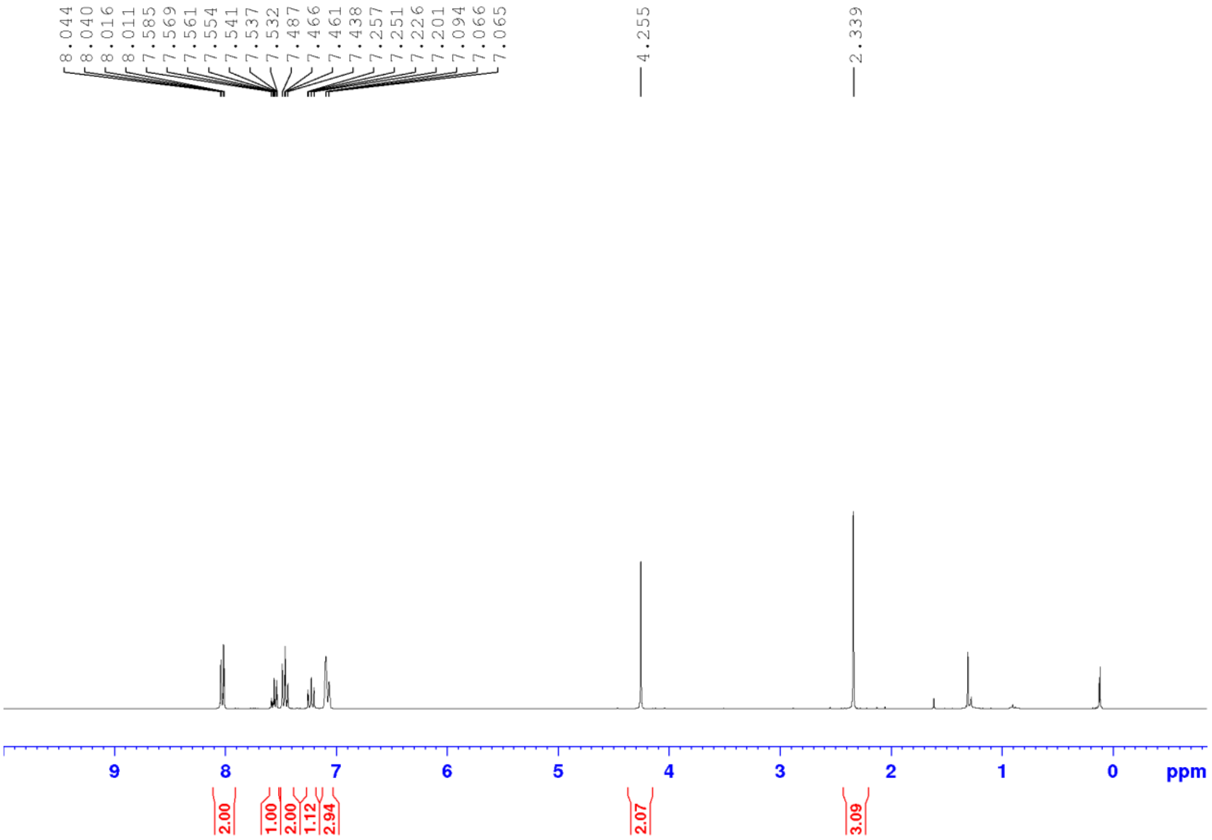

Figure S 48 ^{1}H-NMR spectra 2-(*m*-tolyl)phenylethan-1-one **6b** in $CDCl_3$.

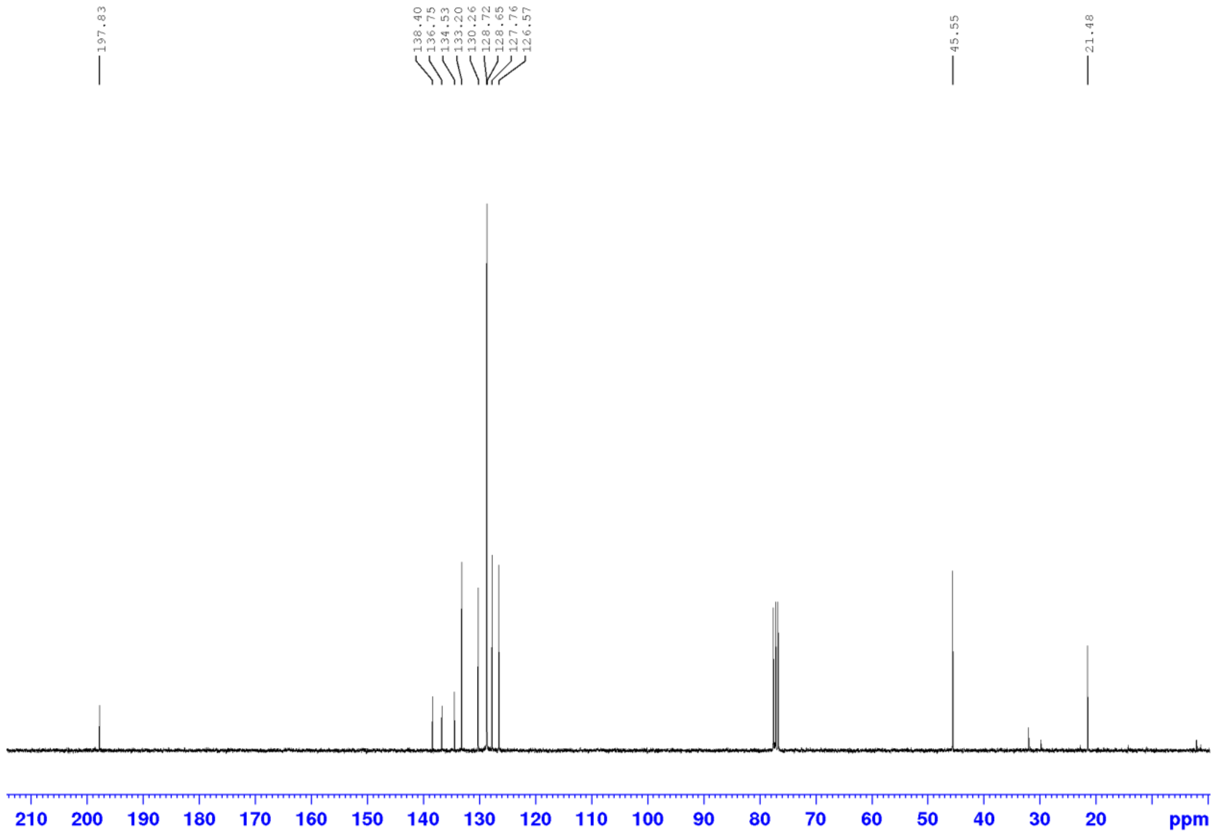

Figure S 49 $^{13}C\{^{1}H\}$ NMR spectra 2-(*m*-tolyl)phenylethan-1-one **6b** in $CDCl_3$.

4. NMR Reaction Monitoring

4.1 Zincation and iodination of Naphthalene using Zn(TMP)$_2$/2KO*t*Bu

***In-situ* iodination**:

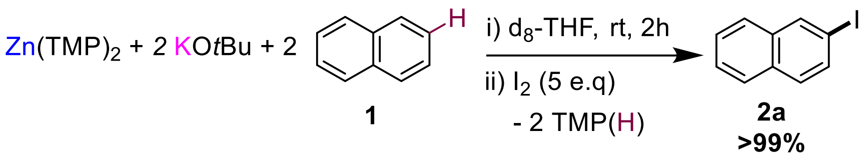

In an argon flushed J Young's NMR tube, naphthalene (0.2 mmol, 26 mg) and hexamethylbenzene (approx. 5.4 mg) were dissolved in d_8-THF (0.5 mL). ^{1}H NMR recorded. To this solution was added Zn(TMP)$_2$ (0.1 mmol, 35 mg) and KO*t*Bu (0.2 mmol, 22 mg). ^{1}H NMR monitoring showed full conversion of naphthalene after 2h at room temperature. Sublimed iodine (128 mg, 0.5 mmol) was then added to this solution giving a >95% yield of 2-iodonaphthalene **2a** after 1h (yield determined using hexamethylbenzene as internal standard).

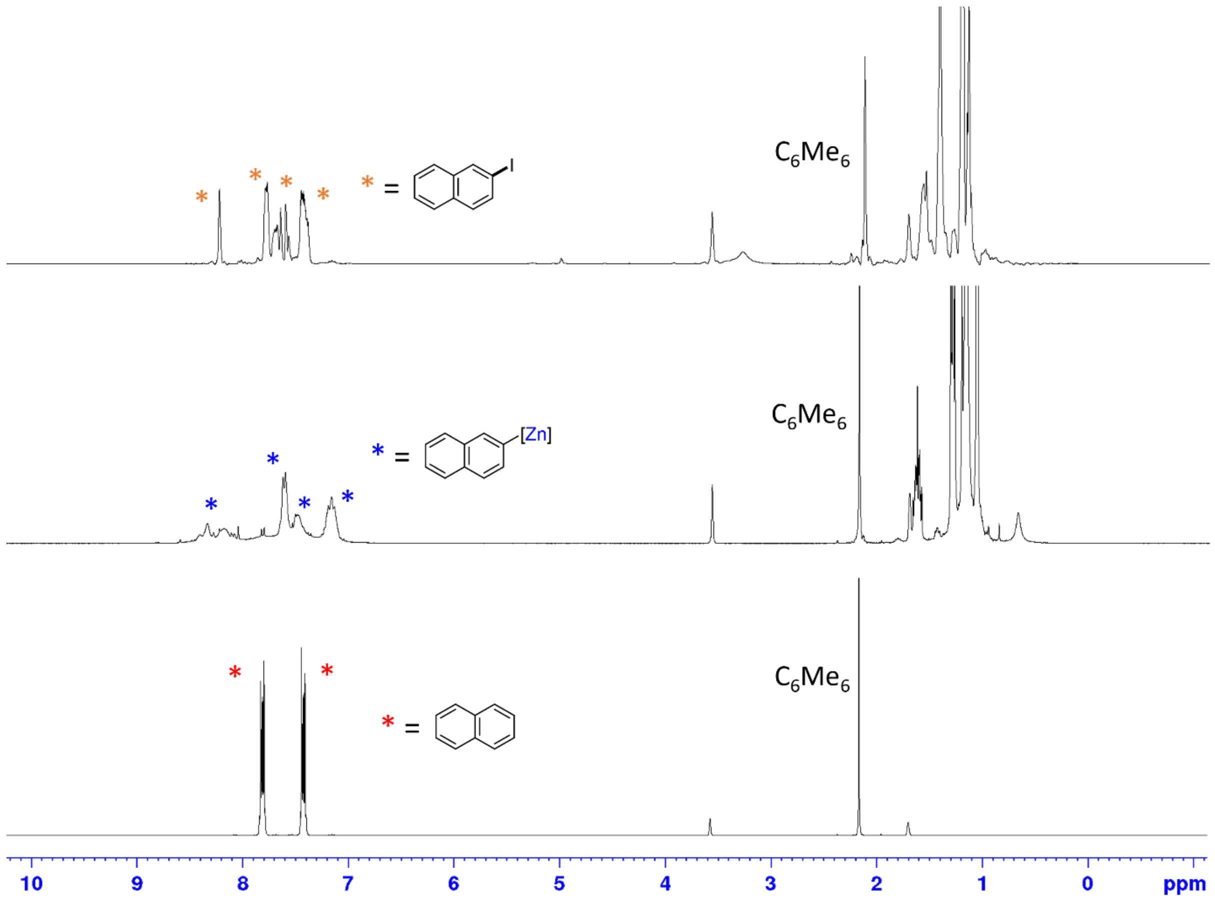

Figure S 50 Stacked ^{1}H NMR spectra of naphthalene zincation with $Zn(TMP)_2$/2KO*t*Bu. (Bottom) t = 0, two equivalents of naphthalene and hexamethylbenzene internal standard. (Middle) t = 2h after addition of $Zn(TMP)_2$/2KO*t*Bu demonstrating complete consumption of naphthalene starting material and emergence of C2-zincated naphthalene signals (top) 2-iodonaphthalene **2a** in d_8-THF formed after electrophilic quench.

***In-situ* zincation:** Identical procedure to above without the addition of hexamethylbenzene or iodine.

The postulated intermediate of the reaction between two equivalents of naphthalene and $Zn(TMP)_2$/2KO*t*Bu in d_8-THF solution was higher order zincate $[(THF)_nK_2Zn(C2\text{-naphthyl})_2(OtBu)_2]$ (**Ia**) for which its benzene congener (**Ib**) is perfectly stable in d_8-THF and characterised by ^{1}H, ^{13}C and ^{1}H-DOSY NMR (see section 3.4 for details, Figure S34-S36). However, in the case of naphthalene a more complex mixture seems to be present with at least two C2-metalated intermediates. ^{1}H-NMR monitoring shows full conversion of naphthalene after 2 hours at room temperature with the emergence of a set of broad multiplets in the aromatic region (ppm range 8.42 - 7.16), two overlapping singlets at 1.21 and 1.89 ppm in the typical range for the O*t*Bu signals of these species and concomitant formation of two equivalents of TMP(H). The $^{13}C\{^{1}H\}$ spectra of this *in-situ* mixture displays two informative signals at 171.9 and 163.2 ppm in the region for C_q-Zn bonds of metalated arenes. This suggests that two different C2 metalated species are existing in solution. ^{1}H-DOSY NMR proved to be inconclusive and could not aid in elucidating the exact nature of these metalated species due to overlapping signals giving inaccurate diffusion coefficients. Whilst it is possible $[(THF)_nK_2Zn(C2\text{-naphthyl})_2(OtBu)_2]$ (**Ia**) is present in this mixture it cannot be said with certainty. It is a known phenomenon that alkali-metal alkoxides can

induce complex equilibria forming multiple species in solution and we have previously documented this in our study relating to the zincation of fluoroarenes using this mixture.[19,22–24]

Note:

- Compound **3a NOT** detected by NMR spectroscopy in this reaction mixture.
- As demonstrated in Figure S50 and from multiple quenching studies with iodine, this *in-situ* generated mixture quenches with iodine to exclusively furnish 2-iodonaphthalene **2a**. 1-iodonaphthalene never detected.

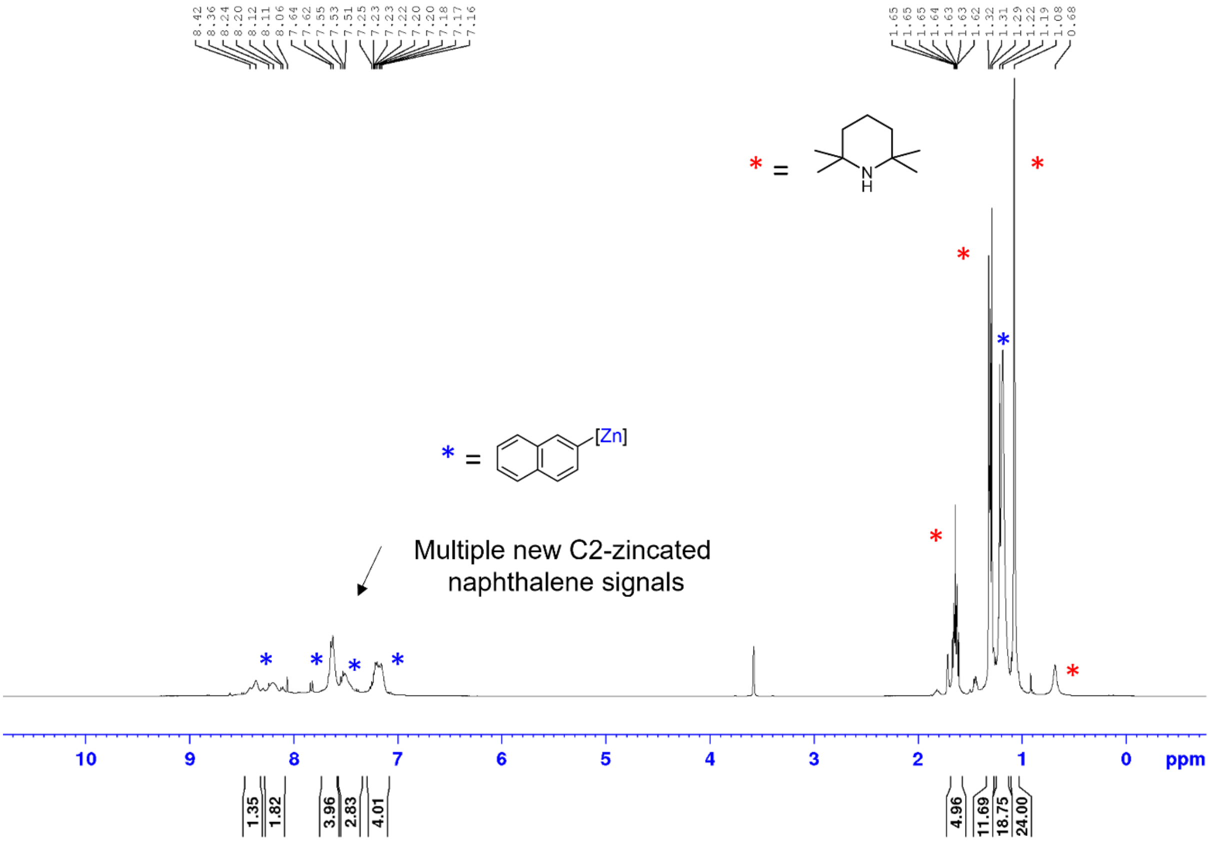

Figure S 51 ^{1}H-NMR spectra of *in-situ* reaction of two equivalents of naphthalene with $Zn(TMP)_2/2KOtBu$ in d_8-THF.

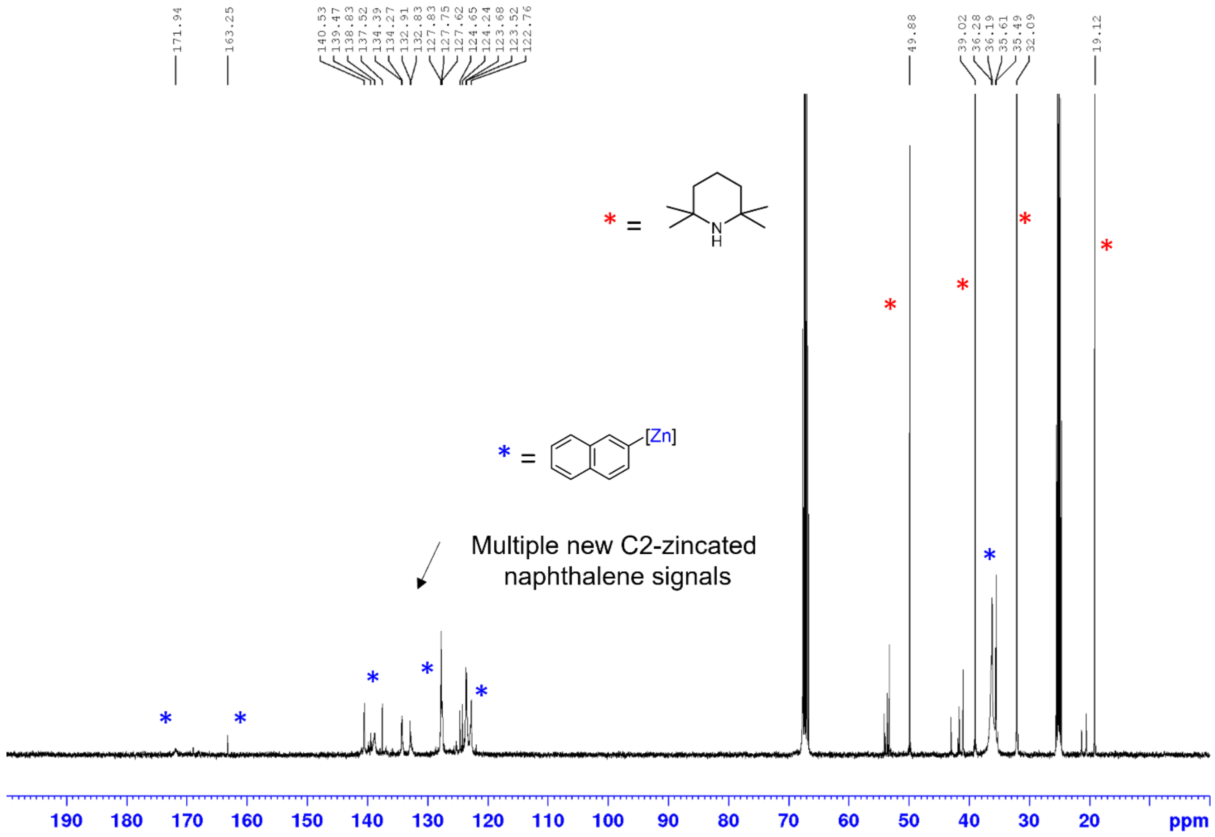

Figure S 52 $^{13}C\{^{1}H\}$ NMR spectra of *in-situ* reaction of two equivalents of naphthalene with $Zn(TMP)_2/2KOtBu$ in d_8-THF.

<u>**4.2** Zincation and iodination of anthracene and possible competing SET process</u>

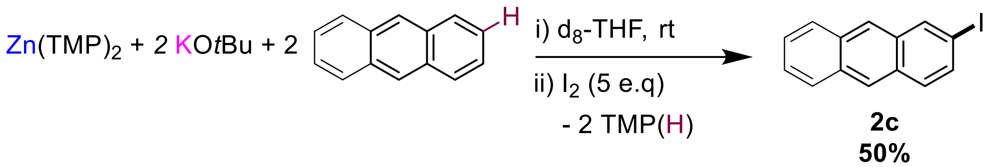

2c
50%
(50% recovery of anthracene)

In an argon flushed Schlenk flask, anthracene (0.5 mmol, 90 mg) was dissolved in THF (2.5 mL). To this solution was added $Zn(TMP)_2$ (0.25 mmol, 90 mg) and KO*t*Bu (0.5 mmol, 55 mg) and left to stir for 2h at room temperature forming a deep orange solution. After the stated time, five equivalents of sublimed I_2 (2.5 mmol, 650 mg) were added and allowed to stir overnight. Reaction mixture quenched with $Na_2S_2O_3$ (10 mL), organics extracted with EtOAc (3 x 10 mL), washed with brine (1 x 10 mL), dried over $MgSO_4$ and filtered. Aliquot taken, all solvent removed, and NMR analysis carried out in $CDCl_3$ using hexamethylbenzene as an internal standard revealing a 1:1 mixture of 2-iodoanthracene **2c** and anthracene starting material (Figure S53).

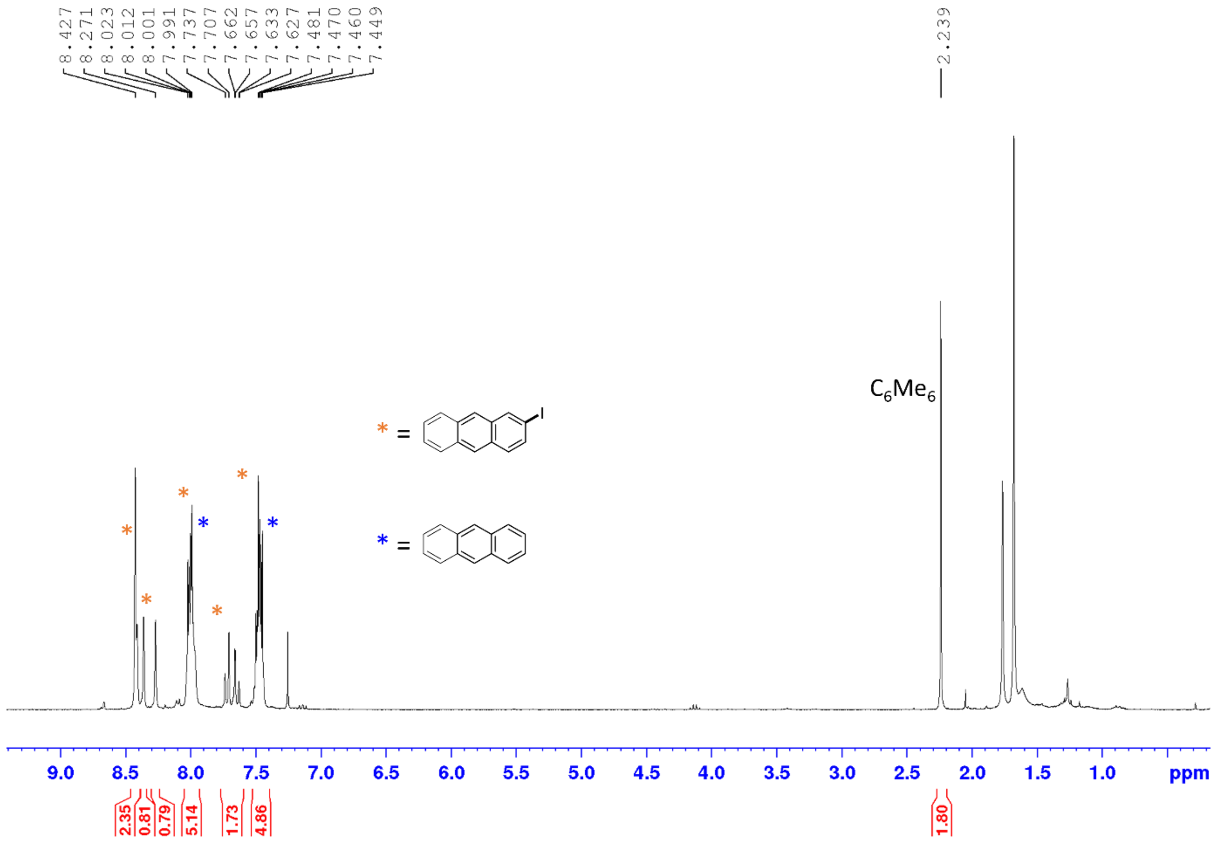

Figure S 53 ^{1}H-NMR spectra in $CDCl_3$ of crude reaction mixture from I_2 quench of reaction between two equivalents of anthracene and $Zn(TMP)_2$/2KO*t*Bu.

***In-situ* iodination:** Carrying out this reaction in d_8-THF in an argon flushed J. Youngs NMR tube, the reaction instantly turns a deep orange colour (and subsequently dark brown overtime) upon addition of the base. ^{1}H-NMR monitoring after 1h displays a completely silent aromatic region and consumption of anthracene (Figure S54b). Overtime signals which have been assigned to zincated anthracene emerge in the aromatic region along with the presence of TMP(H) (indicative of deprotonative metalation) and importantly no anthracene starting material is present at this stage (Figure S54c). Addition of excess I_2 at this stage results in the formation of an approximately 1:1 mixture of 2-iodoanthracene 2c and anthracene starting material (Figure S54d).

We postulate that our $Zn(TMP)_2$/2KO*t*Bu combination is capable of performing single electron transfer (SET) processes and is reducing the anthracene to its paramagnetic radical anion hence the deeply coloured (see Figure S55) NMR silent solution at t = 1h.[25],[26] This perhaps explains the moderate yield of 2-iodoanthracene **2c** as treatment with iodine (which can behave as an oxidant)[27] leads to a 50% recovery of anthracene, suggesting a competing SET process. Compared with naphthalene, anthracene possesses a higher reduction potential presenting a more facile SET process which helps to explain why this competing reaction is not observed for arenes with lower reduction potentials.[28] Although KO*t*Bu is known to induce SET processes,[29] control reactions show the alkoxide alone is not the capable to promote these transformations. Extension to other π-extended systems such as phenanthrene and pyrene led to the deep coloured, NMR silent solutions suggesting a similar competing SET process (see figure S55 for optical evidence).

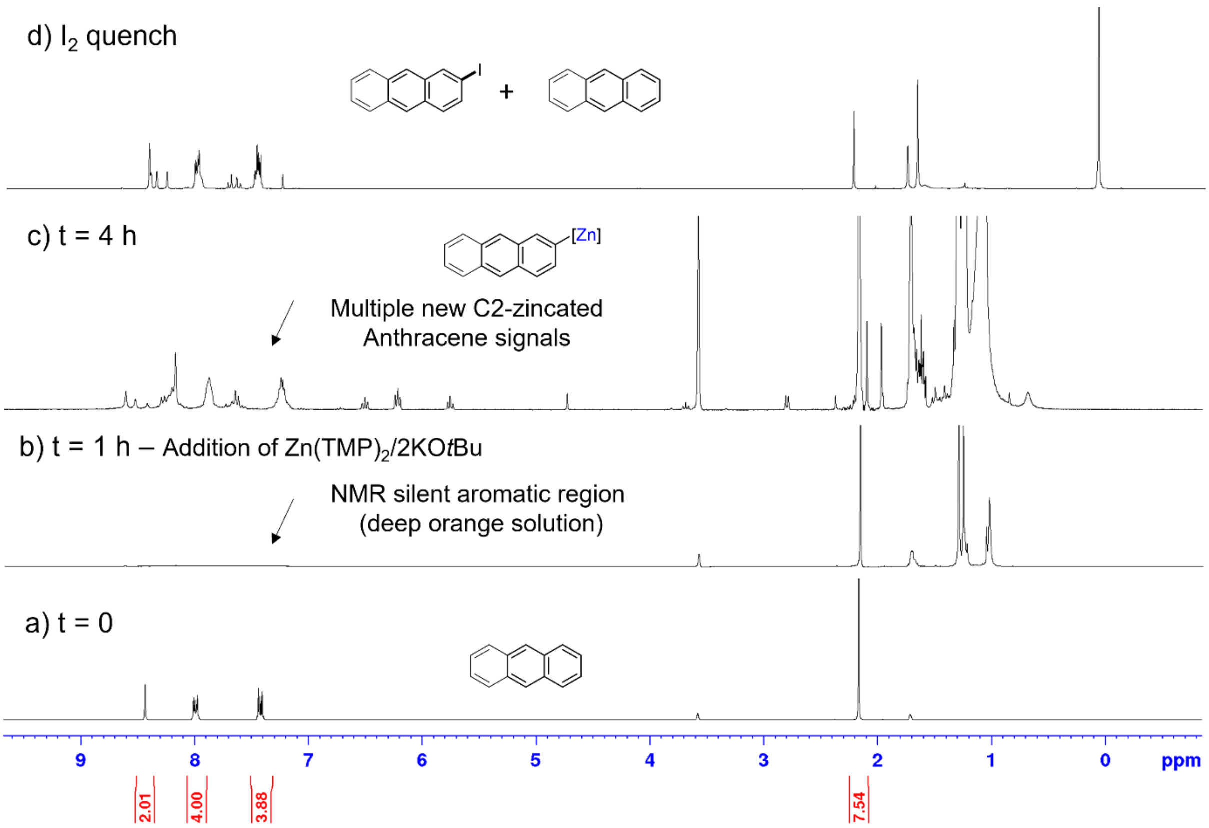

Figure S 54 Stacked ^{1}H-NMR spectra in d_8-THF of a) t = 0, anthracene and hexamethylbenzene internal standard ; b) t = 1h after addition of $Zn(TMP)_2$/2KO*t*Bu displaying NMR silent aromatic region ; c) t = 4h, emergence of aromatic signals assigned to zincated anthracene and formation of TMP(H); d) I_2 quench and formation of a 1:1 mixture of 2-iodoanthracene **2c** and re-formed anthracene starting material.

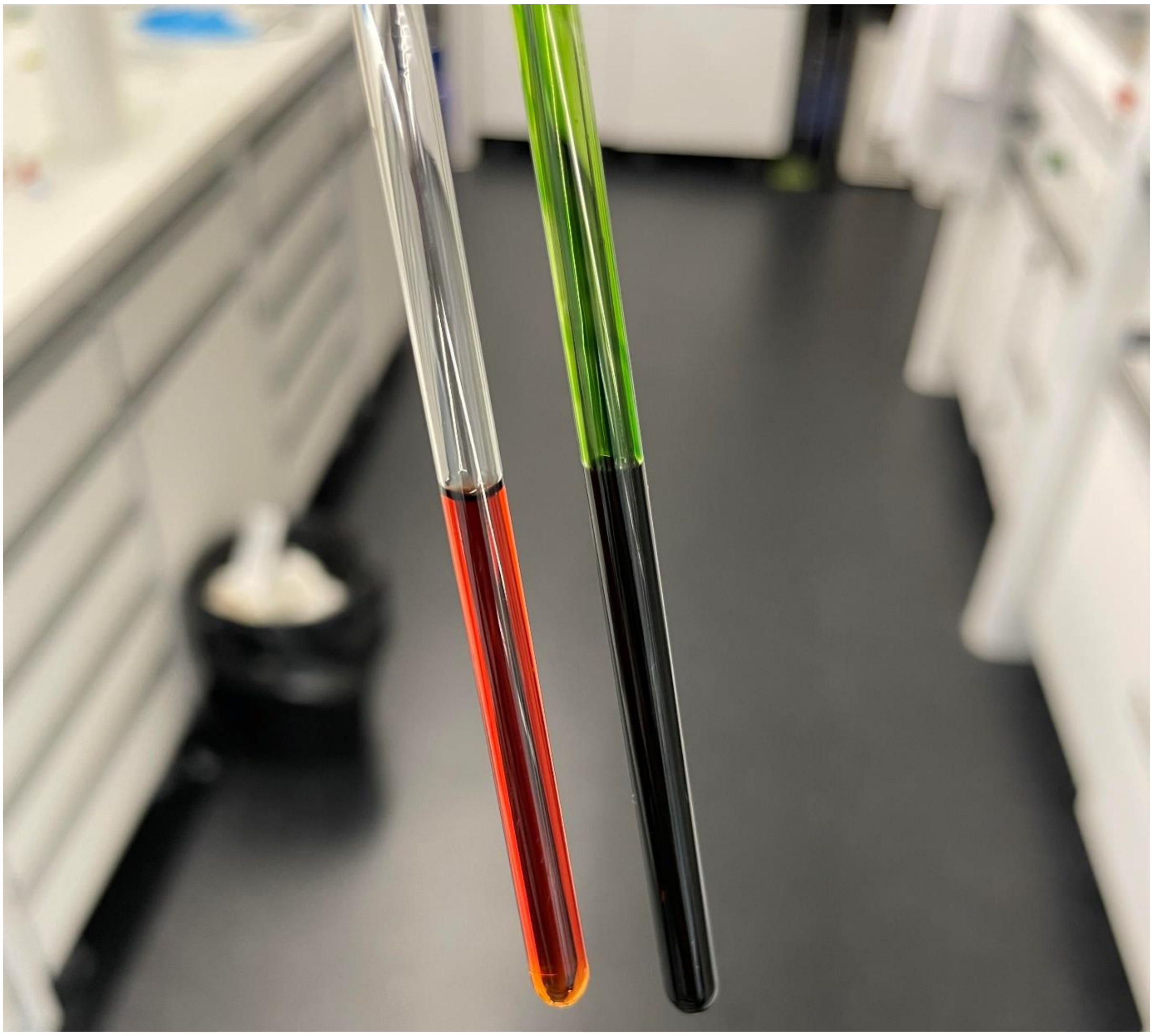

Figure S 55 Deep coloured solutions of anthracene (left, orange) and phenanthrene (right, dark green) after reaction with $Zn(TMP)_2$/2KO*t*Bu in d_8-THF.

4.3 *in-situ* zincation of 1,3-benzoxazole and generation of **3c** and **4**

In an argon flushed J Young's NMR tube, 1,3-benzoxazole (0.2 mmol, 24 mg) and hexamethylbenzene (approx. 5.4 mg) were dissolved in d_8-THF (0.5 mL). ^{1}H NMR recorded. To this solution was added $Zn(TMP)_2$ (0.1 mmol, 35 mg) and KO*t*Bu (0.2 mmol, 22 mg). ^{1}H NMR monitoring shows full conversion of 1,3-benzoxazole after 20 minutes and the emergence of a 1:1 mixture of $[(THF)_2KZn(2\text{-benzoxazolyl})(OtBu)_2]_2]$ (**3c**) and $[(THF)_2K(1,2\text{-}O\text{-}C_6H_4\text{-}NC)]_2$ (**4**) with concomitant formation of two equivalents of TMP(H). Comparison of the ^{1}H-NMR spectra from the independent synthesis of **3c** and **4** an exact match with the chemical shifts found in the *in-situ* generated mixture (Figure S57), ^{13}C-NMR also an exact match. NMR spectroscopy of **4** fits well with those reported for lithium 2-isocyanophenolate.[17]

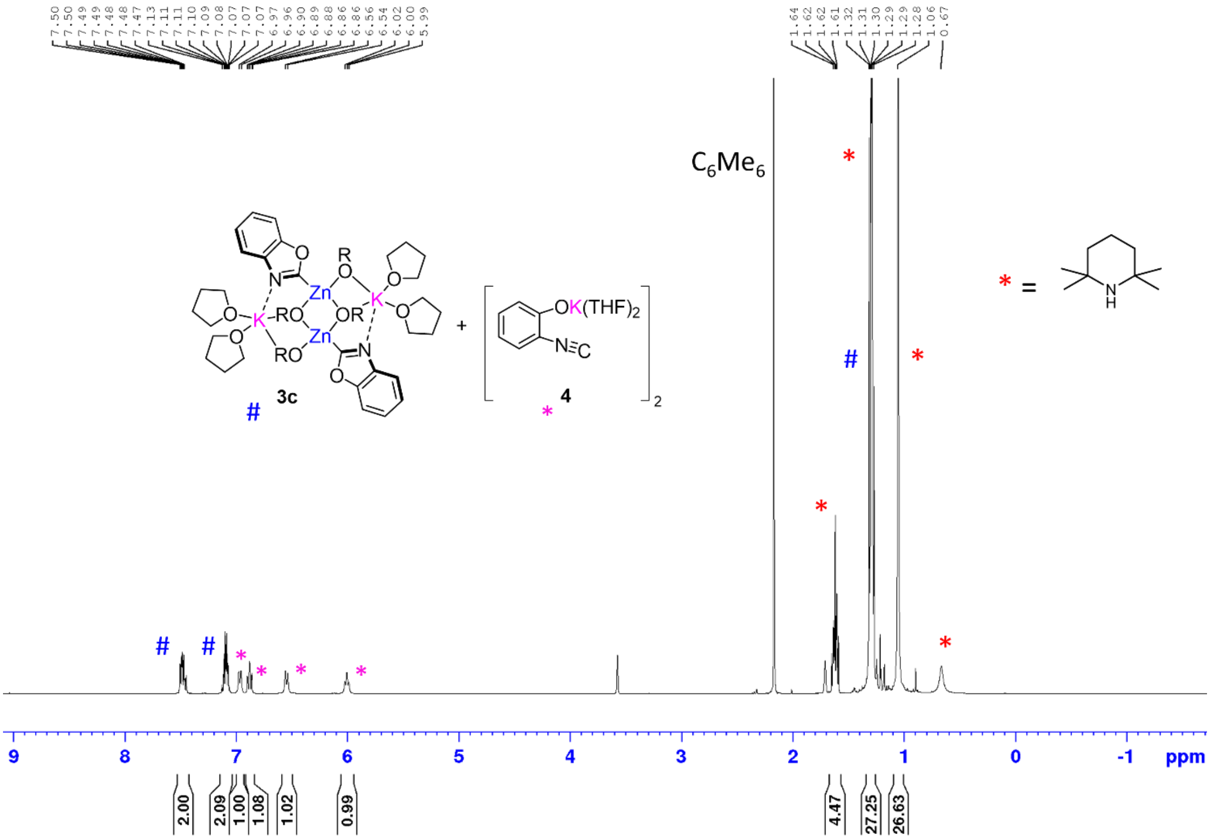

Figure S 56 1:1 mixture of **3c** and **4** formed from reaction of two equivalents of benzoxazole with Zn(TMP)$_2$/2KO*t*Bu in d$_8$-THF.

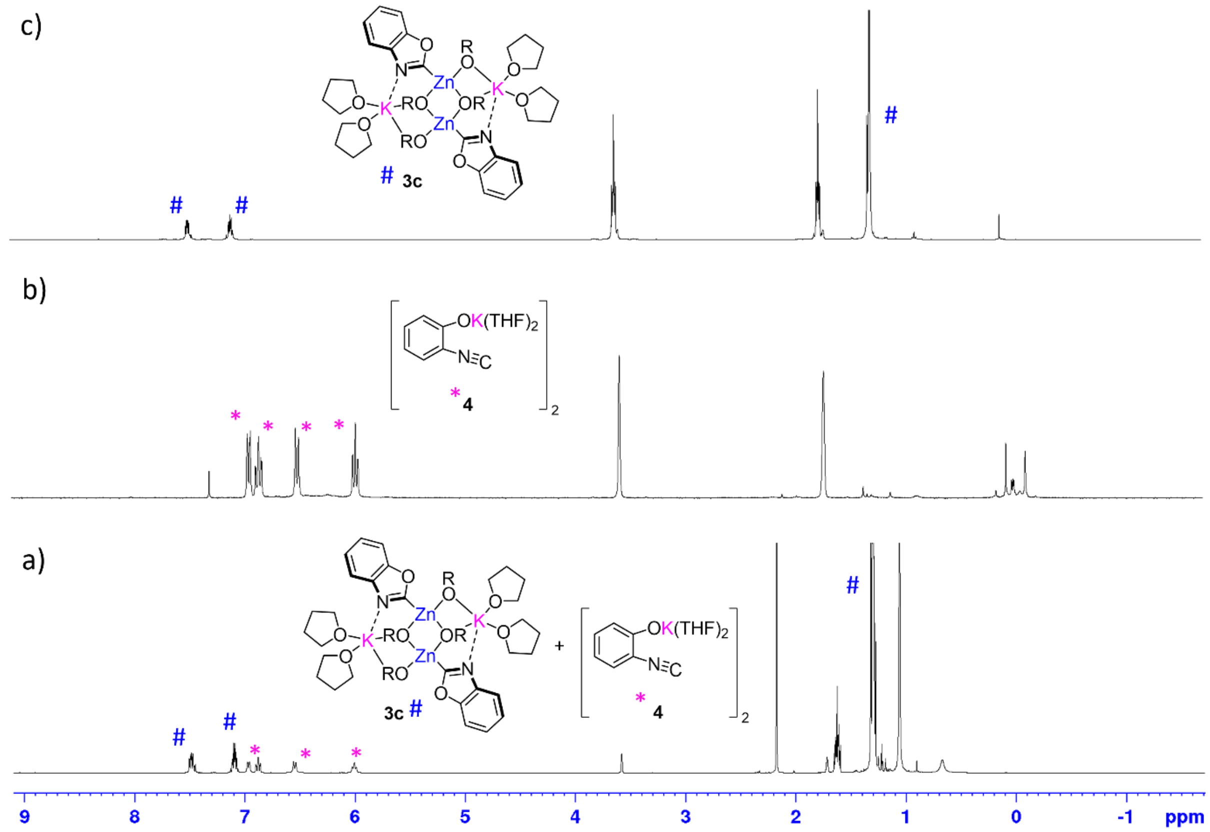

Figure S 57 Stacked ^{1}H-NMR spectra in d_8-THF of a) *in-situ* reaction of two equivalents of benzoxazole with $Zn(TMP)_2$/2KO*t*Bu in d_8-THF forming a 1:1 mixture of **3c** and **4**; b) isolated $[(THF)_2K(1,2\text{-}O\text{-}C_6H_4\text{-}NC)]_2$ (**4**) and c) $[(THF)_2KZn(2\text{-benzoxazolyl})(OtBu)_2]_2]$ (**3c**).

<u>4.4 Zincation of 1,3-benzothiazole and in-situ generation of **Id**</u>

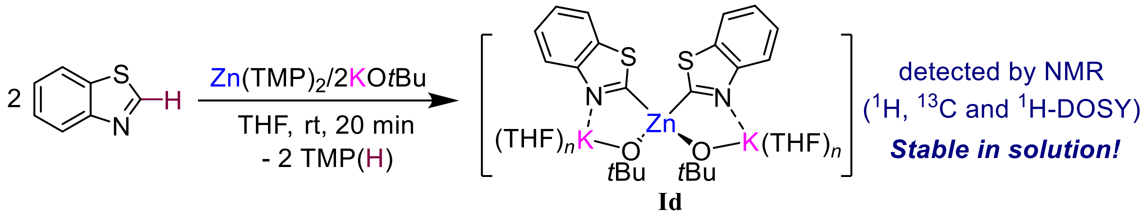

In an argon flushed J Young's NMR tube, 1,3-benzothiazole (0.2 mmol, 22 µL) and hexamethylbenzene (approx. 5.4 mg) were dissolved in d_8-THF (0.5 mL). To this solution was added $Zn(TMP)_2$ (0.1 mmol, 35 mg) and KO*t*Bu (0.2 mmol, 22 mg). ^{1}H NMR monitoring shows full conversion of 1,3-benzothiazole after 20 minutes at room temperature forming $[(THF)_nK_2Zn(2\text{-benzothiazolyl})_2(OtBu)_2]$ (**Id**) with concomitant formation of TMP(H).

^{1}H-DOSY NMR spectroscopic studies from an *in-situ* deprotonation of 2 equivalents of 1,3-benzothiazole with $Zn(TMP)_2$ and 2 equivalents of KO*t*Bu indicate the formation of a single molecular potassium zincate entity $[(THF)_nK_2Zn(2\text{-benzothiazolyl})_2(OtBu)_2]$ (**Id**) based on the independent diffusion coefficients of the chemical shifts in the ^{1}H-NMR spectra representing the zincated heterocyclic

moiety and the alkoxide fragment. Formation of **Id** proceeds with concomitant formation of 2 equivalents of TMP(H) present in the ^{1}H and ^{13}C NMR spectra.

[(THF)$_n$K$_2$Zn(2-benzothiazolyl)$_2$(O*t*Bu)$_2$] (**Id**): D = 4.33 x10^{-10} m^{2}s^{-1}.

^{1}H-NMR (300.1 MHz, D$_8$-THF, 298 K): δ / ppm = 7.89 (dd, J = 26.4 Hz, 7.0 Hz, 4H, Ar-H), 7.22-7.08 (m, 4H, Ar-H), 0.94 (s, 18H, 2 x O*t*Bu).

^{13}C{^{1}H}-NMR (101 MHz, D$_8$-THF, 298 K): δ / ppm = 211.8 (s, C$_q$-Zn), 158.1 (s, C$_q$), 138.9 (s, C$_q$), 123.4 (s, Ar, C-H), 121.9 (s, Ar, C-H), 121.6 (s, Ar, C-H), 120.9 (s, Ar, C-H), 68.0 (s, C$_q$, O*t*Bu), 35.6 (s, CH$_3$, O*t*Bu).

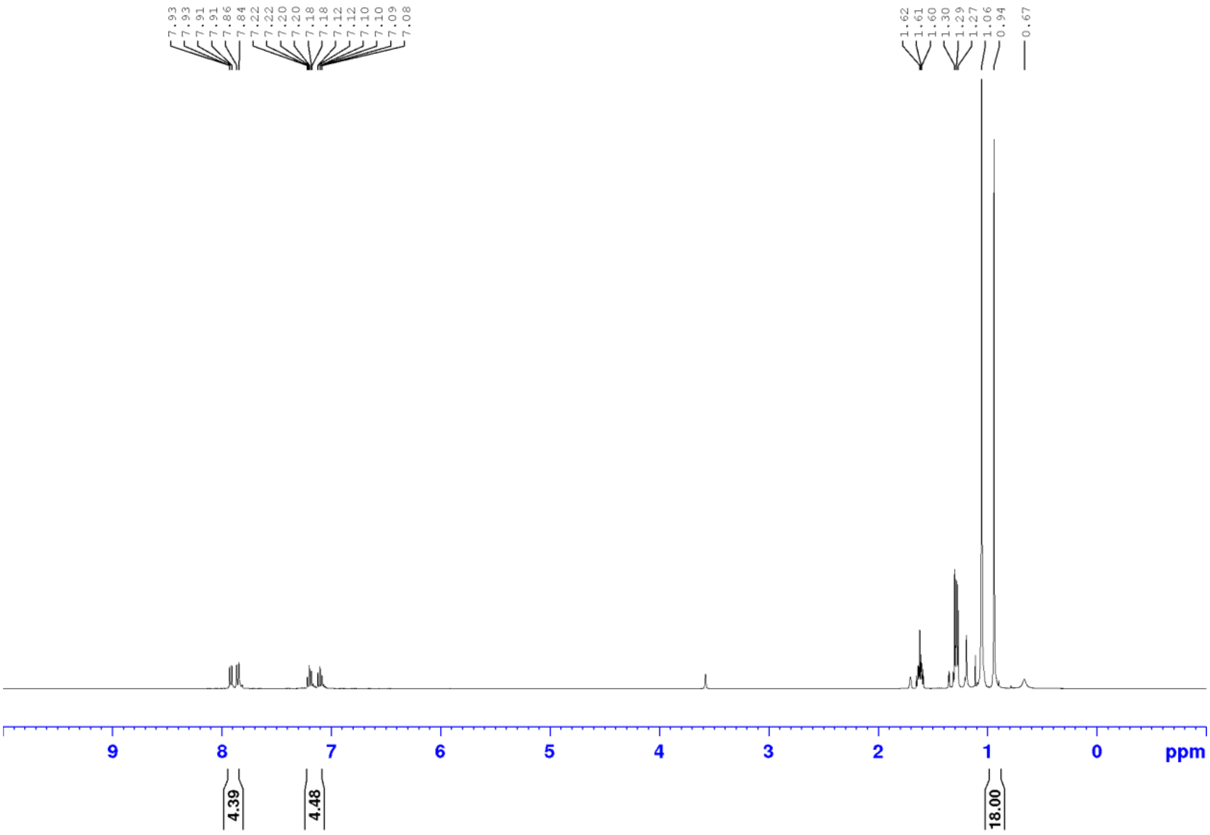

Figure S 58 ^{1}H-NMR spectra of [(THF)$_n$K$_2$Zn(2-benzothiazolyl)$_2$(O*t*Bu)$_2$] **Id** and TMP(H) in d$_8$-THF.

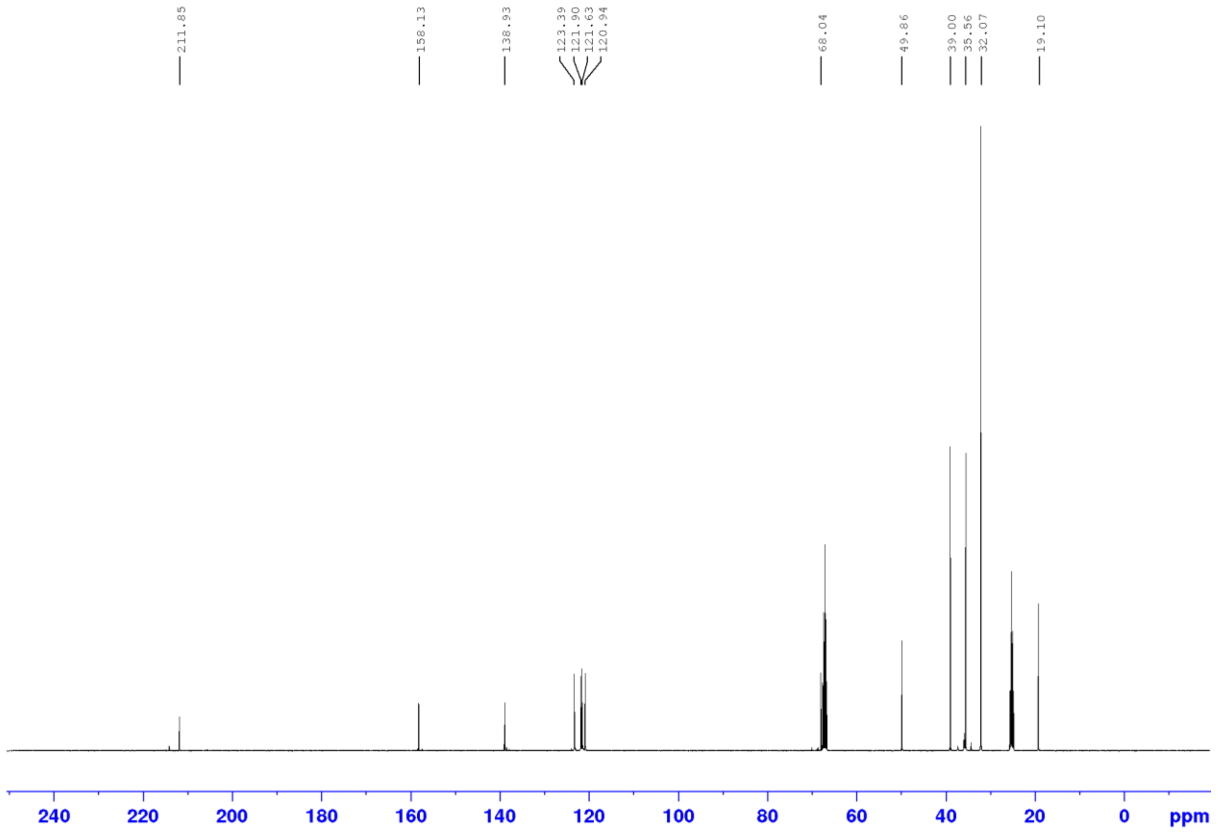

Figure S 59 $^{13}C\{^{1}H\}$-NMR} spectra of [(THF)$_n$K$_2$Zn(2-benzothiazolyl)$_2$(O*t*Bu)$_2$] **Id** and TMP(H) in d_8-THF.

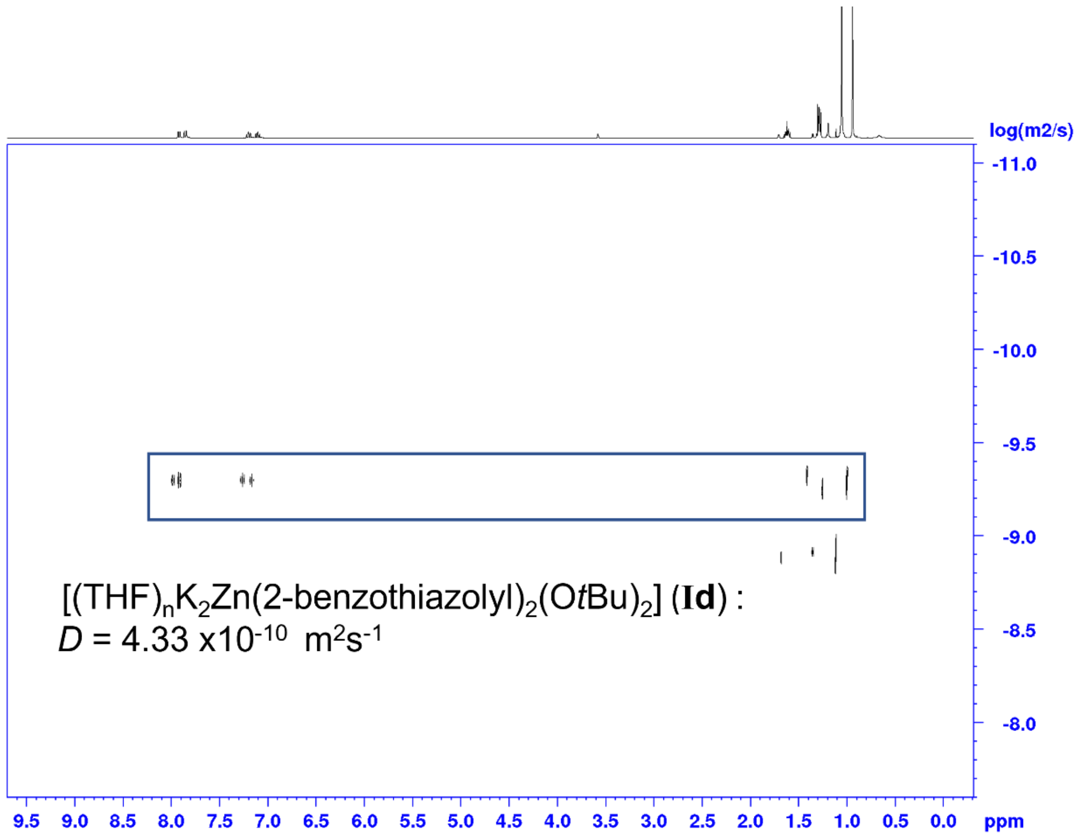

Figure S 60 ^{1}H-DOSY NMR spectra of [(THF)$_n$K$_2$Zn(2-benzothiazolyl)$_2$(O*t*Bu)$_2$] **Id** and TMP(H) in d_8-THF.

4.5 Negishi cross-coupling reaction via zincation of benzothiophene

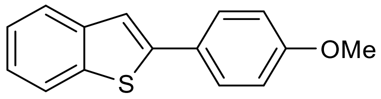

2-(4-methoxyphenyl)benzo[*b*]thiophene

In an argon flushed Schlenk flask, benzothiophene (0.72 mmol, 97 mg, 2 equiv), $Zn(TMP)_2$ (0.36 mmol, 130 mg, 1 equiv) and KO*t*Bu (0.72 mmol, 81 mg, 2 equiv) were dissolved in THF (2 mL). The reaction was left to stir for 20 minutes at room temperature affording a pale-yellow solution. The reaction mixture was then transferred via cannula to a J. Young's ampoule containing a THF (2 mL) solution of 4-iodoanisole (0.65 mmol, 152 mg, 1.8 equiv), $Pd(OAc)_2$ (5 mol%) and SPhos (10 mol%). The resulting dark brown suspension was stirred at 60 °C for 3h and then quenched with NH_4Cl (5 mL), organics extracted with EtOAc (3 x 10 mL), washed with brine (1 x 10 mL), dried over $MgSO_4$, filtered and hexamethylbenzene (10 mol%) was added as an internal standard. An aliquot of this reaction was taken, solvent removed *in-vacuo* and the resulting brown solid dissolved in $CDCl_3$. ^{1}H-NMR analysis revealed the quantitative (>95%) formation of 2-(4-methoxyphenyl)benzothiophene using hexamethylbenzene as an internal standard. Spectroscopic data was an identical match for those previously reported.[30]

^{1}H-NMR (300.1 MHz, D_8-THF, 298 K): δ / ppm = 7.81 (br d, J = 8.1 Hz, 1H, Ar-H), 7.75 (br d, J = 7.84, 1H, Ar-H), 7.65 (d, J = 8.2 Hz, 2H, Ar-H), 7.43 (s, 1H, Ar-H), 7.35-7.27 (m, 2H, Ar-H), 6.96 (d, J = 8.8 Hz, 2H, Ar-H), 3.86 (s, 3H, *OMe*).

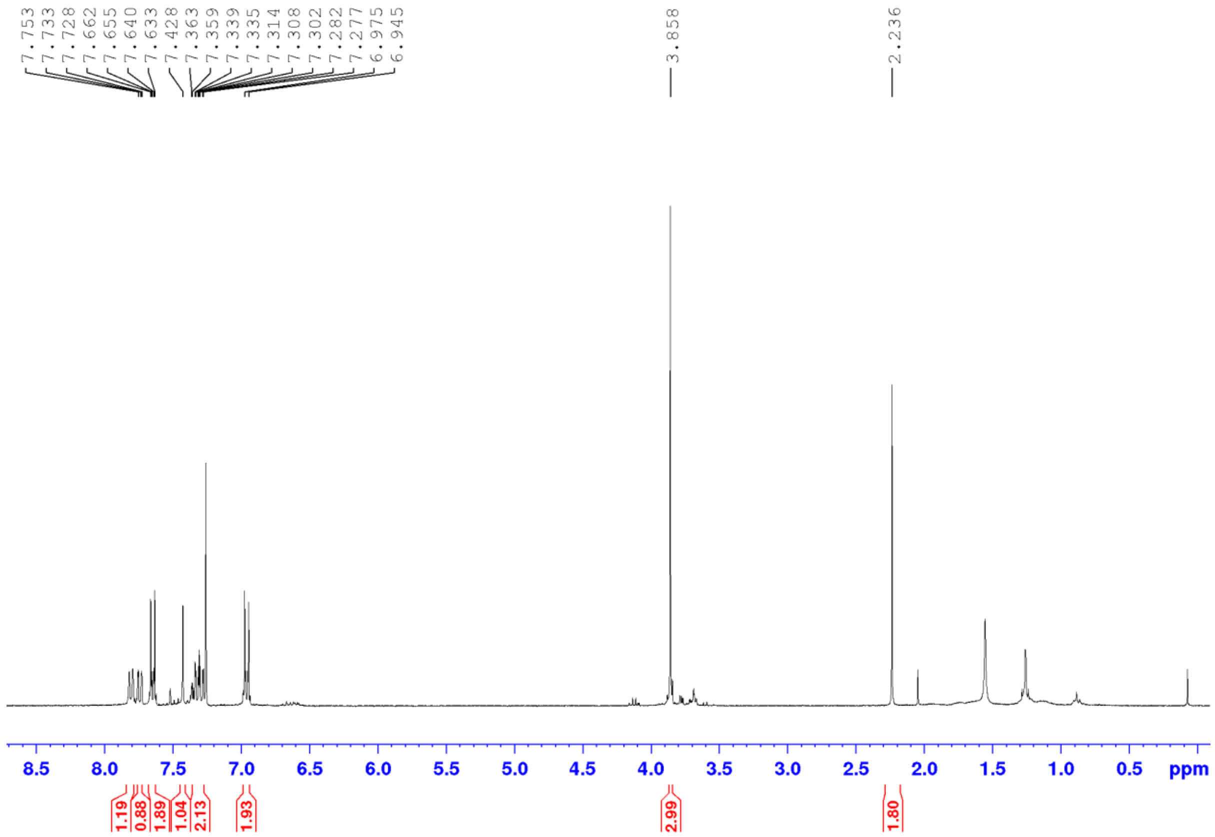

Figure S 61 ^{1}H-NMR of 2-(4-methoxyphenyl)benzothiophene in $CDCl_3$ with 10 mol% hexamethylbenzene as internal standard.

<u>**4.6** THF metalation and decomposition with Zn(TMP)$_2$/2KO*t*Bu</u>

Leaving a solution of Zn(TMP)$_2$/2KO*t*Bu in d$_8$-THF to stand at room temperature for 3 days in a sealed J. Youngs NMR tube in the glovebox was accompanied with a dramatic colour change from colourless to an intense bright purple solution (Figure S63). ^{1}H-NMR analysis of this solution indicated that all Zn-TMP signals had been converted to TMP(D) (Figure S62), indicating a possible deprotonative metalation reaction between the zinc base and the THF solvent. Note the absence of the N-H signal which for TMP(H) is found at 0.67 ppm (see Figure S56 and S58 for examples of TMP(H)) indicating a deprotonation of the deuterated solvent and not hydrolysis of the sample.

As described in section **3.4**, leaving our Zn(TMP)$_2$/2KO*t*Bu combination to stir in protic THF over the course of three days gives deep purple/black solution with a black deposit appearing on the Teflon stirrer bar. A work-up of this reaction in hexane with the addition of Lewis donor PMDETA afforded a

colourless crop of crystals which was analysed by a combination of NMR spectroscopy and X-ray crystallography revealing potassium zincate [(PMDETA)KZn(C_4H_5)(O*t*Bu)$_2$]$_2$ (**7**) formed in a 37% crystalline yield. In an effort to ascertain whether this zincate **7** was indeed the product of an intriguing decomposition of THF, the reaction was carried out again in d_8-THF, all solvent removed and subsequent deuterium-NMR analysis (Figure S64) in protic THF indicated deuterium incorporation into the C_4H_5 diene fragment displaying broad multiplets, at similar chemical shifts in the ^{2}H-NMR (6.76, 6.22, 5.11 and 4.68 ppm) to those observed in the ^{1}H-NMR spectrum of **7** (6.68, 6.23, 4.73 and 4.54 ppm).

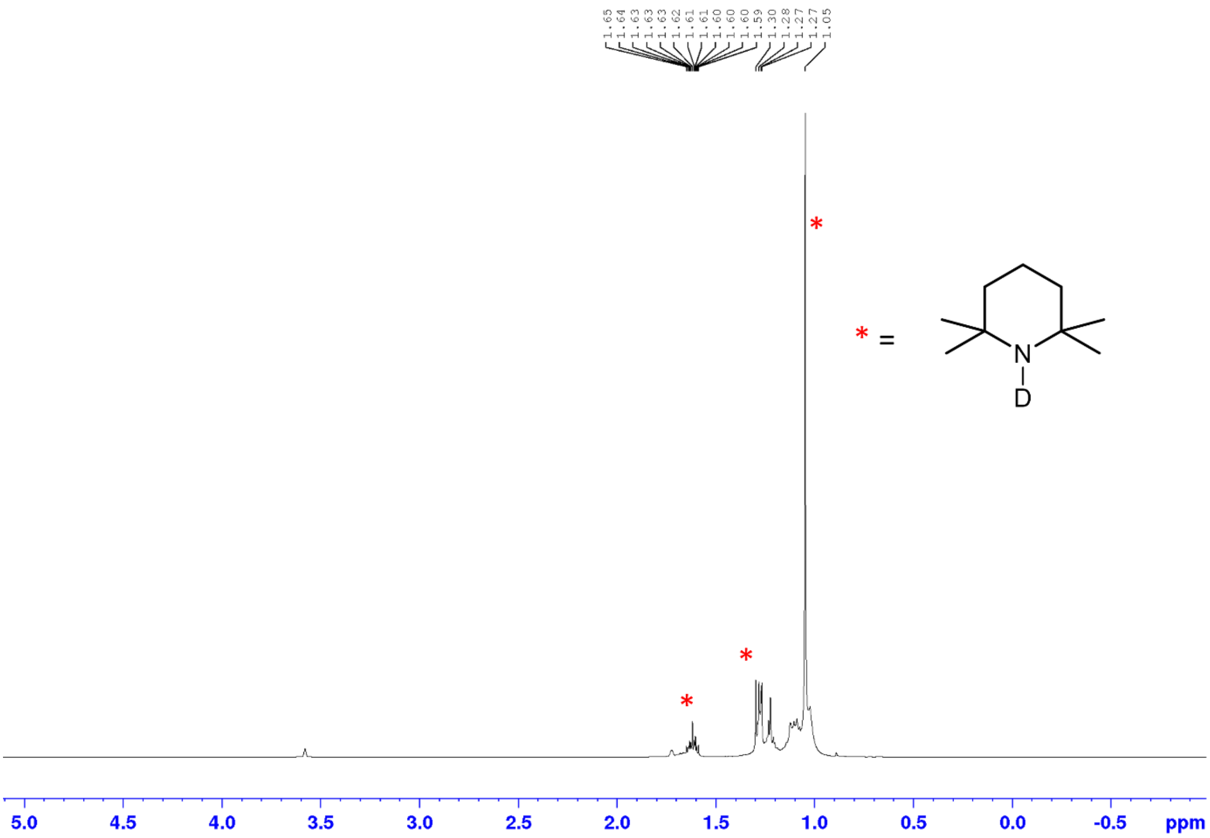

Figure S 62 ^{1}H-NMR spectra of a mixture of Zn(TMP)$_2$/2KO*t*Bu in d_8-THF after being left to stand at room temperature for 72h.

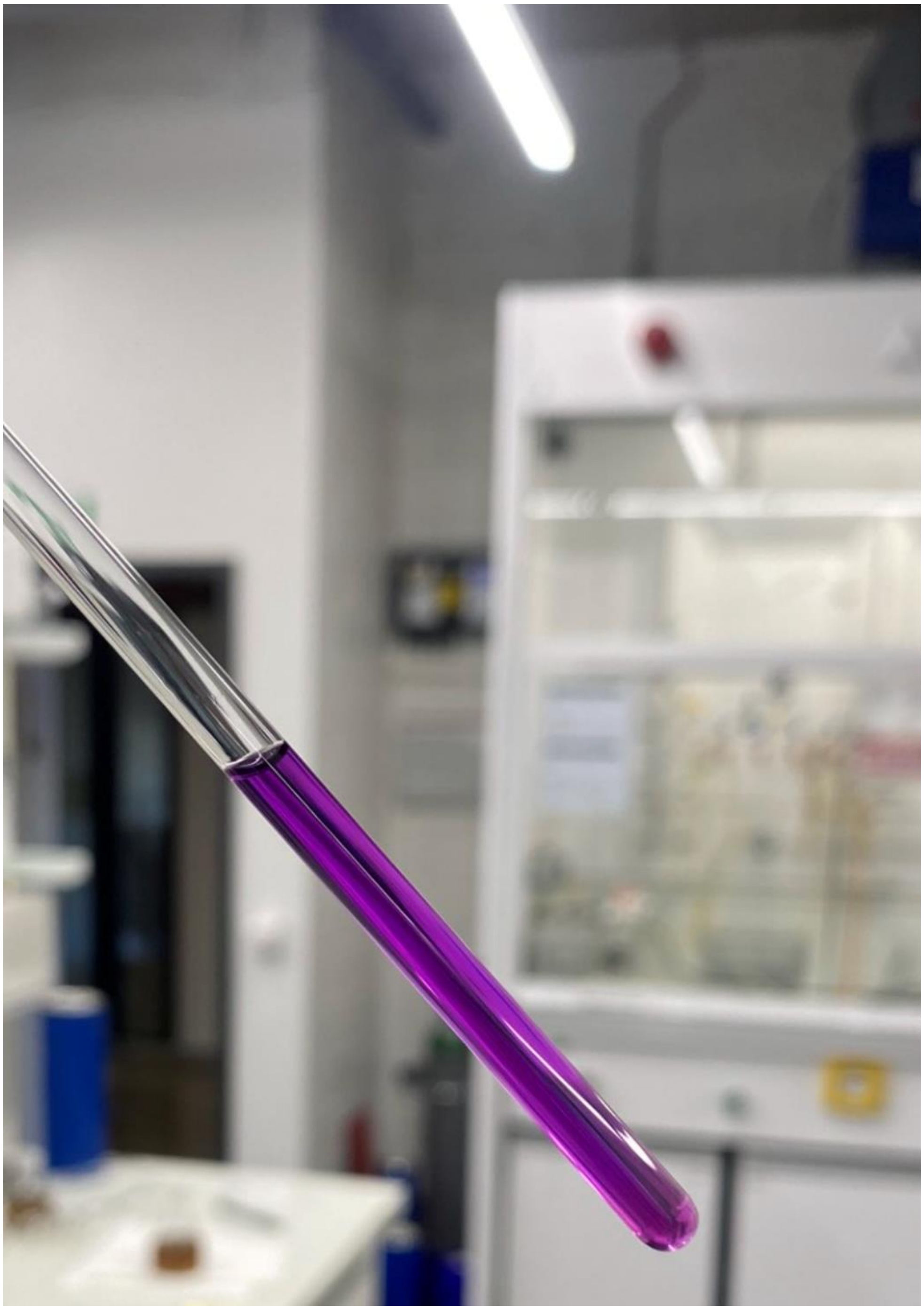

Figure S 63 Bright purple solution of a mixture of $Zn(TMP)_2/2KOtBu$ in d_8-THF after being left to stand at room temperature for 72h.

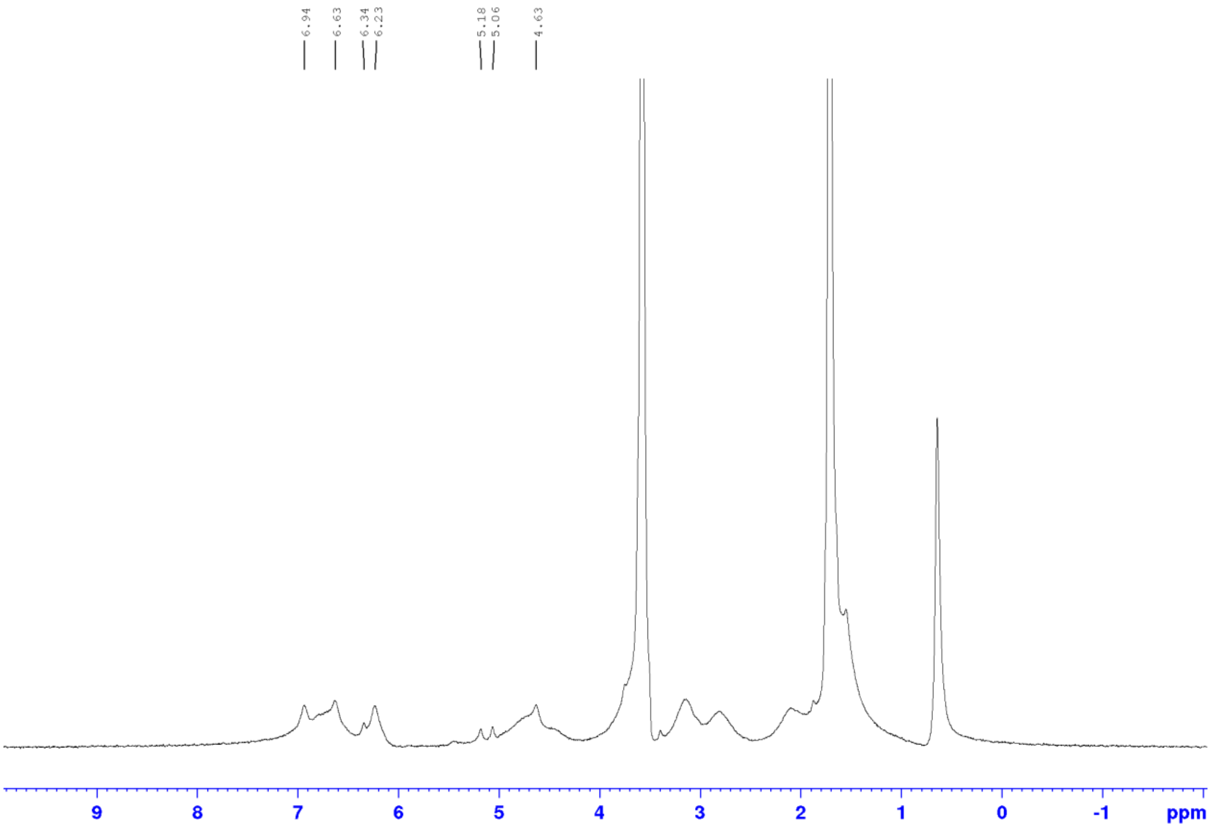

Figure S 64 D-NMR spectra of the crude mixture of a combination of $Zn(TMP)_2$/2KO*t*Bu in d_8-THF after being left to stand at room temperature for 72h.

5. References

[1] A. M. Borys, *Organometallics* **2023**, DOI 10.1021/acs.organomet.2c00535.

[2] D. Huang, D. Olivieri, Y. Sun, P. Zhang, T. R. Newhouse, *J. Am. Chem. Soc.* **2019**, *141*, 16249–16254.

[3] G. M. Sheldrick, *Acta Crystallogr. Sect. A Found. Crystallogr.* **2015**, *71*, 3–8.

[4] G. M. Sheldrick, *Acta Crystallogr. Sect. C Struct. Chem.* **2015**, *71*, 3–8.

[5] O. V. Dolomanov, L. J. Bourhis, R. J. Gildea, J. A. K. Howard, H. Puschmann, *J. Appl. Crystallogr.* **2009**, *42*, 339–341.

[6] Y. Gu, R. Martín, *Angew. Chem. Int. Ed.* **2017**, *56*, 3187–3190.

[7] H. Yang, Y. Li, M. Jiang, J. Wang, H. Fu, *Chem. Eur. J.* **2011**, *17*, 5652–5660.

[8] P. C. Meltzer, P. Blundell, H. Huang, S. Liu, Y. F. Yong, B. K. Madras, *Bioorganic Med. Chem.* **2000**, *8*, 581–590.

[9] Z. Wu, F. Wei, B. Wan, Y. Zhang, *J. Am. Chem. Soc.* **2021**, *143*, 4524–4530.

[10] J. C. Goeltz, C. P. Kubiak, *Organometallics* **2011**, *30*, 3908–3910.

[11] N. H. Chang, X. C. Chen, H. Nonobe, Y. Okuda, H. Mori, K. Nakajima, Y. Nishihara, *Org. Lett.* **2013**, *15*, 3558–3561.

[12] E. B. Stephens, K. E. Kinsey, J. F. Davis, J. M. Tour, *Macromolecules* **1993**, *26*, 3519–3532.

[13] J. M. L'Helgoual'ch, A. Seggio, F. Chevallier, M. Yonehara, E. Jeanneau, M. Uchiyama, F. Mongin, *J. Org. Chem.* **2008**, *73*, 177–183.

[14] J. Bergman, L. Venemalm, *J. Org. Chem.* **1992**, *57*, 2495–2497.

[15] V. L. Blair, D. C. Blakemore, D. Hay, E. Hevia, D. C. Pryde, *Tetrahedron Lett.* **2011**, *52*, 4590–4594.

[16] Q. Shi, S. Zhang, J. Zhang, V. F. Oswald, A. Amassian, S. R. Marder, S. B. Blakey, *J. Am. Chem. Soc.* **2016**, *138*, 3946–3949.

[17] O. Bayh, H. Awad, F. Mongin, C. Hoarau, L. Bischoff, F. Trécourt, G. Quéguiner, F. Marsais, F. Blanco, B. Abarca, R. Ballesteros, *J. Org. Chem.* **2005**, *70*, 5190–5196.

[18] R. Neufeld, D. Stalke, *Chem. Sci.* **2015**, *6*, 3354–3364.

[19] N. R. Judge, E. Hevia, *Angew. Chem. Int. Ed.* **2023**, *62*, DOI 10.1002/anie.202303099.

[20] D. Anderson, A. Tortajada, E. Hevia, *Angew. Chem. Int. Ed.* **2023**, DOI 10.1002/anie.202218498.

[21] P. E. Krach, A. Dewanji, T. Yuan, M. Rueping, *Chem. Commun.* **2020**, *56*, 6082–6085.

[22] P. Benrath, M. Kaiser, T. Limbach, M. Mondeshki, J. Klett, *Angew. Chem. Int. Ed.* **2016**, *55*, 10886–10889.

[23] J. Klett, *Chem. – A Eur. J.* **2021**, *27*, 888–904.

[24] J. Kocher, N. R. Judge, E. Hevia, *Helv. Chim. Acta* **2024**, *3*, 2–11.

[25] D. Shimizu, A. Osuka, *Chem. Sci.* **2018**, *9*, 1408–1423.

[26] A. Shiozuka, K. Sekine, Y. Kuninobu, *Synth.* **2022**, *54*, 2330–2339.

[27] S. Samanta, S. Mondal, *Asian J. Org. Chem.* **2021**, *10*, 2503–2520.

[28] K. Seto, T. Nakayama, B. Uno, *J. Phys. Chem. B* **2013**, *117*, 10834–10845.

[29] E. C. Ashby, A. B. Goel, R. N. DePriest, *J. Org. Chem.* **1981**, *46*, 2429–2431.

[30] S. Tamba, Y. Okubo, S. Tanaka, D. Monguchi, A. Mori, *J. Org. Chem.* **2010**, *75*, 6998–7001.
